# Supplementary material for: Synthesis of Ethynyl Trifluoromethyl Sulfide and Its Application to the Synthesis of CF3S-Containing Triazoles
Source: Molecules. 2025 May 28;30(11):2358. doi: 10.3390/molecules30112358 (PMC12156333; doi:10.3390/molecules30112358)
Supplement: Supplementary file 1 [file molecules-30-02358-s001.zip › molecules-3622829-supplementary.pdf]

Synthesis of Ethynyl Trifluoromethyl Sulfide and Its Application to the Synthesis of CF_3S-Containing Triazoles

Alejandra Riesco-Domínguez,[a] Hussein Hammoudeh,[a] Daniel Blanco-Ania[a] and F. P. J. T. Rutjes*[a]

[a]Radboud University, Institute for Molecules and Materials, Heyendaalseweg 135, 6525 AJ Nijmegen, the Netherlands

floris.rutjes@ru.nl

Table of Contents

1.	General Procedure for the Synthesis of Azides **8h–k**	S3
2.	General Procedure for the Synthesis of Ethynyl Trifluoromethyl Sulfide **3**	S4
3.	General Procedure for the Synthesis of 1,4-Disubstituted-1*H*-1,2,3-Triazoles **5a–o**	S5
4.	References and Notes	S11
5.	NMR Data	S12

General Procedure for the Synthesis of Aromatic Azides 8h–k

The corresponding aniline **9h–k** (1.0 mmol) was added to a solution of TsOH·H_2O (1.62 g, 9.0 mmol) in H_2O (9 mL). After stirring for 1 min, anhydrous $NaNO_2$ (0.621 g, 9.0 mmol) was added gradually over 5 min. The resulting solution was then stirred for a period between 2–60 min until the starting amine disappeared (reactions were monitored by TLC). Anhydrous NaN_3 (0.104 g, 1.6 mmol) was added to the resulting solution and an immediate emission of N_2 was observed. The solid aromatic azides (**8h** and **8i**) were filtered off, washed with H_2O (50 mL) and dried in vacuo whereas the oily azides (**8j** and **8k**) were extracted with AcOEt (3 × 10 mL) and dried over Na_2SO_4. The suspension was filtered off and dried under reduced pressure.

Scheme S1 *Synthesis of Aromatic Azides **8h–k** from Anilines **9h–k**.*

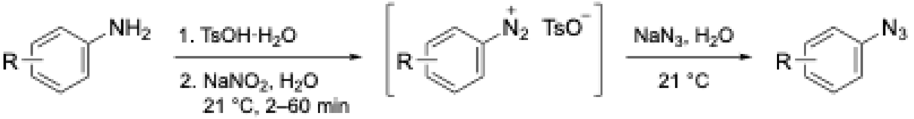

9h–k 10h–k 8h–k

1-Azido-3-methoxybenzene 8h[1]

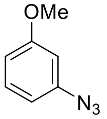

According to the general procedure, the reaction of aniline **9h** (123 mg, 1.0 mmol) afforded azide **8h** (144.7 mg, 0.97 mmol). **^{1}H NMR** [400 MHz, δ (ppm), $CDCl_3$]: 7.25 (t, *J* = 8.1 Hz, 1 H), 6.67 (ddd, *J* = 8.3, 2.4, 0.8 Hz, 1 H), 6.65 (ddd, *J* = 8.0, 2.1, 0.9 Hz, 1 H), 6.55 (t, *J* = 2.2 Hz, 1 H), 3.80 (s, 3 H). **Yield**: 97%.

3-Azidobenzonitrile 8i[2]

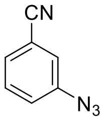

According to the general procedure, the reaction of aniline **9i** (118.1 mg, 1.0 mmol) afforded azide **8i** (136.9 mg, 0.95 mmol). **^{1}H NMR** [400 MHz, δ (ppm), $CDCl_3$]: 7.65–7.56 (m, 2 H), 7.28–7.25 (m, 1 H), 7.22 (td, *J* = 7.7, 1.0 Hz, 1 H). **Yield**: 95%.

1-Azido-2-methoxybenzene 8j[1]

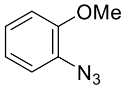

According to the general procedure, the reaction of aniline **9j** (123.2 mg, 1.0 mmol) afforded azide **8j** (141.7 mg, 0.95 mmol). **^{1}H NMR** [400 MHz, δ (ppm), $CDCl_3$]: 7.10 (td, *J* = 7.8, 1.7 Hz, 1 H), 7.02 (dd, *J* = 7.8, 1.7 Hz, 1 H), 6.97–6.88 (m, 2 H), 3.88 (s, 3 H, OC*H*$_3$). **Yield**: 95%.

2-Azidobenzonitrile 8k[3]

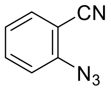

According to the general procedure, the reaction of aniline **9k** (118.1 mg, 1.0 mmol) afforded azide **8k** (115.3 mg, 0.80 mmol). **^{1}H NMR** [400 MHz, δ (ppm), $CDCl_3$]: 7.50–7.40 (m, 2 H), 7.30–7.24 (m, 2 H). **Yield**: 80%.

Azides **8b–g** were commercially available and azides **8a**,[4] **8l**[5] and **8m**[6] were previously prepared in our research group according to procedures published in the literature.

General Procedure for the Synthesis of Ethynyl Trifluoromethyl Sulfide 3

In a sealed vial under a nitrogen atmosphere, 2-chloroethyl trifluoromethyl sulfide (**1**; 100 mg, 0.608 mmol) was dissolved in CH_2Cl_2 (1 mL). The solution was cooled to 0 °C and subsequently KO^{t}Bu (790 µL, 0.790 mmol, 1.3 equiv, 1.0 M solution in THF) was slowly added. The reaction mixture was stirred at 21 °C for 90 min to form trifluoromethyl vinyl sulfide (**2**). Then, Br_2 (34 µL, 107 mg, 0.668 mmol, 1.1 equiv) in CH_2Cl_2 (1 mL) and added at 21 °C to the solution containing alkene **2**. The reaction mixture was stirred for 1 h, until the orange color of the mixture turned to a pale-yellow color, to afford the CF_3S-dibromo derivative **6**. Subsequently, the mixture was filtered and the CH_2Cl_2 was evaporated under reduced pressure in order to reduce 50% of the volume of the mixture. Then, the reaction mixture was cooled to 0 °C, KO^{t}Bu (790 µL, 0.790 mmol, 1.3 equiv, 1.0 M solution in THF) was slowly added and the reaction mixture was stirred at 21 °C for 90 min to afford alkene **7**. Finally, NaHMDS (517 µL, 1.034 mmol, 1.7 equiv, 2.0 M solution in THF) was slowly added and stirred for 1 h, to give CF_3S–C≡CH (**3**). ^{1}H NMR and ^{19}F NMR were checked after every reaction step and used for the final characterization of CF_3S–C≡CH (**3**).

Trifluoromethyl Vinyl Sulfide 2[7]

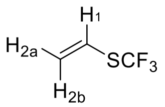

^{1}H NMR [400 MHz, δ (ppm), THF-d_8]: 6.54 (dd, J = 16.5, 9.4 Hz, 1 H, 1-C*H*), 5.72 (dq, J = 9.4, 1.5 Hz, 1 H, 2-CH_a), 5.70 (d, J = 16.5, 1 H, 2-CH_b). **^{13}C NMR** [101 MHz, δ (ppm), THF-d_8]: 129.8 (q, J = 306.5 Hz, SCF_3), 124.4 (q, J = 1.0 Hz, 2-*C*), 121.3 (q, J = 3.2 Hz, 1-*C*). **^{19}F NMR** [377 MHz, δ (ppm), THF-d_8]: −43.6.

1,2-Dibromoethyl Trifluoromethyl Sulfide 6

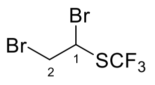

^{1}H NMR [400 MHz, δ (ppm), $CDCl_3$]: 5.46 (dd, *J* = 7.1, 5.7 Hz, 1 H, 1-C*H*), 4.00 (ddq, *J* = 11.4, 5.8, 0.5 Hz, 1 H, 2-C*H*H), 3.91 (ddq, *J* = 11.4, 7.1, 0.6 Hz, 1 H, 2-CH*H*). **^{13}C NMR** [126 MHz, δ (ppm), CD_2Cl_2/THF-*d*$_8$]: 130.37 (q, *J* = 309.1 Hz, SCF_3), 48.3 (1-*C*), 37.0 (2-*C*). **^{19}F NMR** [377 MHz, δ (ppm), $CDCl_3$]: −40.9.

1-Bromovinyl Trifluoromethyl Sulfide 7

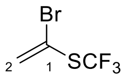

^{1}H NMR [500 MHz, δ (ppm), CD_2Cl_2/THF-*d*$_8$]: 6.56 (d, *J* = 2.3 Hz, 1 H, 2-C*H*H), 6.41 (dq, *J* = 2.3, 0.8 Hz, 1 H, 2-CH*H*). **^{13}C NMR** [126 MHz, δ (ppm), CD_2Cl_2/THF-*d*$_8$]: 138.2 (q, *J* = 1.3 Hz, 2-*C*), 130.0 (d, *J* = 310.5 Hz, SCF_3), 112.3 (q, *J* = 2.9 Hz, 1-*C*). **^{19}F NMR** [377 MHz, δ (ppm), $CDCl_3$]: −42.5.

Ethynyl Trifluoromethyl Sulfide 3

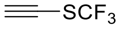

^{1}H NMR [400 MHz, δ (ppm), $CDCl_3$]: 3.33 (s, 1 H, C*H*). **^{19}F NMR** [377 MHz, δ (ppm), $CDCl_3$]: −43.2.

General Procedure for the Synthesis of 1,4-Disubstituted-1*H*-1,2,3-Triazoles 5a–o

A solution containing a mixture of $CF_3S–C≡CH$ (**3**; 3.0 equiv) and the corresponding azide **8** (1.0 equiv) in CH_2Cl_2/THF was added to a solution of $CuSO_4·5H_2O$ (2.5 mg, 0.01 mmol), sodium ascorbate (4 mg, 0.02 mmol) and benzoic acid (12 mg, 0.1 mmol) in ^{t}BuOH/H_2O (1:2 v/v, 1.0 mL) in a 4 mL vial. Then, CH_2Cl_2 was added in order to fill the vial completely. The resultant mixture was stirred for the stated time at the indicated temperature for every reaction (reactions were followed by ^{1}H and ^{19}F NMR). The reaction mixture was then quenched with H_2O (20 mL) and extracted with CH_2Cl_2 (3 × 15 mL). The combined organic layers were washed with H_2O and brine, dried over anhydrous Na_2SO_4, filtered off and concentrated in vacuo. The crude product was purified by column chromatography (heptane/AcOEt, 4:1) to afford the corresponding triazoles **5a–p**.

1-(4-Nitrophenyl)-4-[(trifluoromethyl)sulfanyl]-1*H*-1,2,3-triazole 5a

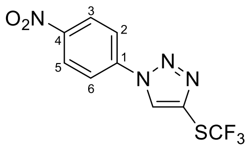

According to the general procedure, the reaction of 1-azido-4-nitrobenzene **8a** (21 mg, 0.13 mmol) with $CF_3S–C≡CH$ (**3**) at 30 °C for 16 h afforded triazole **5a** (46.4 mg, 0.16 mmol) as a yellow-brown solid. **^{1}H NMR** [400 MHz, δ (ppm),

$CDCl_3$]: δ 8.51–8.44 (m, 2 H, 3-C*H* + 5-C*H*), 8.42 (s, 1 H, C*H*), 8.05–7.98 (m, 2 H, 2-C*H* + 6-C*H*). **^{13}C NMR** [101 MHz, δ (ppm), $CDCl_3$]: 148.0 (4-*C*), 140.5 (1-*C*), 132.0 (*C*–S, indirect observation), 128.33 (*C*H), 128.32 (q, *J* = 310.0 Hz, SCF_3), 125.9 (3-*C* + 5-*C*), 121.1 (2-*C* + 6-*C*). **^{19}F NMR** [377 MHz, δ (ppm), $CDCl_3$]: –42.5. **FTIR** [$\bar{\nu}$ (cm^{-1})]: 2924, 1599, 1519, 1348, 1144, 1102, 907, 733. ***R*F**: 0.32 (heptane/AcOEt, 4:1). **Yield**: 82%.

1-(4-Fluorophenyl)-4-[(trifluoromethyl)sulfanyl]-1*H*-1,2,3-triazole 5b

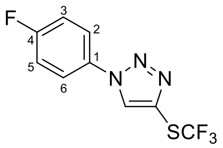

According to the general procedure, the reaction of 1-azido-4-fluorobenzene **8b** (250 µL, 0.125 mmol, 0.5 M solution in ^{t}BuOMe) with CF_3S–C≡CH (**3**) at 60 °C for 16 h afforded triazole **5b** (28.0 mg, 0.106 mmol) as a brown solid. **^{1}H NMR** [400 MHz, δ (ppm), $CDCl_3$]: 8.26 (s, 1 H, C*H*), 7.79–7.67 (m, 2 H, 2-C*H* + 6-C*H*), 7.31–7.22 (m, 2 H, 3-C*H* + 5-C*H*). **^{13}C NMR** [101 MHz, δ (ppm), $CDCl_3$]: 163.1 (d, *J* = 250.6 Hz, 4-*C*), 132.7 (1-C), 130.8 (*C*–S), 128.6 (*C*H), 128.4 (q, *J* = 309.7 Hz, SCF_3), 123.0 (d, *J* = 8.8 Hz, 2-*C* + 6-*C*), 117.2 (d, *J* = 23.5 Hz, 3-*C* + 5-*C*). **^{19}F NMR** [377 MHz, δ (ppm), $CDCl_3$]: –42.7 (SC*F*$_3$). **FTIR** [$\bar{\nu}$ (cm^{-1})]: 3125, 1520, 1243, 1146, 1122, 839. **HRMS** [ESI (m/z)] calcd for ($C_9H_5F_4N_3S$ + H)$^+$ = 264.02186, found 264.02227 (|Δ| = 1.59 ppm). ***R*F**: 0.42 (heptane/AcOEt, 4:1). **Yield**: 85%.

1-[4-(Trifluoromethyl)phenyl]-4-[(trifluoromethyl)sulfanyl]-1*H*-1,2,3-triazole 5c

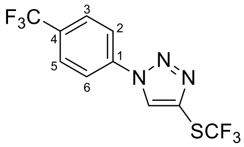

According to the general procedure, the reaction of 1-azido-4-(trifluoromethyl)benzene **8c** (250 µL, 0.125 mmol, 0.5 M solution in ^{t}BuOMe) with CF_3S–C≡CH (**3**) at 50 °C for 16 h afforded triazole **5c** (31.3 mg, 0.10 mmol) as a yellow solid. **^{1}H NMR** [400 MHz, δ (ppm), $CDCl_3$]: 8.38 (s, 1 H, *CH*), 7.97–7.89 (m, 2 H, 2-C*H* + 6-C*H*), 7.88–7.82 (m, 2 H, 3-C*H* + 5-C*H*). **^{13}C NMR** [101 MHz, δ (ppm), $CDCl_3$]: 138.8 (1-*C*), 131.9 (q, *J* = 33.4 Hz, 4-*C*), 131.4 (*C*–S), 128.37 (q, *J* = 309.9 Hz, SCF_3), 128.37 (CH), 127.5 (q, *J* = 3.7 Hz, 3-*C* + 5-*C*), 123.5 (q, *J* = 272.4 Hz, C*F*$_3$), 120.9 (2-*C* + 6-*C*). **^{19}F NMR** [377 MHz, δ (ppm), $CDCl_3$]: –42.6 (SC*F*$_3$), –62.8 (C*F*$_3$). **FTIR** [$\bar{\nu}$ (cm^{-1})]: 3117, 1335, 1151, 1106, 844. **HRMS** [ESI (m/z)] calcd for ($C_{10}H_5F_6N_3S$ + H)$^+$ = 314.01866, found 314.02077 (|Δ| = 2.11 mmu). ***R*F**: 0.50 (heptane/AcOEt, 4:1). **Yield**: 80%.

1-(4-Methylphenyl)-4-[(trifluoromethyl)sulfanyl]-1*H*-1,2,3-triazole 5d[8]

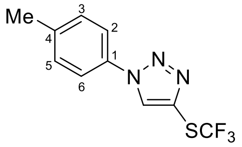

According to the general procedure, the reaction of 1-azido-4-methylbenzene **8d** (250 μL, 0.125 mmol, 0.5 M solution in ^{t}BuOMe) with CF_3S–C≡CH (**3**) at 70 °C for 48 h afforded triazole **5d** (28.0 mg, 0.108 mmol) as a brown solid. ^{1}**H NMR** [400 MHz, δ (ppm), $CDCl_3$]: 8.26 (s, 1 H, C*H*), 7.64–7.59 (m, 2 H, 2-C*H* + 6-C*H*), 7.38–7.32 (m, 2 H, 3-C*H* + 5-C*H*), 2.44 (s, 3 H, CH_3). ^{13}**C NMR** [101 MHz, δ (ppm), $CDCl_3$]: 140.1 (4-*C*), 134.2 (1-C), 130.6 (3-*C* + 5-*C*), 130.3 (*C*–S), 128.46 (q, *J* = 309.7 Hz, SCF_3), 128.45 (*C*H), 120.7 (2-*C* + 6-*C*), 21.3 (*C*H_3). ^{19}**F NMR** [377 MHz, δ (ppm), $CDCl_3$]: –42.8. **FTIR** [$\tilde{\nu}$ (cm^{-1})]: 2967, 1143, 1119, 1039, 817. **HRMS** [ESI (m/z)] calcd for $(C_{10}H_8F_3N_3S + H)^+$ = 260.04693, found 260.04638 (|Δ| = 2.11 ppm). ***R*F**: 0.44 (heptane/AcOEt, 4:1). **Yield**: 86%.

1-(4-Methoxyphenyl)-4-[(trifluoromethyl)sulfanyl]-1*H*-1,2,3-triazole 5e[9]

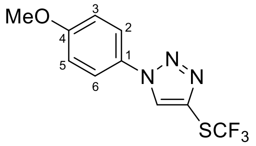

According to the general procedure, the reaction of 1-azido-4-methoxybenzene **8e** (250 μL, 0.125 mmol, 0.5 M solution in ^{t}BuOMe) with CF_3S–C≡CH (**3**) at 50 °C for 72 h afforded triazole **5e** (7.0 mg, 0.025 mmol) as a yellow-brown solid. ^{1}**H NMR** [400 MHz, δ (ppm), $CDCl_3$]: 8.21 (s, 1 H, C*H*), 7.67–7.62 (m, 2 H, 2-C*H* + 6-C*H*), 7.10–7.01 (m, 2 H, 3-C*H* + 5-C*H*), 3.89 (s, 3 H, OCH_3). ^{13}**C NMR** [101 MHz, δ (ppm), $CDCl_3$]: 160.5 (4-C), 130.1 (1-*C*), 129.7 (*C*–S), 128.4 (*C*H), 128.3 (q, *J* = 309.6 Hz, SCF_3), 122.4 (2-*C* + 6-*C*), 115.0 (3-*C* + 5-C), 55.7 (O*C*H_3). ^{19}**F NMR** [377 MHz, δ (ppm), $CDCl_3$]: –42.8. **FTIR** [$\tilde{\nu}$ (cm^{-1})]: 2926, 1522, 1261, 1145, 1121, 830. **HRMS** [ESI (m/z)] calcd for $(C_{10}H_8F_3N_3OS + H)^+$ = 276.04184, found 276.04126 (|Δ| = 2.11 ppm). ***R*F**: 0.32 (heptane/AcOEt, 4:1). **Yield**: 20%.

1-Phenyl-4-[(trifluoromethyl)sulfanyl]-1*H*-1,2,3-triazole 5f

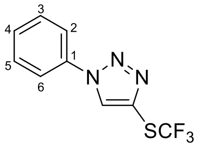

According to the general procedure, the reaction of phenyl azide **8f** (250 μL, 0.125 mmol, 0.5 M solution in ^{t}BuOMe) with CF_3S–C≡CH (**3**) at 30 °C for 48 h afforded triazole **5f** (22.4 mg, 0.09 mmol) as a brown solid. ^{1}**H NMR** [400 MHz, δ (ppm), $CDCl_3$]: 8.30 (s, 1 H, C*H*), 7.78–7.73 (m, 2 H, 2-C*H* + 6-C*H*), 7.61–7.55 (m, 2 H, 3-C*H* + 5-C*H*), 7.54–7.48 (m, 1 H, 4-C*H*). ^{13}**C NMR** [101 MHz, δ (ppm), $CDCl_3$]: 136.2 (1-*C*), 130.5 (*C*–S, indirect observation), 130.2 (3-*C* + 5-C), 129.8 (4-*C*), 128.5 (*C*H), 120.9 (2-*C* + 6-C). The carbon signal of SCF_3 was not observed. ^{19}**F NMR** [377 MHz, δ (ppm), $CDCl_3$]: −42.8. **FTIR** [$\tilde{\nu}$ (cm^{-1})]: 2989, 1141, 1120, 1041, 758.

HRMS [ESI (m/z)] calcd for $(C_9H_6F_3N_3S + H)^+$ = 246.03128, found 246.03124 (|Δ| = 0.15 ppm). ***R*F**: 0.36 (heptane/AcOEt, 4:1). **Yield**: 73%.

1-(3-Chlorophenyl)-4-[(trifluoromethyl)sulfanyl]-1*H*-1,2,3-triazole 5g

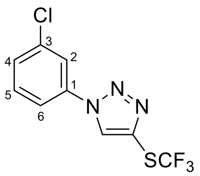

According to the general procedure, the reaction of 1-azido-3-chlorobenzene **8g** (200 μL, 0.10 mmol, 0.5 M solution in ^{t}BuOMe) with CF_3S–C≡CH (**3**) at 30 °C for 16 h afforded triazole **5g** (21.0 mg, 0.075 mmol) as a brown solid. **^{1}H NMR** [400 MHz, δ (ppm), $CDCl_3$]: 8.32 (s, 1 H, C*H*), 7.81 (td, *J* = 1.9, 0.6 Hz, 1 H, 2-C*H*), 7.67 (dt, *J* = 7.4, 2.0 Hz, 1 H, 6-C*H*), 7.53 (t, *J* = 7.7 Hz, 1 H, 5-C*H*), 7.48 (dt, *J* = 8.1, 1.8 Hz, 1 H, 4-C*H*). **^{13}C NMR** [101 MHz, δ (ppm), $CDCl_3$]: 137.2 (1-*C* or 3-*C*), 136.1 (1-*C* or 3-*C*), 131.2 (5-*C*), 131.0 (*C*–S), 129.9 (4-*C*), 128.43 (*C*H), 128.39 (q, *J* = 309.8 Hz, SCF_3), 121.1 (2-*C*), 118.8 (6-*C*). **^{19}F NMR** [377 MHz, δ (ppm), $CDCl_3$]: –42.7. **FTIR** [$\bar{\nu}$ (cm^{-1})]: 3124, 1597, 1155, 1118, 1040, 784. **HRMS** [ESI (m/z)] calcd for $(C_9H_5F_3N_3SCl + H)^+$ = 279.99231, found 279.99238 (|Δ| = 0.26 ppm). ***R*F**: 0.40 (heptane/AcOEt, 4:1). **Yield**: 75%.

1-(3-Methoxyphenyl)-4-[(trifluoromethyl)sulfanyl]-1*H*-1,2,3-triazole 5h[9]

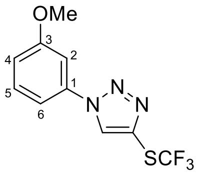

According to the general procedure, the reaction of 1-azido-3-methoxybenzene **8h** (19 mg, 0.13 mmol) with CF_3S–C≡CH (**3**) at 50 °C for 48 h afforded triazole **5h** (12.9 mg, 0.047 mmol) as a brown solid. **^{1}H NMR** [400 MHz, δ (ppm), $CDCl_3$]: 8.28 (s, 1 H, C*H*), 7.45 (t, *J* = 8.2 Hz, 1 H, 5-C*H*), 7.35 (t, *J* = 2.3 Hz, 1 H, 2-C*H*), 7.28 (ddd, *J* = 8.2, 2.1, 0.9 Hz, 1 H, 6-C*H*), 7.03 (ddd, *J* = 8.2, 2.6, 0.9 Hz, 1 H, 4-C*H*), 3.90 (s, 3 H, OC*H*3). **^{13}C NMR** [101 MHz, δ (ppm), $CDCl_3$]: 160.9 (3-*C*), 137.5 (1-*C*), 130.9 (5-*C*), 130.4 (*C*–S), 128.6 (*C*H), 128.5 (q, *J* = 309.6 Hz, SCF_3), 115.6 (4-*C*), 112.6 (6-*C*), 106.7 (2-*C*), 55.9 (O*C*H3). **^{19}F NMR** [377 MHz, δ (ppm), $CDCl_3$]: –42.8. **FTIR** [$\bar{\nu}$ (cm^{-1})]: 2932, 1612, 1146, 1110, 1032. **HRMS** [ESI (m/z)] calcd for $(C_{10}H_8F_3N_3OS + H)^+$ = 276.04184, found 276.04185 (|Δ| = 0.03 ppm). ***R*F**: 0.34 (heptane/AcOEt, 4:1). **Yield**: 36%.

3-{4-[(Trifluoromethyl)sulfanyl]-1*H*-1,2,3-triazol-1-yl}benzonitrile 5i

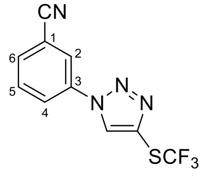

According to the general procedure, the reaction of 3-azidobenzonitrile **8i** (20 mg, 0.14 mmol) with CF_3S–C≡CH (**3**) at 50 °C for 48 h afforded triazole **5i** (15.1 mg, 0.056 mmol) as a brown solid. **^{1}H NMR** [400 MHz, δ (ppm), $CDCl_3$]: 8.35 (s, 1 H, C*H*), 8.10 (t, *J* = 1.9 Hz, 1 H, 2-C*H*), 8.05 (ddd, *J* = 8.1, 2.3, 1.2 Hz, 1 H, 4-C*H*), 7.81 (dt, *J* = 7.8, 1.3 Hz, 1 H, 6-C*H*), 7.73 (t, *J* = 7.9 Hz, 1 H, 5-C*H*). **^{13}C NMR** [101 MHz, δ (ppm), $CDCl_3$]: 137.0 (3-*C*),

133.1 (6-*C*), 131.36 (5-*C*), 131.34 (*C*–S), 128.2 (*C*H), 124.8 (4-*C*), 124.0 (2-*C*), 117.1 (*C*N), 114.7 (1-*C*). The carbon signal of SCF_3 was not observed. **^{19}F NMR** [377 MHz, δ (ppm), $CDCl_3$]: –42.5. **FTIR** [$\bar{\nu}$ (cm^{-1})]: 2930, 2235, 1109, 1032, 755. ***R*F**: 0.17 (heptane/AcOEt, 4:1). **Yield**: 40%.

1-(2-Methoxyphenyl)-4-[(trifluoromethyl)sulfanyl]-1*H*-1,2,3-triazole 5j[9]

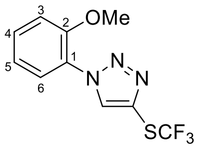

According to the general procedure, the reaction of 1-azido-2-methoxybenzene **8j** (19 mg, 0.13 mmol) with $CF_3S–C≡CH$ (**3**) at 50 °C for 48 h afforded triazole **5j** (11.0 mg, 0.04 mmol) as a brown solid. **^{1}H NMR** [400 MHz, δ (ppm), $CDCl_3$]: 8.48 (s, 1 H, C*H*), 7.86 (dd, *J* = 7.9, 1.7 Hz, 1 H, 6-C*H*), 7.47 (ddd, *J* = 8.3, 7.6, 1.7 Hz, 1 H, 4-C*H*), 7.17–7.10 (m, 2 H, 3-C*H* + 5-C*H*), 3.93 (s, 3 H, OC*H*3). **^{13}C NMR** [101 MHz, δ (ppm), $CDCl_3$]: 150.8 (2-*C*), 132.4 (*C*H), 130.8 (4-*C*), 128.8 (*C*–S), 128.4 (q, *J* = 309.7 Hz, SCF_3), 125.6 (1-*C*), 125.1 (6-*C*), 121.4 (5-*C*), 112.3 (3-*C*), 56.1 (OCH_3). **^{19}F NMR** [377 MHz, δ (ppm), $CDCl_3$]: –43.1. **FTIR** [$\bar{\nu}$ (cm^{-1})]: 2939, 1604, 1510, 1285, 1256, 1106, 1026, 754. **HRMS** [ESI (m/z)] calcd for $(C_{10}H_8F_3N_3OS + H)^+$ = 276.04184, found 276.04158 (|Δ| = 0.93 ppm). ***R*F**: 0.38 (heptane/AcOEt, 4:1). **Yield**: 31%.

2-{4-[(Trifluoromethyl)sulfanyl]-1*H*-1,2,3-triazol-1-yl}benzonitrile 5k

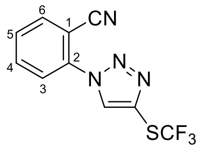

According to the general procedure, the reaction of 2-azidobenzonitrile **8k** (18 mg, 0.12 mmol) with $CF_3S–C≡CH$ (**3**) at 50 °C for 48 h afforded triazole **5k** (9.7 mg, 0.036 mmol) as a brown solid. **^{1}H NMR** [400 MHz, δ (ppm), $CDCl_3$]: 8.59 (s, 1 H, C*H*), 7.95 (dd, *J* = 8.2, 1.2 Hz, 1 H, 3-C*H*), 7.91 (dd, *J* = 7.8, 1.5 Hz, 1 H, 6-C*H*), 7.87 (td, *J* = 7.8, 1.5 Hz, 1 H, 4-C*H*), 7.68 (td, *J* = 7.7, 1.3 Hz, 1 H, 5-C*H*). **^{13}C NMR** [101 MHz, δ (ppm), $CDCl_3$]: 137.8 (2-*C*), 134.8 (4-*C*), 134.6 (6-*C*), 131.0 (*C*–S, indirect observation), 130.8 (*C*H), 130.5 (5-*C*), 128.4 (q, *J* = 309.9 Hz, SCF_3), 125.7 (3-*C*), 115.3 (*C*N), 106.9 (1-*C*). **^{19}F NMR** [377 MHz, δ (ppm), $CDCl_3$]: –42.6. **FTIR** [$\bar{\nu}$ (cm^{-1})]: 2923, 2853, 1520, 1349, 1146, 1103, 1034, 853. **HRMS** [ESI (m/z)] calcd for $(C_{10}H_5F_3N_4S + H)^+$ = 271.02653, found 271.02598 (|Δ| = 2.00 ppm). ***R*F**: 0.14 (heptane/AcOEt, 4:1). **Yield**: 30%.

1-(2,6-Difluorophenyl)-4-[(trifluoromethyl)sulfanyl]-1*H*-1,2,3-triazole 5l

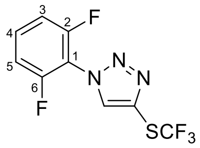

According to the general procedure, the reaction of 2-azido-1,3-difluorobenzene **8l** (21 mg, 0.14 mmol) with CF_3S–C≡CH (**3**) at 50 °C for 48 h afforded triazole **5l** (23.9 mg, 0.085 mmol) as a brown solid. **^{1}H NMR** [400 MHz, δ (ppm), $CDCl_3$]: 8.18 (s, 1 H, C*H*), 7.55 (tt, *J* = 8.6, 6.0 Hz, 1 H, 4-C*H*), 7.22–7.14 (m, 2 H, 3-C*H* + 5-C*H*). **^{13}C NMR** [101 MHz, δ (ppm), $CDCl_3$]: 156.8 (dd, *J* = 257.5, 2.7 Hz, 2-*C* + 6-*C*), 133.0 (CH), 132.3 (t, *J* = 9.7 Hz, 4-C*H*), 130.0 (*C*–S), 128.37 (q, *J* = 309.7 Hz, SCF_3), 113.0–112.7 (m, 3-*C* + 5-*C*). The signal of 1-*C* was not observed. **^{19}F NMR** [377 MHz, δ (ppm), $CDCl_3$]: –42.82 (SC*F*$_3$). **FTIR** [$\bar{\nu}$ (cm^{-1})]: 2919, 1480, 1111, 1032, 1014, 788. **HRMS** [ESI (m/z)] calcd for $(C_9H_4F_5N_3S + H)^+$ = 282.01243, found 282.01226 (|Δ| = 0.63 ppm). ***R*$_F$**: 0.21 (heptane/AcOEt, 4:1). **Yield**: 61%.

4-{4-[(Trifluoromethyl)sulfanyl]-1*H*-1,2,3-triazol-1-yl}pyridine 5m

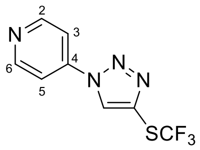

According to the general procedure, the reaction of 4-azidopyridine **8m** (19 mg, 0.16 mmol) with CF_3S–C≡CH (**3**) at 70 °C for 16 h afforded triazole **5m** (25.6 mg, 0.104 mmol) as a brown solid. **^{1}H NMR** [400 MHz, δ (ppm), $CDCl_3$]: 9.10–8.60 (m, 2 H, 2-C*H* + 6-C*H*), 8.44 (s, 1 H, C*H*), 7.79–7.72 (m, 2 H, 3-C*H* + 5-C*H*). **^{13}C NMR** [101 MHz, δ (ppm), $CDCl_3$]: 150.9 (2-*C* + 6-*C*), 141.4 (4-*C*), 130.7 (*C*–S), 127.2 (q, *J* = 310.0 Hz, SCF_3), 126.7 (CH), 112.8 (3-*C* + 5-*C*). **^{19}F NMR** [377 MHz, δ (ppm), $CDCl_3$]: –42.5. **FTIR** [$\bar{\nu}$ (cm^{-1})]: 3113, 1587, 1510, 1150, 1111, 1037, 845, 706. ***R*$_F$**: 0.12 (heptane/AcOEt, 4:1). **Yield**: 65%.

1-Benzyl-4-[(trifluoromethyl)sulfanyl]-1*H*-1,2,3-triazole 5n

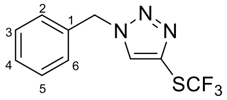

According to the general procedure, the reaction of (azidomethyl)benzene **8n** (22 mg, 0.17 mmol) with CF_3S–C≡CH (**3**) at 50 °C for 16 h afforded triazole **5n** (27.2 mg, 0.105 mmol) as a brown solid. **^{1}H NMR** [400 MHz, δ (ppm), $CDCl_3$]: 7.76 (s, 1 H, C*H*), 7.44–7.37 (m, 3 H, 3-C*H* + 4-C*H* + 5-C*H*), 7.31–7.27 (m, 2 H, 2-C*H* + 6-C*H*), 5.58 (s, 2 H, NC*H*$_2$). **^{13}C NMR** [101 MHz, δ (ppm), $CDCl_3$]: 133.7 (1-*C*), 130.3 (*C*–S), 130.2 (CH), 129.5 (3-*C* + 5-*C*), 129.4 (4-*C*), 128.42 (q, *J* = 309.5 Hz, SCF_3), 128.37 (2-*C* + 6-*C*), 54.9 (NCH_2). **^{19}F NMR** [377 MHz, δ (ppm), $CDCl_3$]: –43.0. **FTIR** [$\bar{\nu}$ (cm^{-1})]: 2362, 1984, 1143, 1129, 1044, 716. **HRMS** [ESI (m/z)] calcd for $(C_{10}H_8F_3N_3S + H)^+$ = 260.04693, found 260.04899 (|Δ| = 2.06 mmu). ***R*$_F$**: 0.28 (heptane/AcOEt, 4:1). **Yield**: 62%.

1-(Adamantan-1-yl)-4-[(trifluoromethyl)sulfanyl]-1*H*-1,2,3-triazole 5o

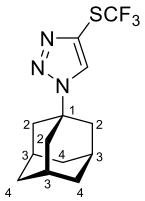

According to the general procedure, the reaction of 1-azidoadamantane **8o** (22 mg, 0.12 mmol) with $CF_3S–C≡CH$ (**3**) at 50 °C for 16 h afforded triazole **5o** (26.2 mg, 0.09 mmol) as a pale solid. **^{1}H NMR** [400 MHz, δ (ppm), $CDCl_3$]: 7.90 (s, 1 H, C*H*), 2.32–2.23 (m, 9 H, adamantyl), 1.88–1.75 (m, 6 H, adamantyl). **^{13}C NMR** [101 MHz, δ (ppm), $CDCl_3$]: 128.4 (q, *J* = 309.0 Hz, SCF_3), 128.2 (*C*–S), 126.9 (CH), 60.9 (1-C), 42.8 (2-C), 35.8 (4-C), 29.4 (3-C). **^{19}F NMR** [377 MHz, δ (ppm), $CDCl_3$]: –43.2. **FTIR** [$\bar{\nu}$ (cm^{-1})]: 2915, 1140, 1109, 1019. **HRMS** [ESI (m/z)] calcd for $(C_{13}H_{16}F_3N_3S + H)^+$ = 304.10953, found 304.11024 (|Δ| = 0.71 mmu). ***R*$_F$**: 0.48 (heptane/AcOEt, 4:1). **Yield**: 70%.

References and Notes

[1] H. C. Bertrand, M. Schaap, L. Baird, N. D. Georgakopoulos, A. Fowkes, C. Thiollier, H. Kachi, A. T. Dinkova-Kostova and G. Wells, *J. Med. Chem.,* 2015, **58**, 7186; S. R. Lanke and B. M. Bhanage, *Synth. Commun.,* 2014, **44**, 399; S. W. Kwok, J. R. Fotsing, R. J. Fraser, V. O. Rodionov and V. V. Fokin, *Org. Lett.,* 2010, **12**, 4217.

[2] H. K. Akula and M. K. Lakshman, *J. Org. Chem.,* 2012, **77**, 8896.

[3] A. V. Budruev, D. Y. Dzhons, V. I. Faerman, G. K. Fukin and A. S. Shavyrin, *Chem. Heterocycl. Compd.,* 2016, **52**, 694.

[4] J. Parello, V. Filimonov, K. Kutonova, M. Trusova and P. Postnikov, *Synthesis*, 2013, **45**, 2706.

[5] L. M. Jin, X. Xu, H. Lu, X. Cui, L. Wojtas and X. P. Zhang, *Angew. Chem. Int. Ed.,* 2013, **52**, 5309.

[6] G. Colombano, C. Travelli, U. Galli, A. Caldarelli, M. G. Chini, P. L. Canonico, G. Sorba, G. Bifulco, G. C. Tron and A. A. Genazzani, *J. Med. Chem.,* 2010, **53**, 616.

[7] A. Riesco-Domínguez, J. van de Wiel, T. A. Hamlin, B. van Beek, S. D. Lindell, D. Blanco-Ania, F. M. Bickelhaupt and F. P. J. T. Rutjes, *J. Org. Chem.,* 2018, **83**, 1779.

[8] Compound **5d** (R = Me) could not be isolated in pure form after column chromatography.

[9] The ratios between the corresponding 1,4- and 1,5-regioisomers were not determined from the crude mixtures but from the corresponding purified fractions.

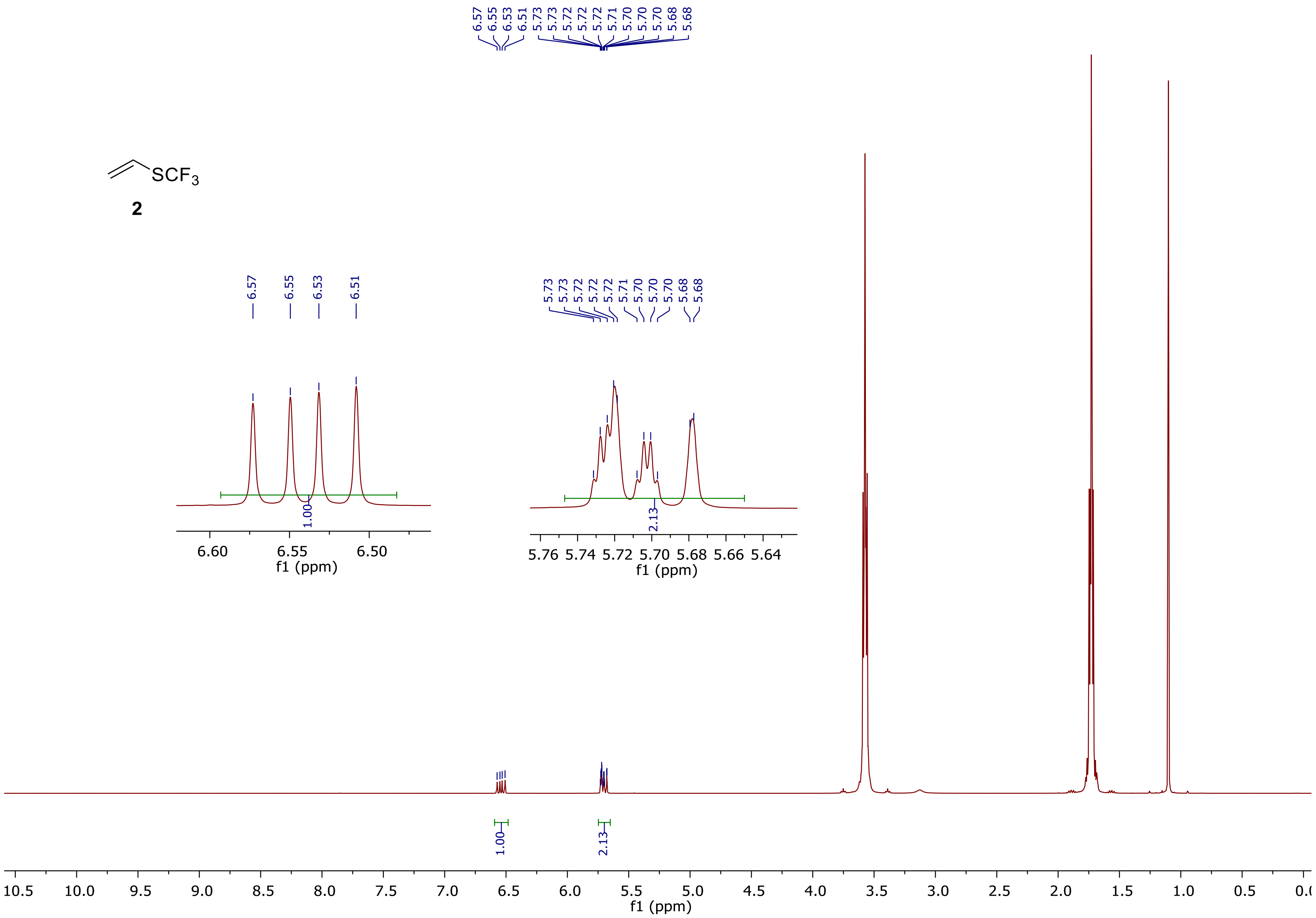

SCF3
2
6.57 6.55 6.53 6.51 5.73 5.73 5.72 5.72 5.72 5.71 5.70 5.70 5.70 5.68 5.68
1.00
2.13
6.60 6.55 6.50
f1 (ppm)
5.76 5.74 5.72 5.70 5.68 5.66 5.64
f1 (ppm)
10.5 10.0 9.5 9.0 8.5 8.0 7.5 7.0 6.5 6.0 5.5 5.0 4.5 4.0 3.5 3.0 2.5 2.0 1.5 1.0 0.5
f1 (ppm)

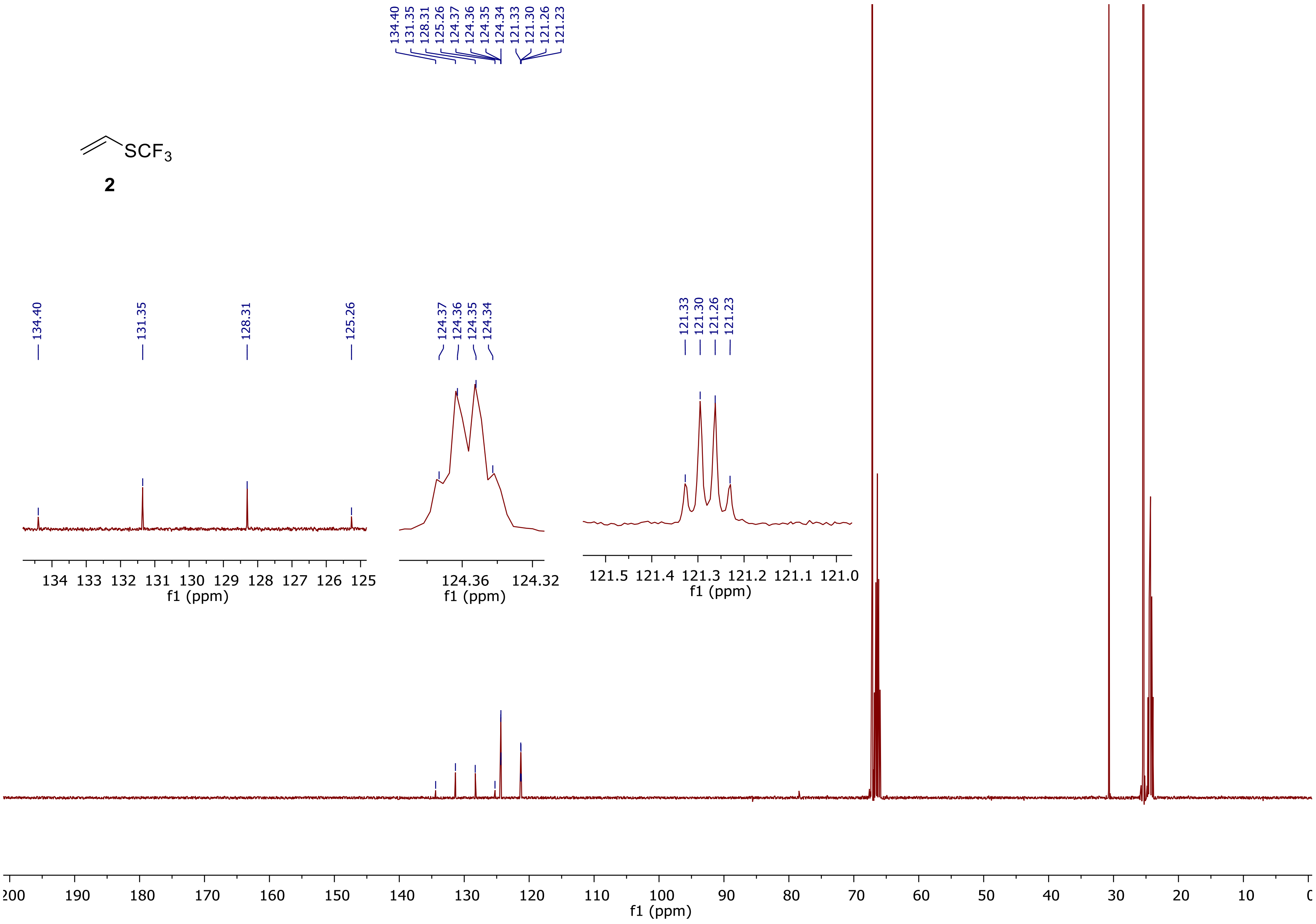
SCF3
2
134.40
131.35
128.31
125.26
124.37
124.36
124.35
124.34
121.33
121.30
121.26
121.23
f1 (ppm)

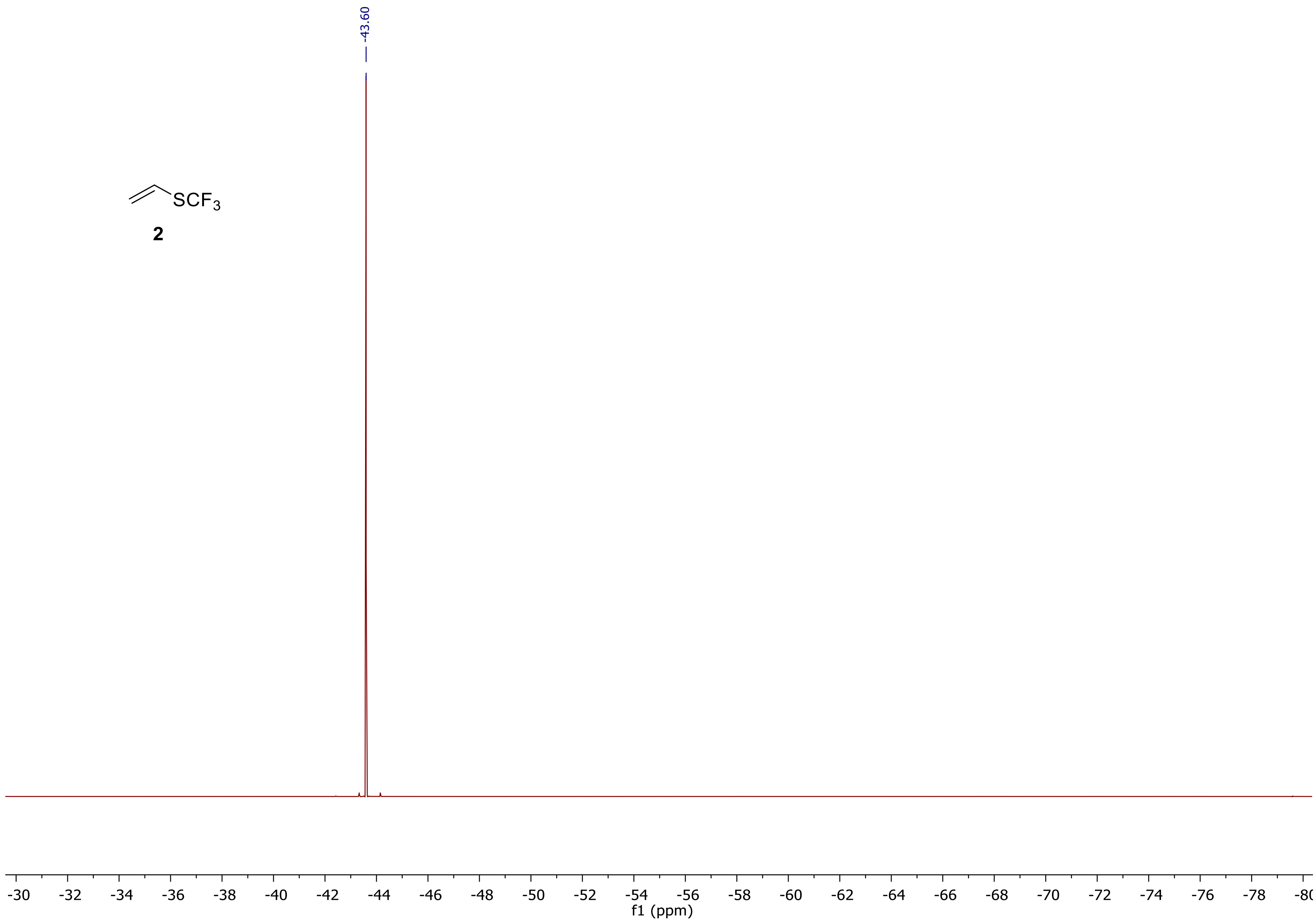

S2. ^{1}H, ^{13}C and ^{19}F NMR of Compound **2**

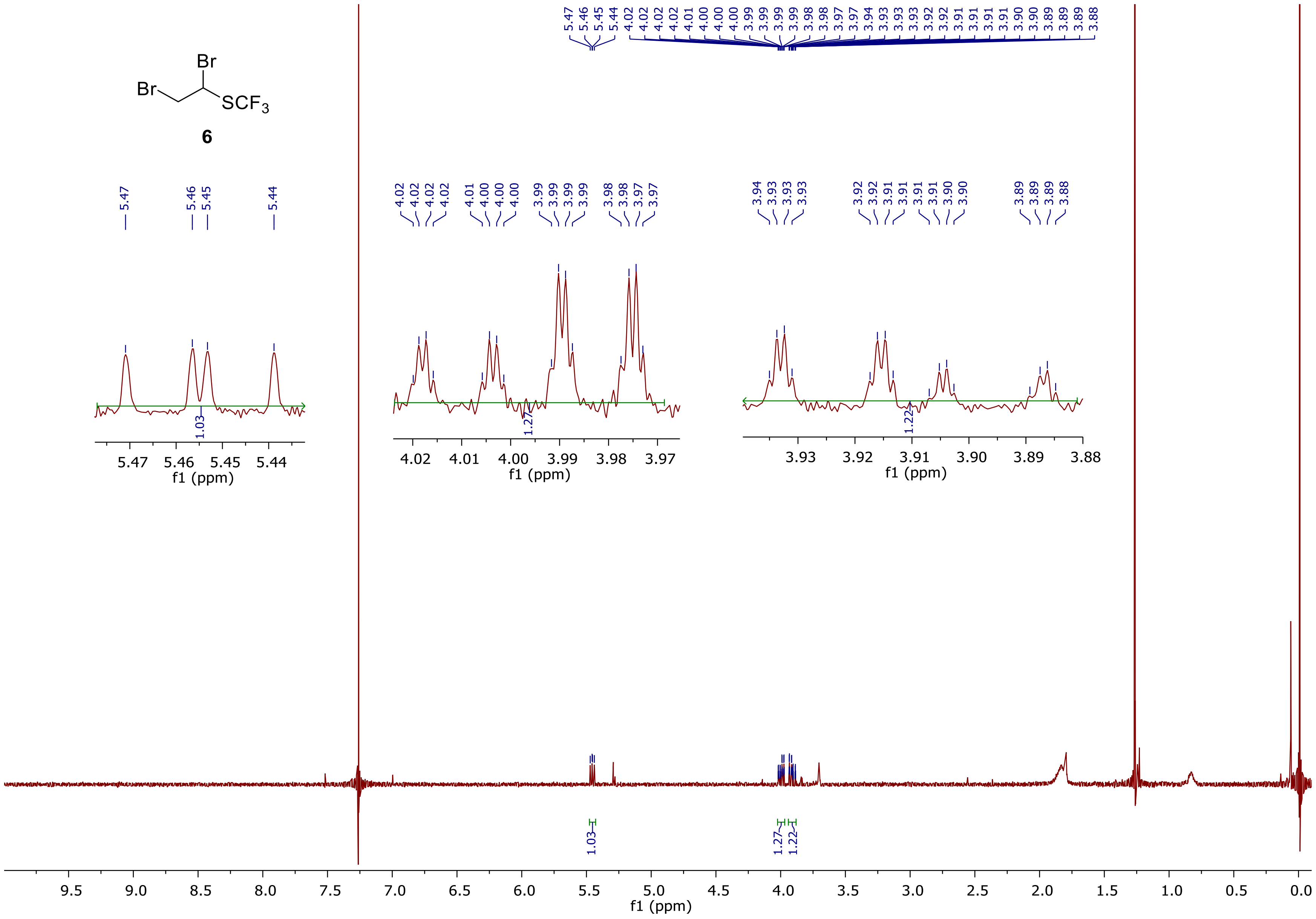

Br
Br
SCF3
6
f1 (ppm)

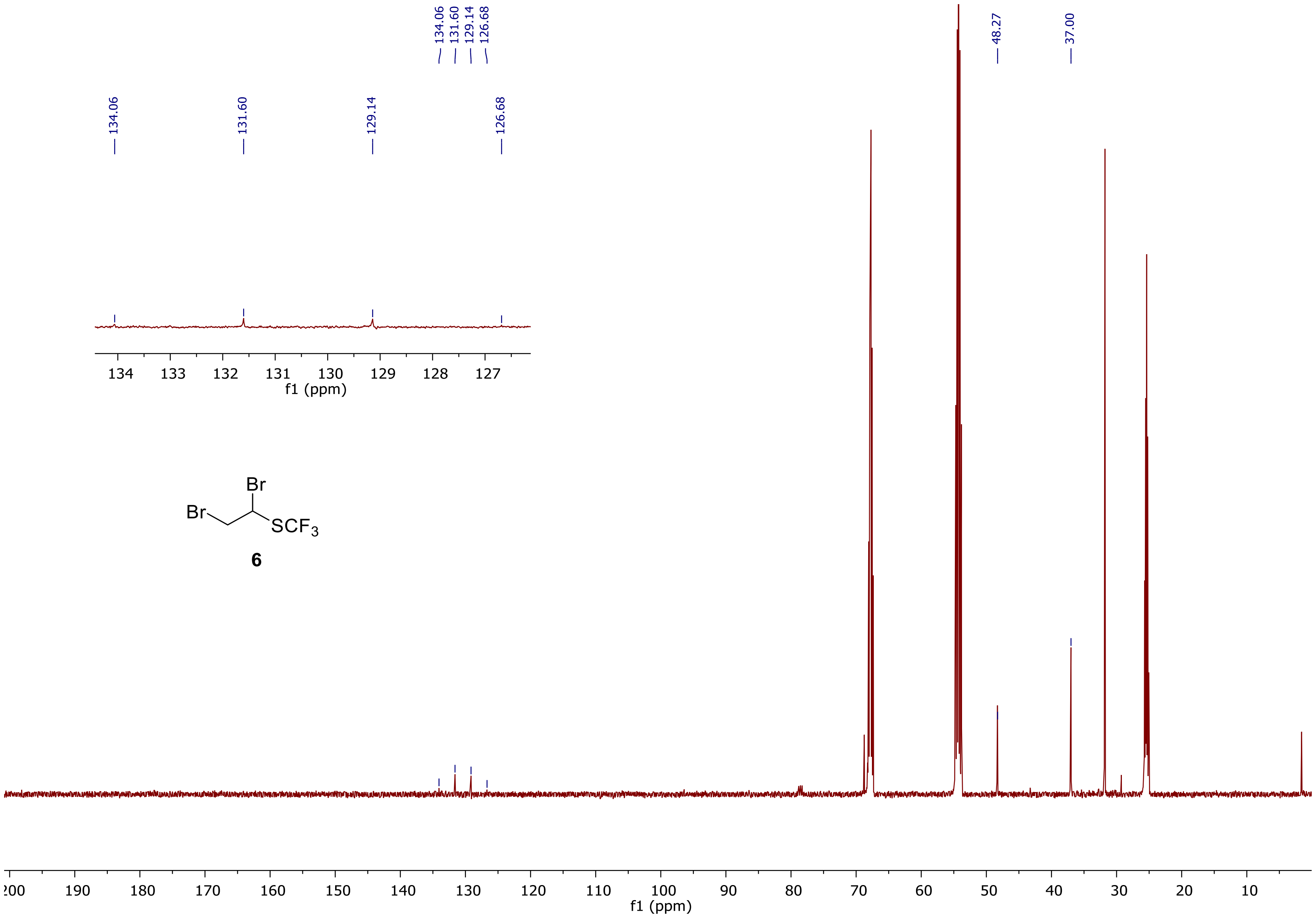
134.06
131.60
129.14
126.68
48.27
37.00
134 133 132 131 130 129 128 127
f1 (ppm)
Br
Br
SCF3
6
200 190 180 170 160 150 140 130 120 110 100 90 80 70 60 50 40 30 20 10
f1 (ppm)

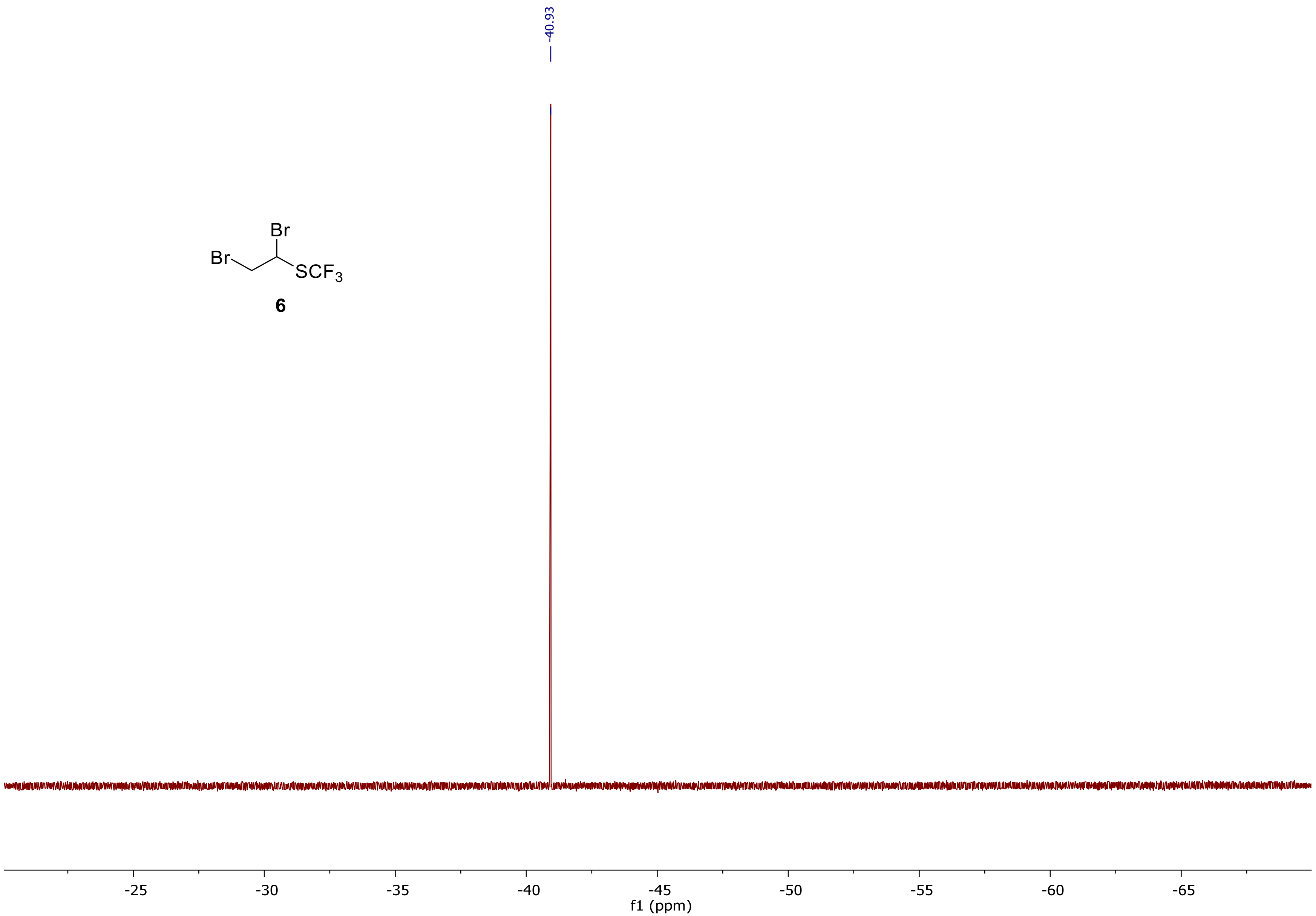

S3. ^{1}H, ^{13}C and ^{19}F NMR of Compound **2**

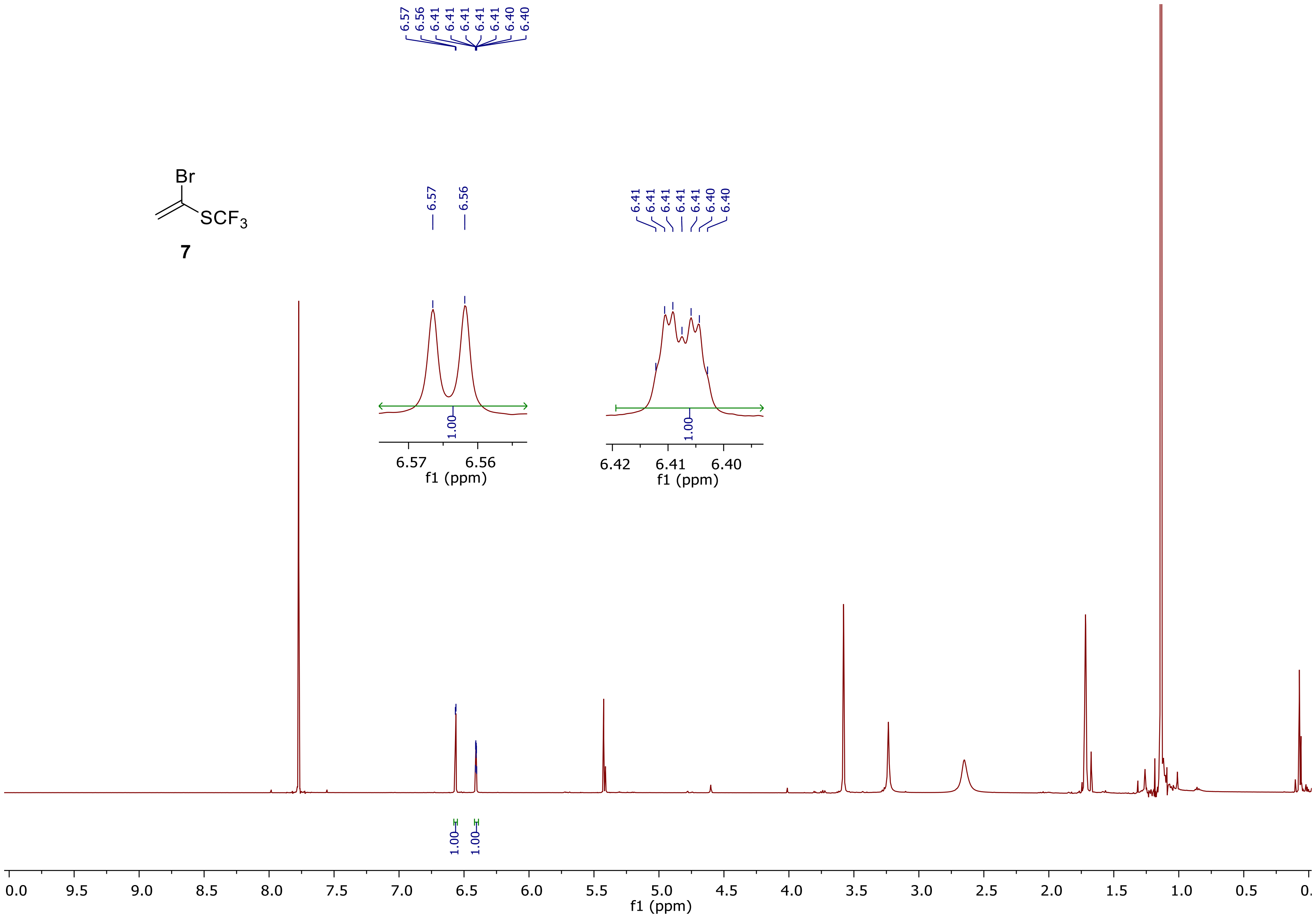
Br
SCF3
7
6.57
6.56
6.41
6.41
6.41
6.41
6.41
6.40
6.40
1.00
1.00
6.57 6.56
f1 (ppm)
6.42 6.41 6.40
f1 (ppm)
0.0 9.5 9.0 8.5 8.0 7.5 7.0 6.5 6.0 5.5 5.0 4.5 4.0 3.5 3.0 2.5 2.0 1.5 1.0 0.5 0.
f1 (ppm)

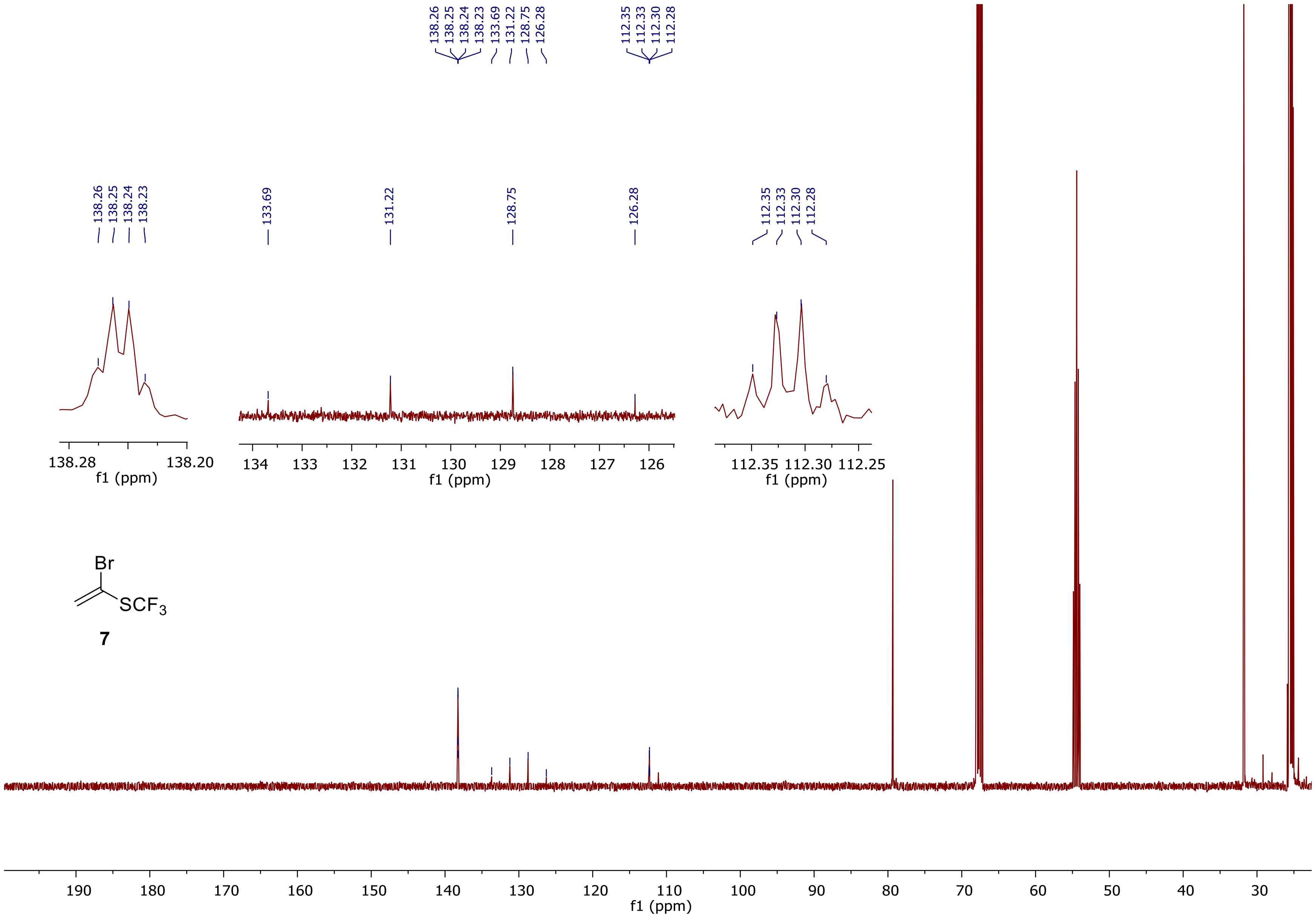
138.26
138.25
138.24
138.23
133.69
131.22
128.75
126.28
112.35
112.33
112.30
112.28
138.28
138.20
f1 (ppm)
134 133 132 131 130 129 128 127 126
f1 (ppm)
112.35 112.30 112.25
f1 (ppm)
Br
SCF3
7
190 180 170 160 150 140 130 120 110 100 90 80 70 60 50 40 30
f1 (ppm)

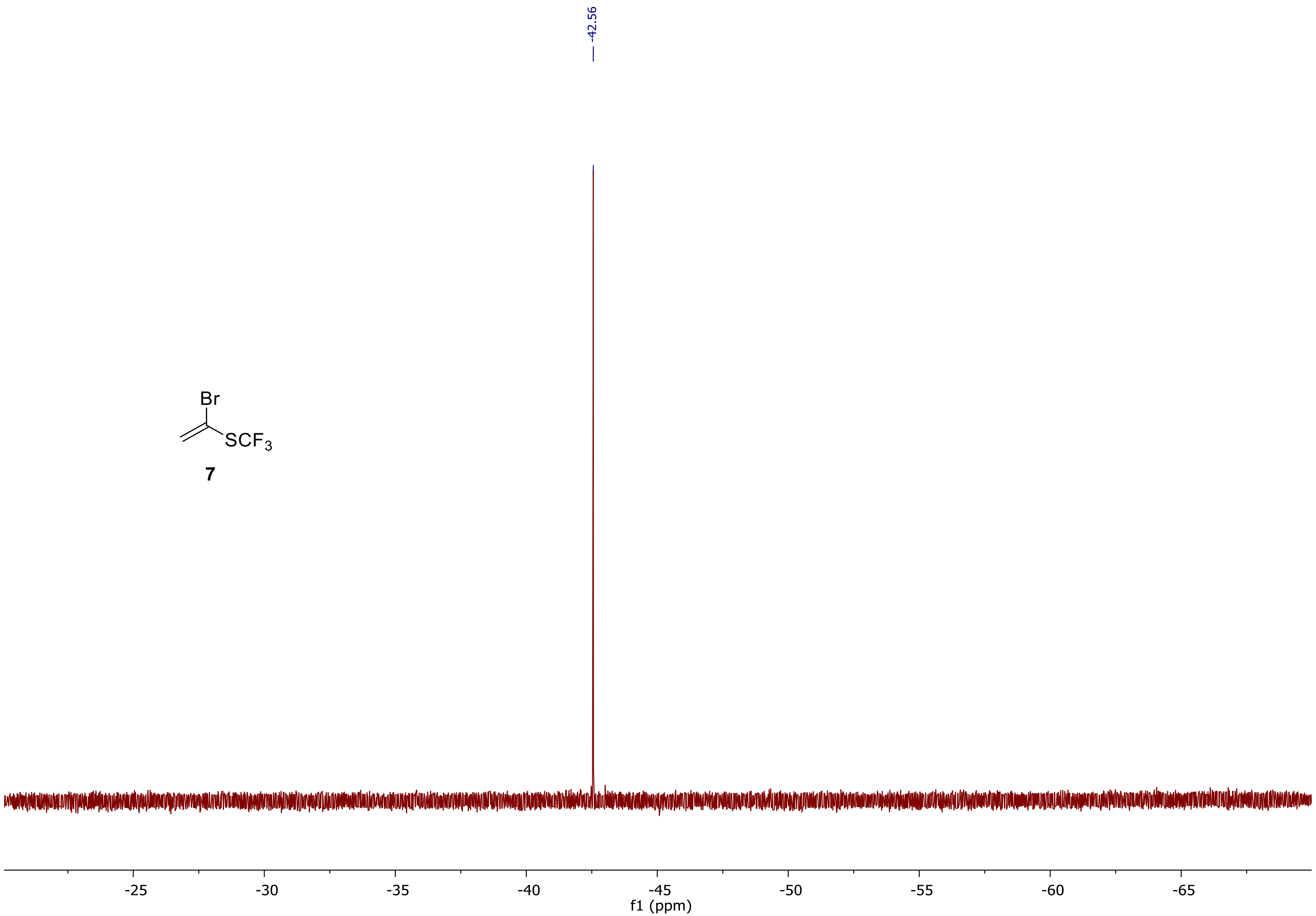

S4. ^{1}H, ^{13}C and ^{19}F NMR of Compound **7**

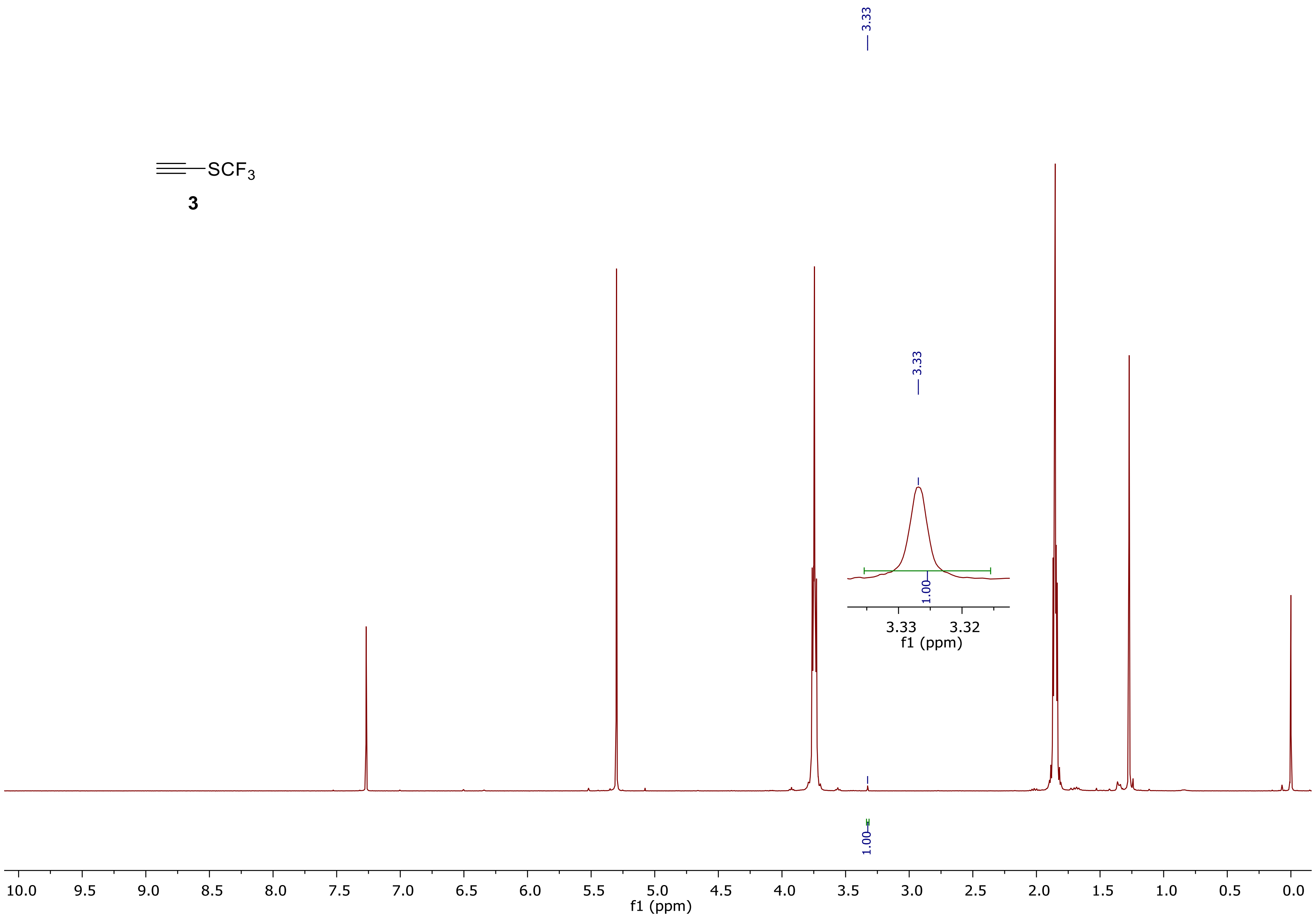

SCF3
3
3.33
3.33
1.00
3.33
3.32
f1 (ppm)
1.00
10.0
9.5
9.0
8.5
8.0
7.5
7.0
6.5
6.0
5.5
5.0
4.5
4.0
3.5
3.0
2.5
2.0
1.5
1.0
0.5
0.0
f1 (ppm)

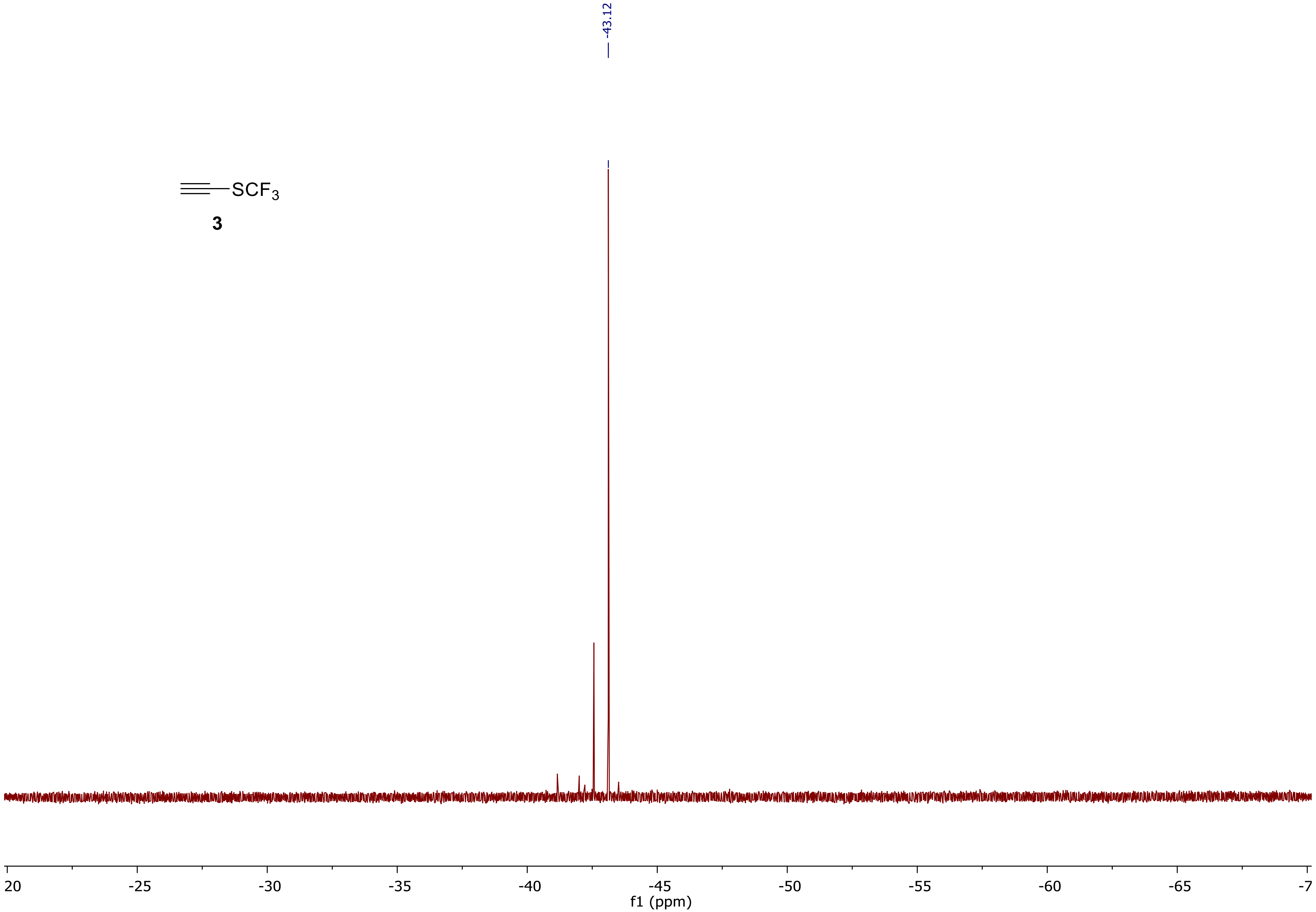

S5. ^{1}H and ^{19}F NMR of Compound **3**

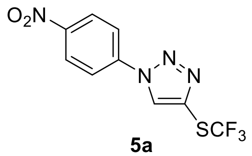
O2N
N
N
N
SCF3

5a

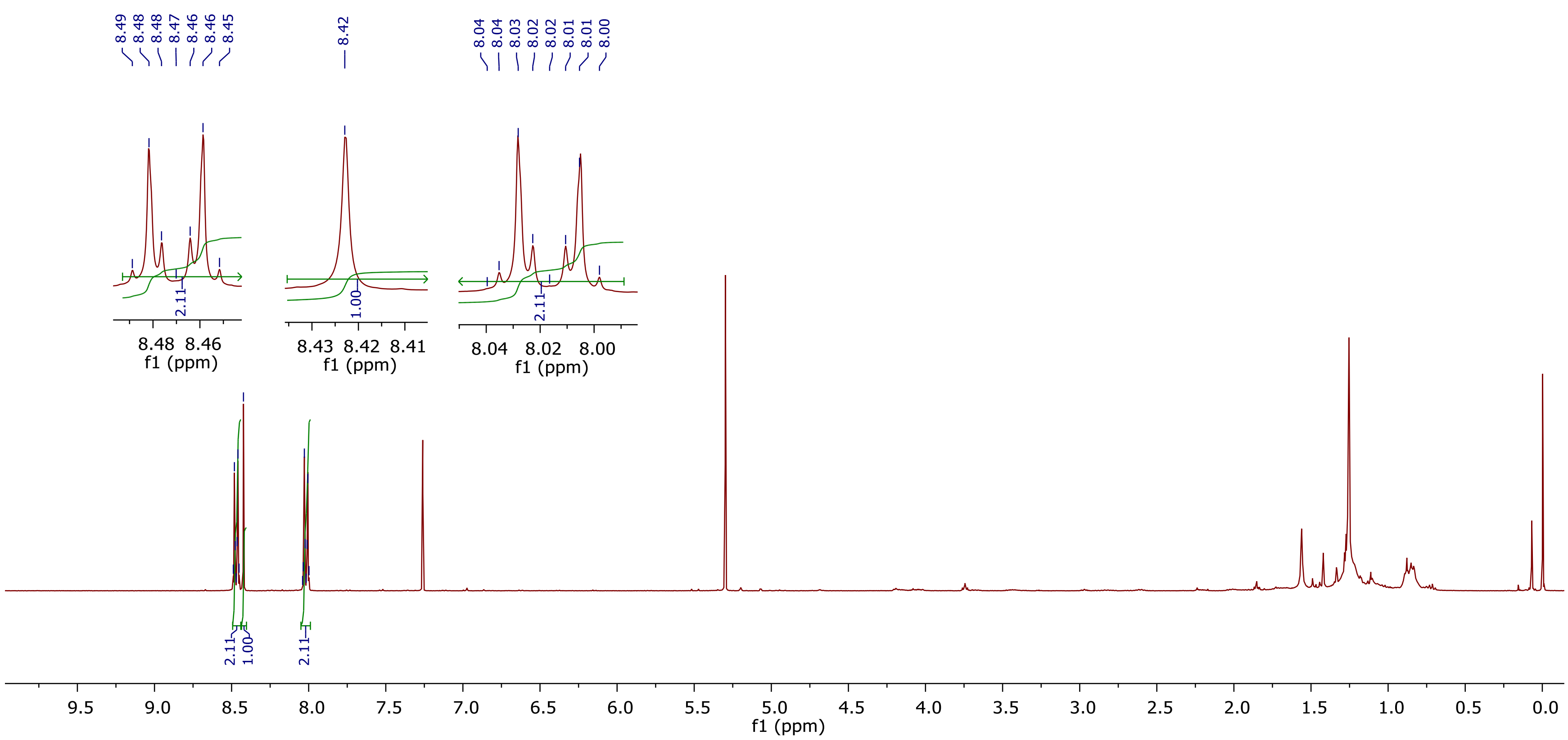
8.49
8.48
8.48
8.47
8.46
8.46
8.45
8.42
8.04
8.04
8.03
8.02
8.02
8.01
8.01
8.00
2.11
1.00
2.11
f1 (ppm)

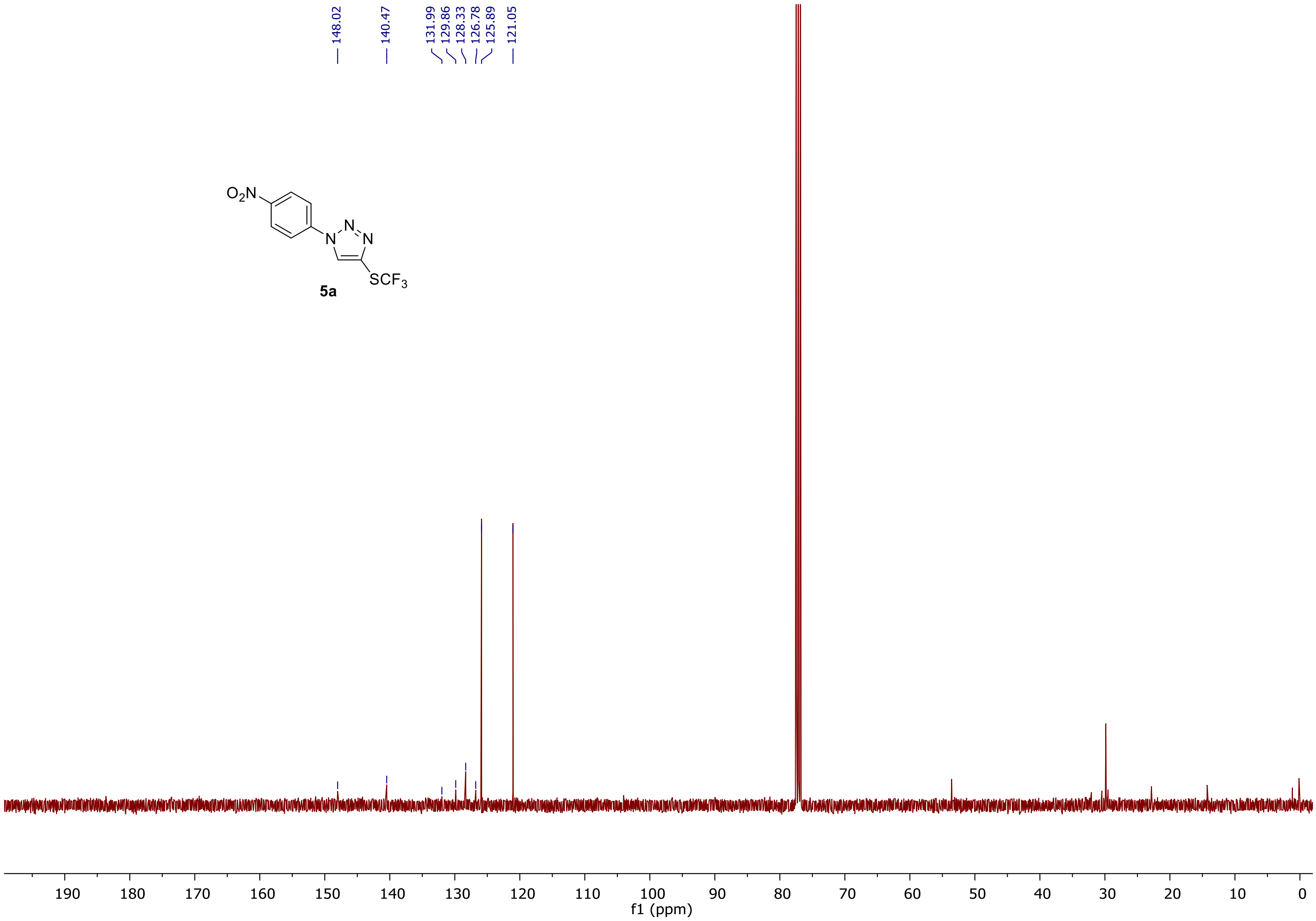
148.02
140.47
131.99
129.86
128.33
126.78
125.89
121.05
O2N
N
N
N
SCF3
5a
190 180 170 160 150 140 130 120 110 100 90 80 70 60 50 40 30 20 10 0
f1 (ppm)

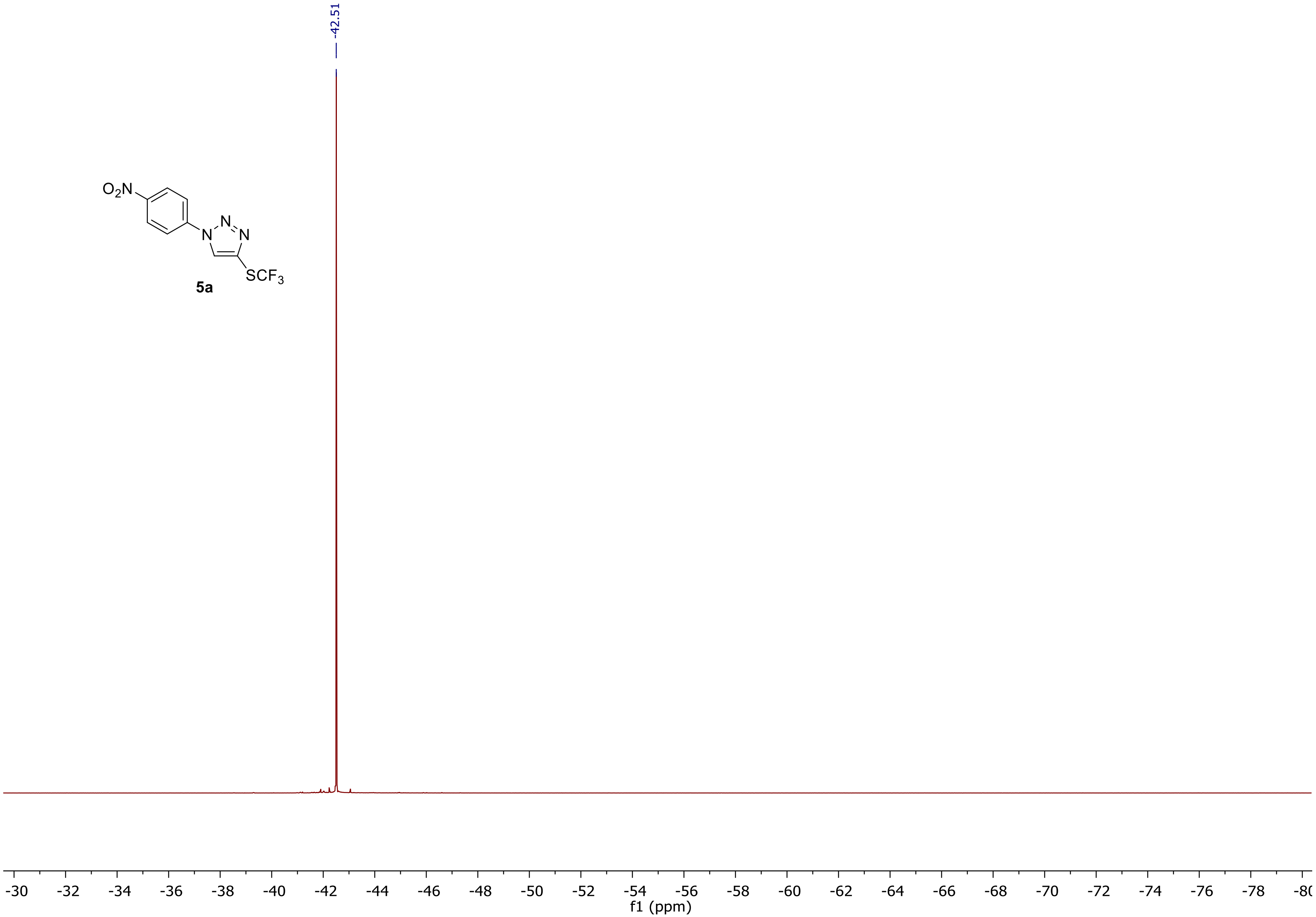

S6. ^{1}H, ^{13}C and ^{19}F NMR of Compound **5a**

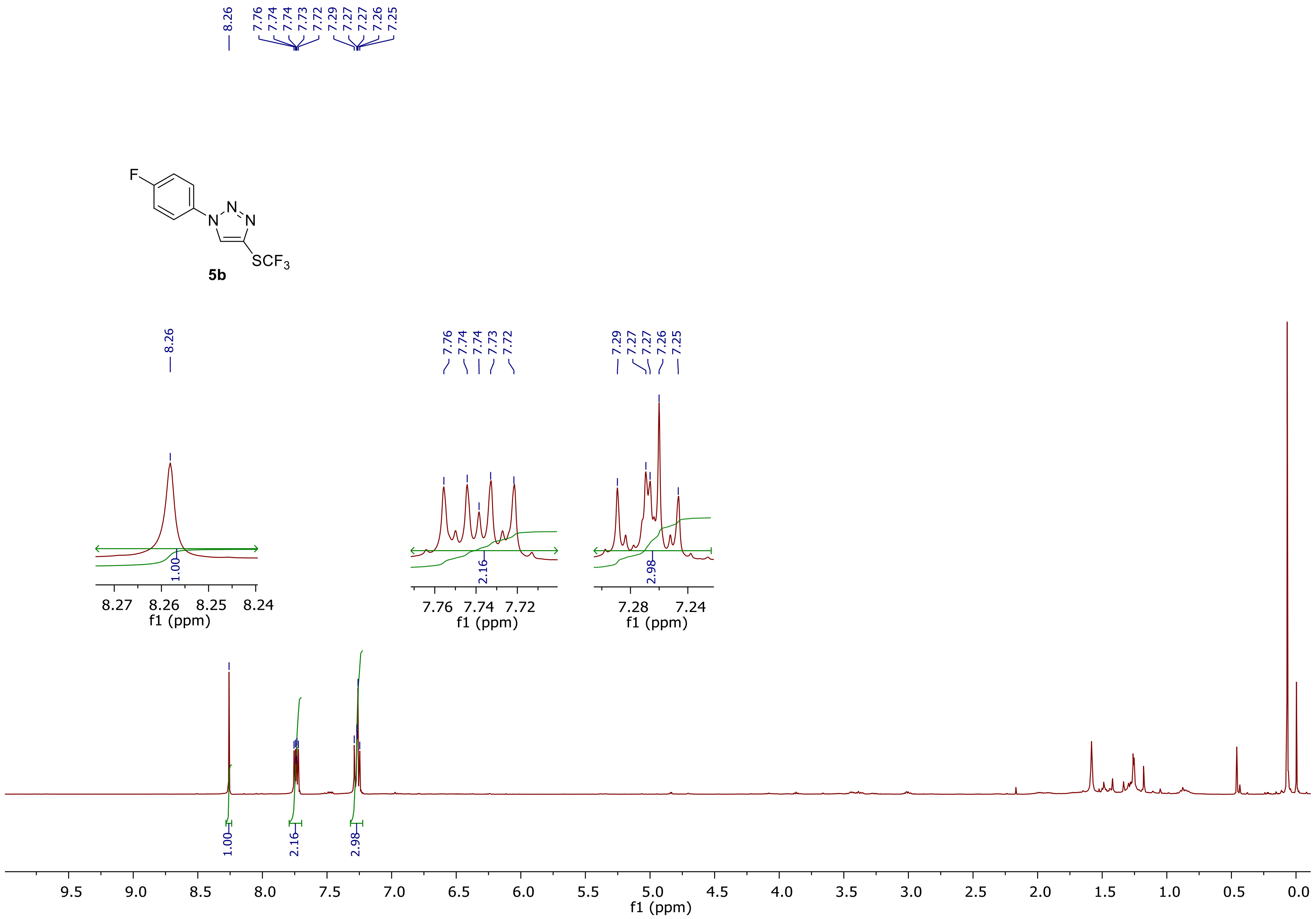
8.26
7.76
7.74
7.74
7.73
7.72
7.29
7.27
7.27
7.26
7.25
F
N
N
N
SCF3
5b
1.00
2.16
2.98
f1 (ppm)

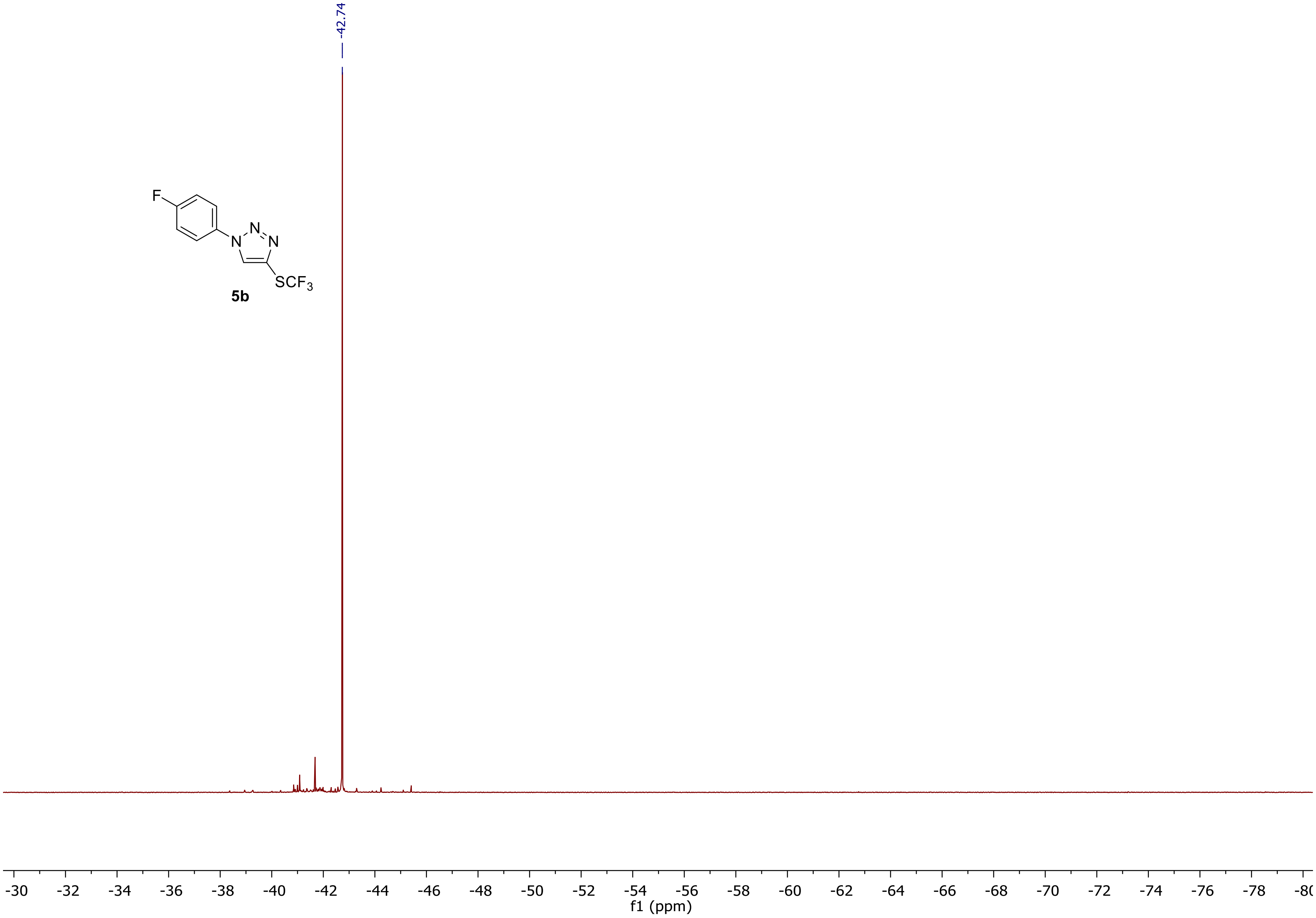
-42.74
F
N
N
N
SCF$_3$
5b
-30 -32 -34 -36 -38 -40 -42 -44 -46 -48 -50 -52 -54 -56 -58 -60 -62 -64 -66 -68 -70 -72 -74 -76 -78 -80
f1 (ppm)

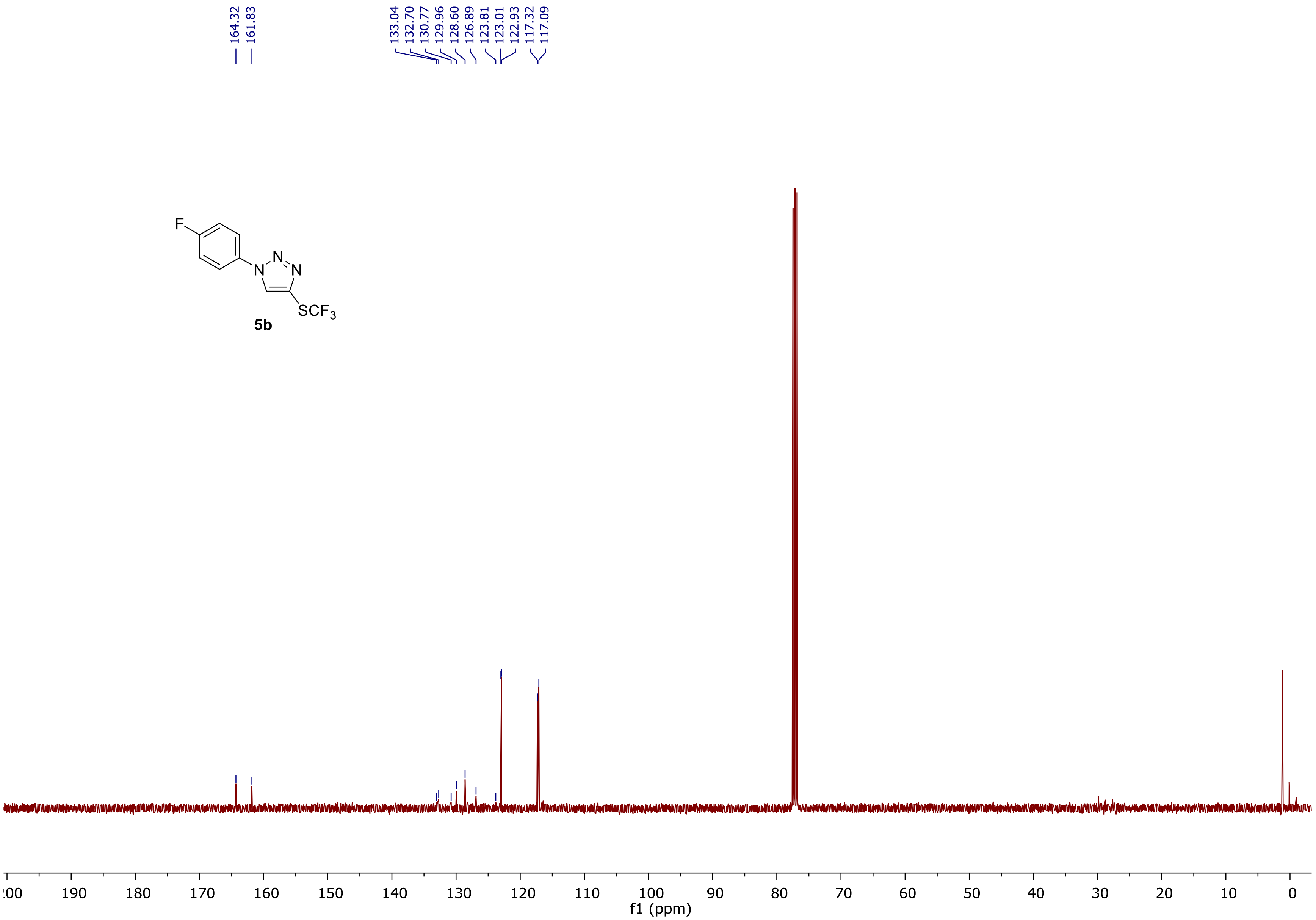

S7. ^{1}H, ^{13}C and ^{19}F NMR of Compound **5b**

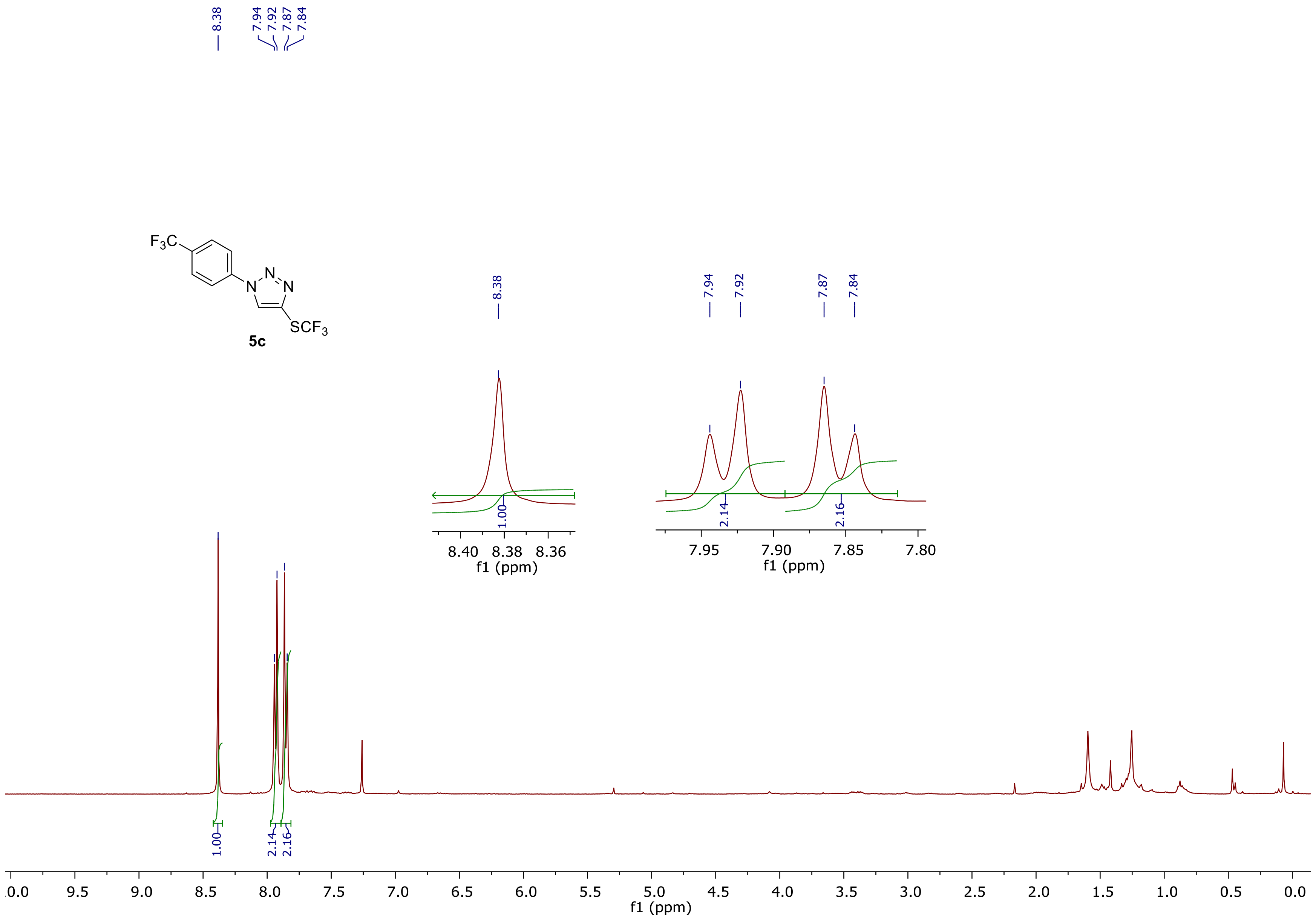
8.38
7.94
7.92
7.87
7.84
F3C
N
N
N
SCF3
5c
1.00
2.14
2.16
8.40
8.38
8.36
f1 (ppm)
7.95
7.90
7.85
7.80
f1 (ppm)
10.0
9.5
9.0
8.5
8.0
7.5
7.0
6.5
6.0
5.5
5.0
4.5
4.0
3.5
3.0
2.5
2.0
1.5
1.0
0.5
0.0
f1 (ppm)

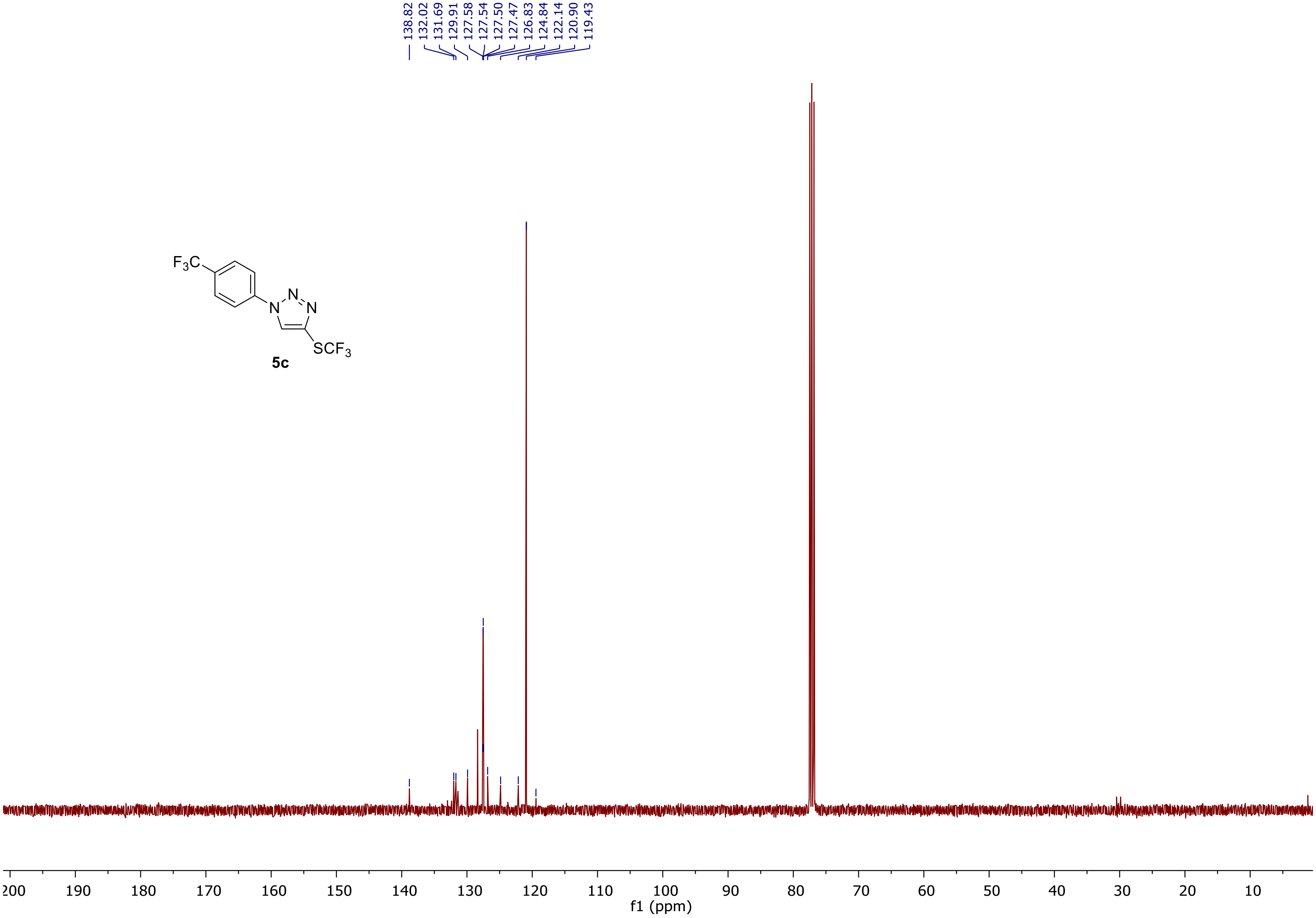
F3C
N
N
N
SCF3
5c
138.82
132.02
131.69
129.91
127.58
127.54
127.50
127.47
126.83
124.84
122.14
120.90
119.43
200
190
180
170
160
150
140
130
120
110
100
90
80
70
60
50
40
30
20
10
f1 (ppm)

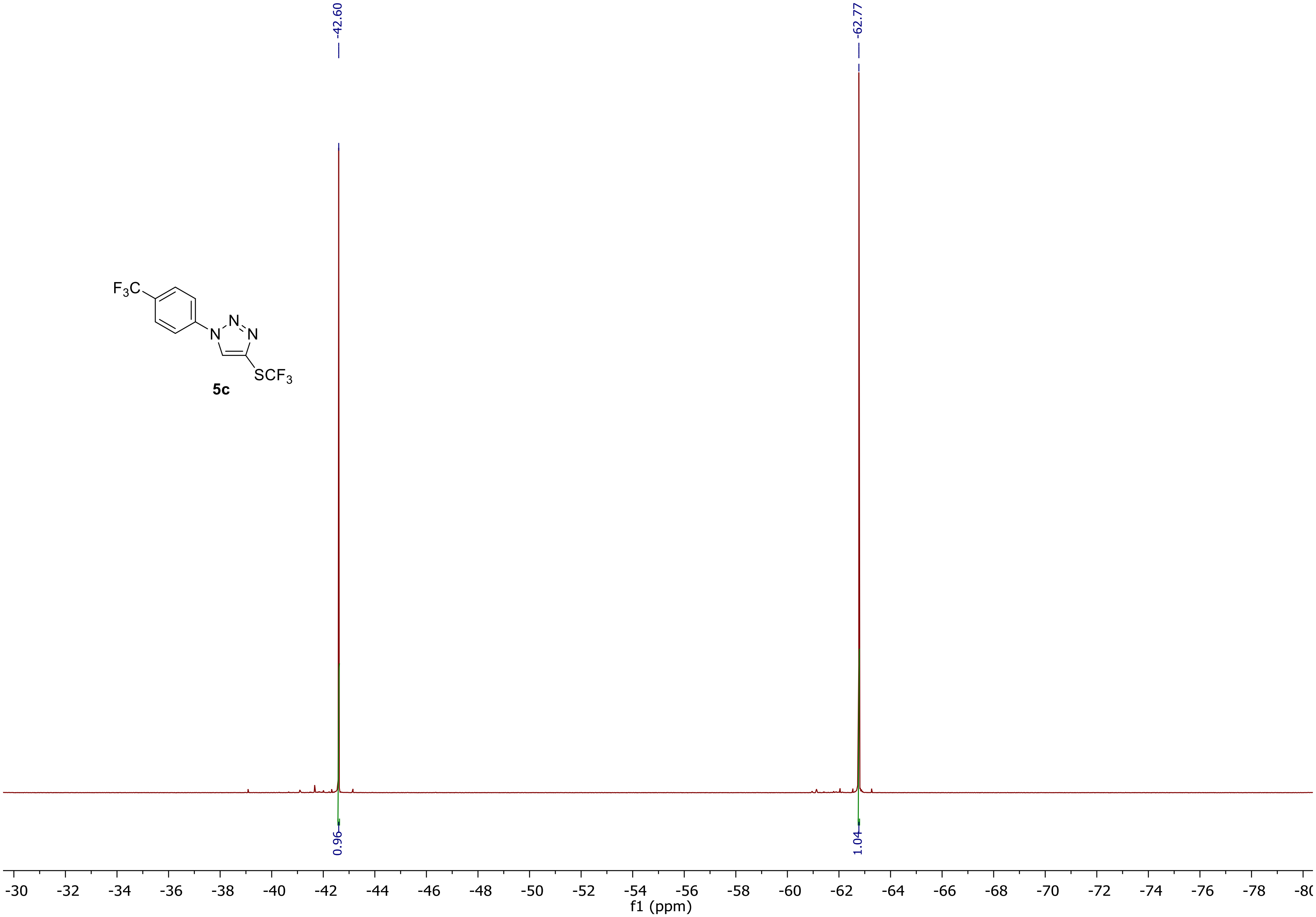

S8. ¹H, ¹³C and ¹⁹F NMR of Compound **5c**

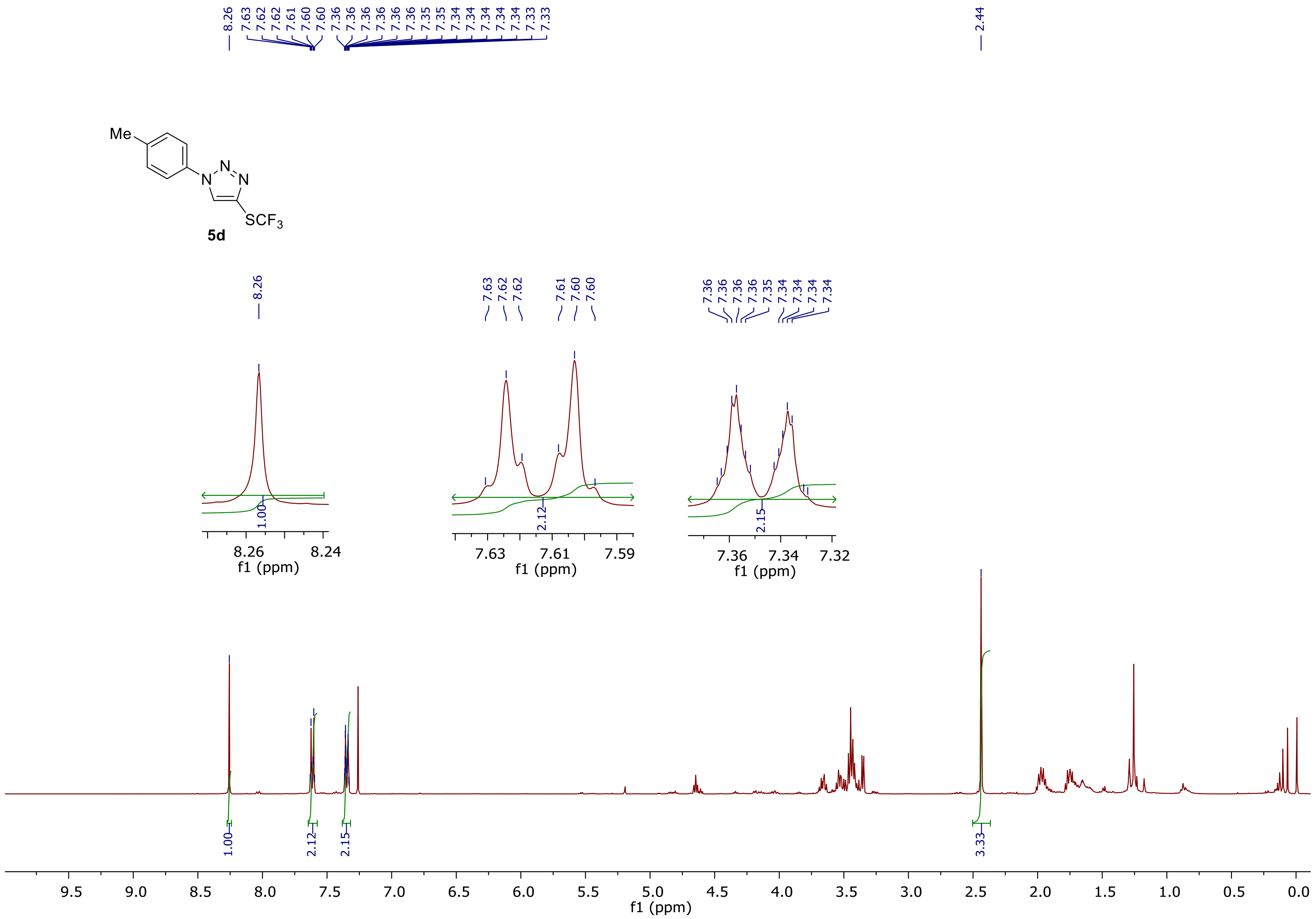
Me
N
N
N
SCF3
5d
f1 (ppm)

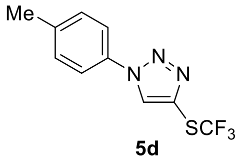
Me
N
N
N
SCF3

5d

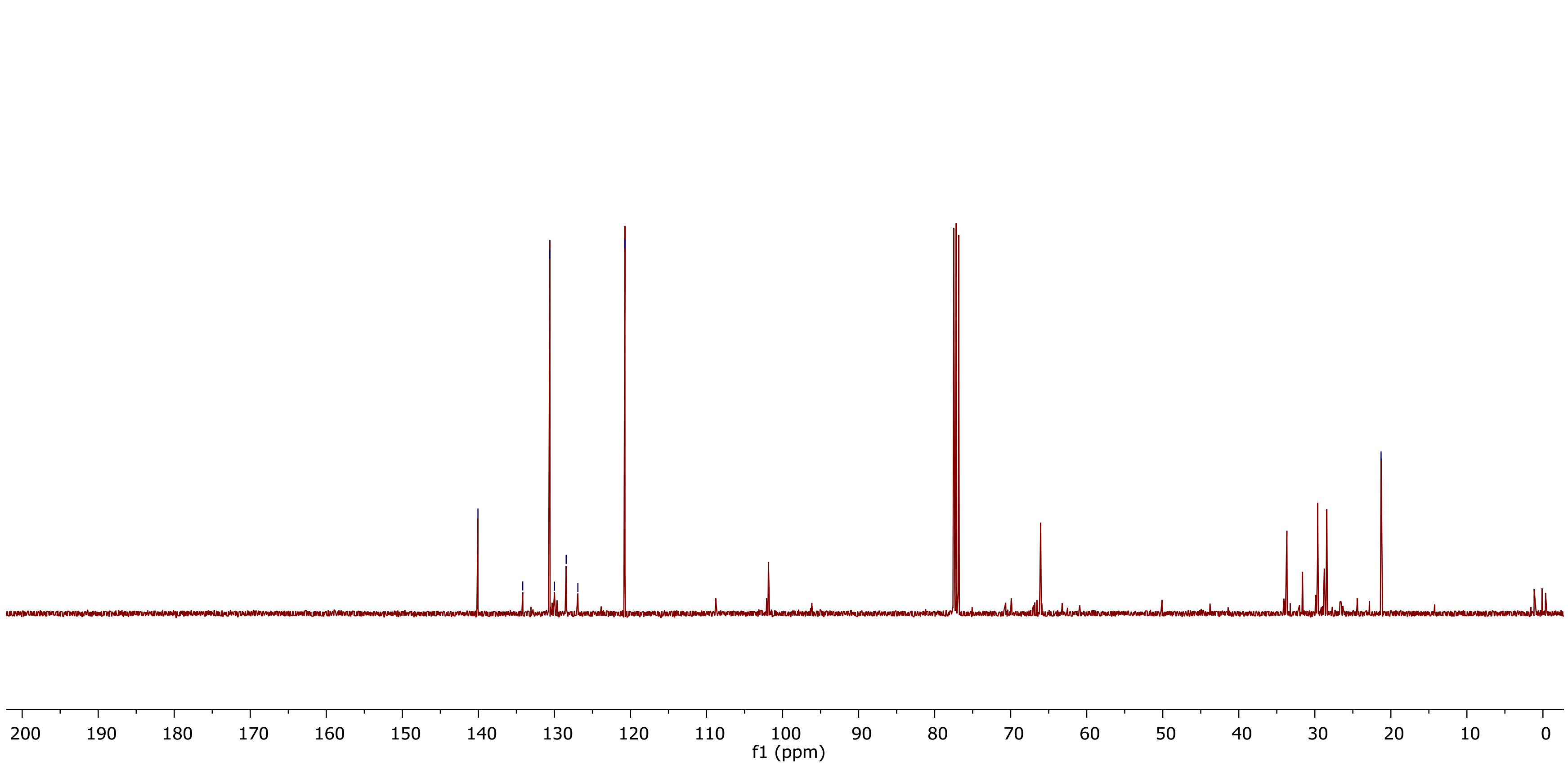
140.06
134.17
130.59
130.00
128.45
126.92
120.73
21.29
200
190
180
170
160
150
140
130
120
110
100
90
80
70
60
50
40
30
20
10
0
f1 (ppm)

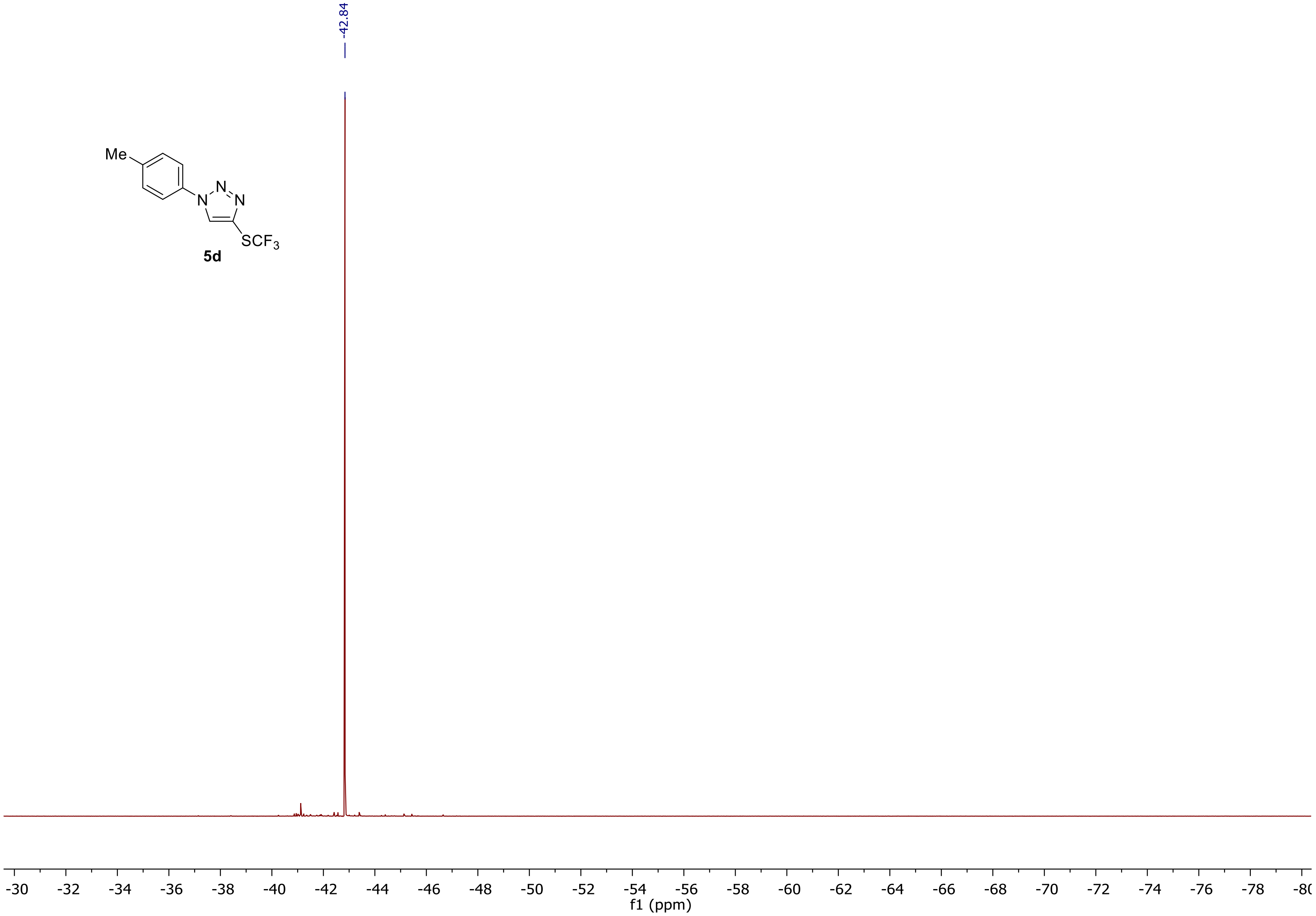

S9. ^{1}H, ^{13}C and ^{19}F NMR of Compound **5d**

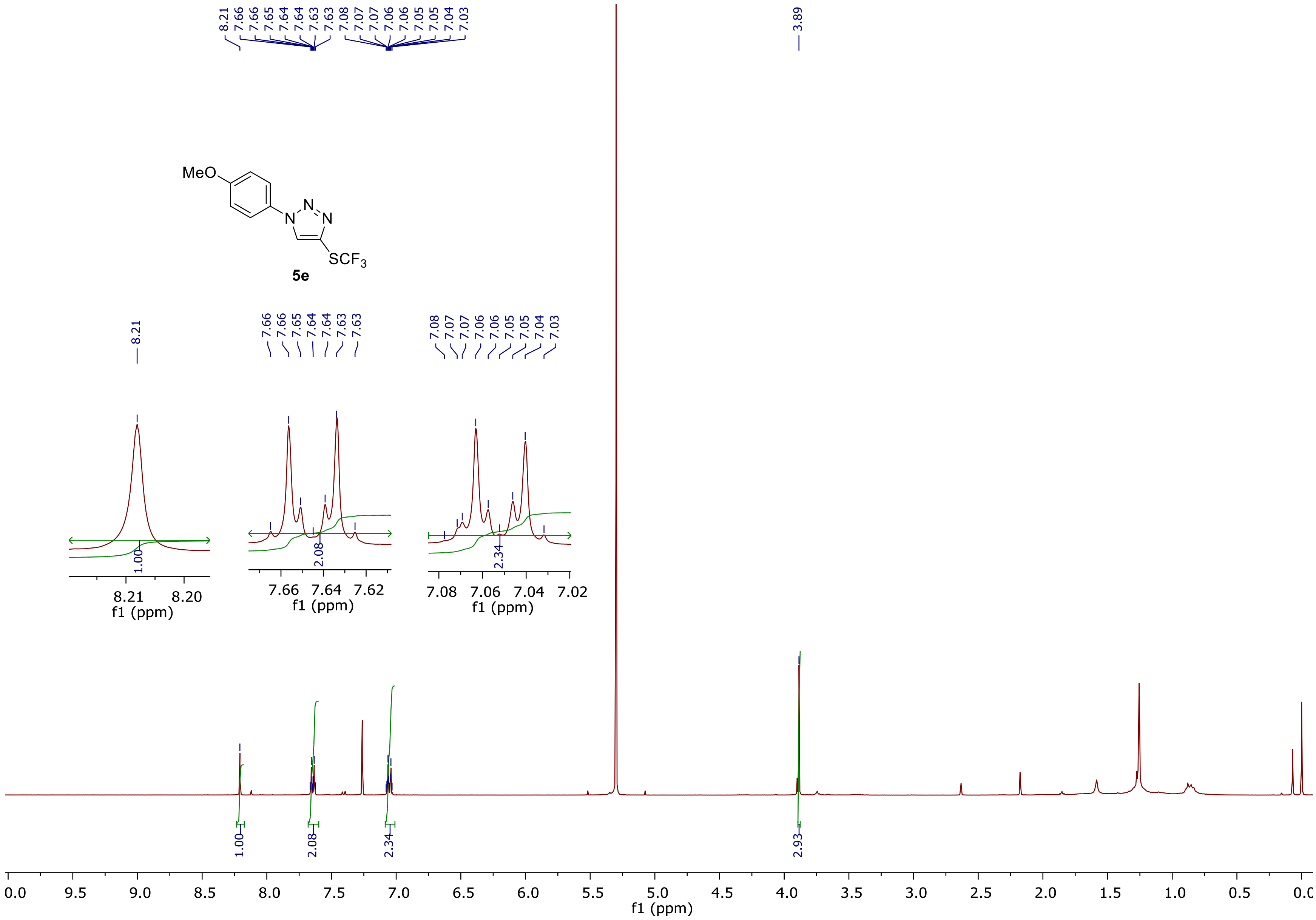

MeO
N
N
N
SCF3
5e
8.21
7.66
7.66
7.65
7.64
7.64
7.63
7.63
7.08
7.07
7.07
7.06
7.06
7.05
7.05
7.04
7.03
3.89
1.00
2.08
2.34
2.93
f1 (ppm)

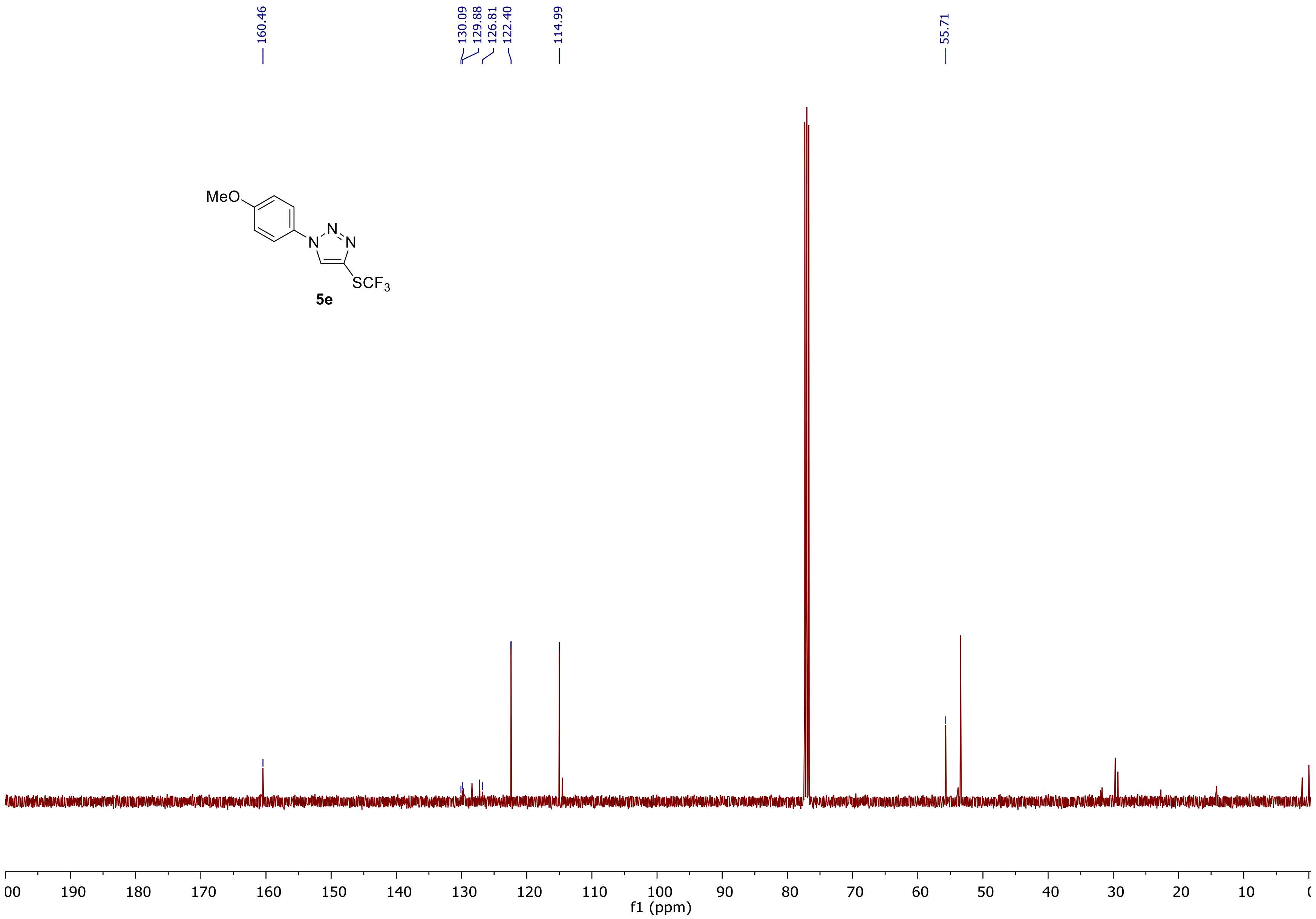

160.46
130.09
129.88
126.81
122.40
114.99
55.71
MeO
N
N
N
SCF3
00
190
180
170
160
150
140
130
120
110
100
90
80
70
60
50
40
30
20
10
f1 (ppm)

5e

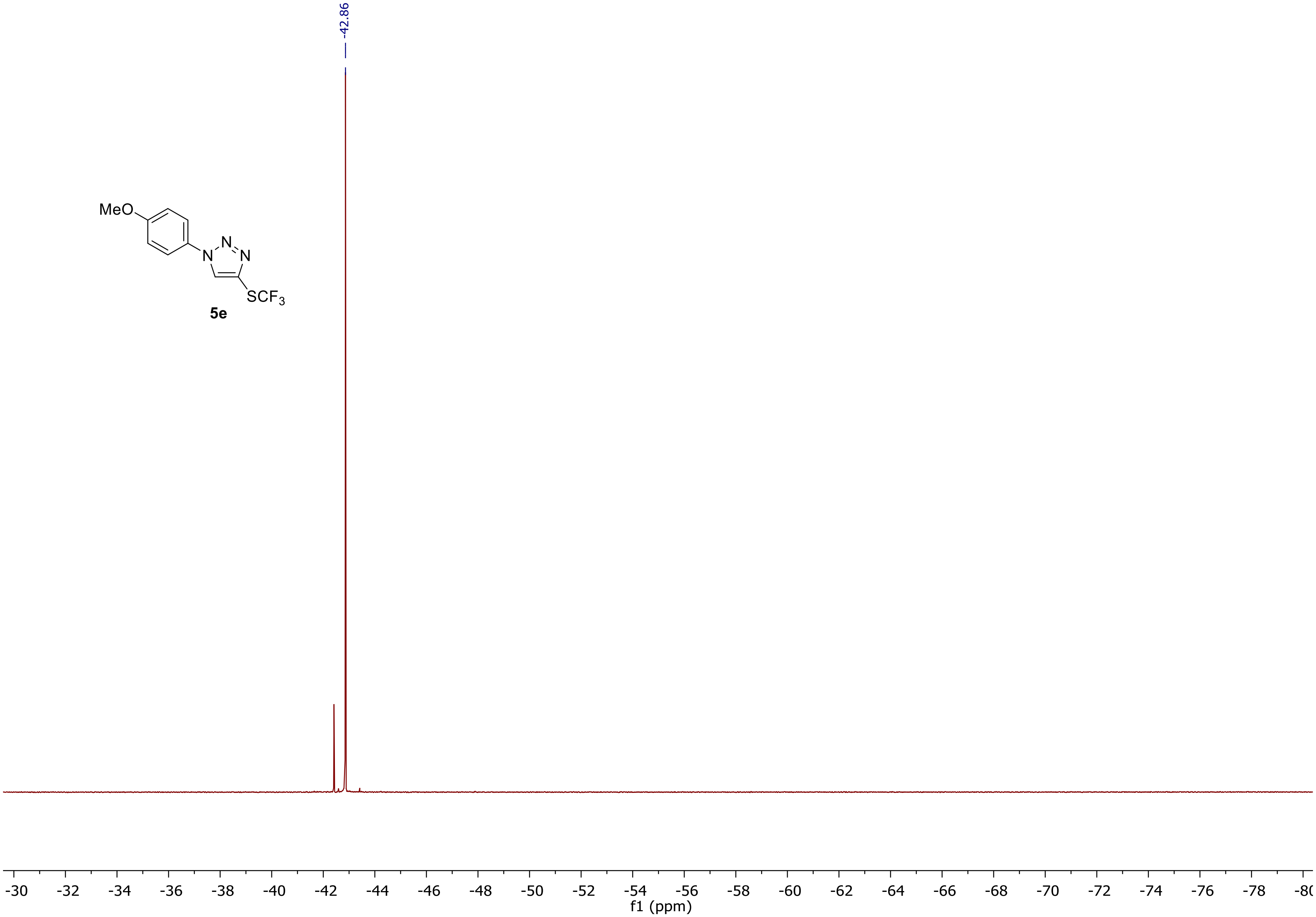

S10. ^{1}H, ^{13}C and ^{19}F NMR of Compound **5e**

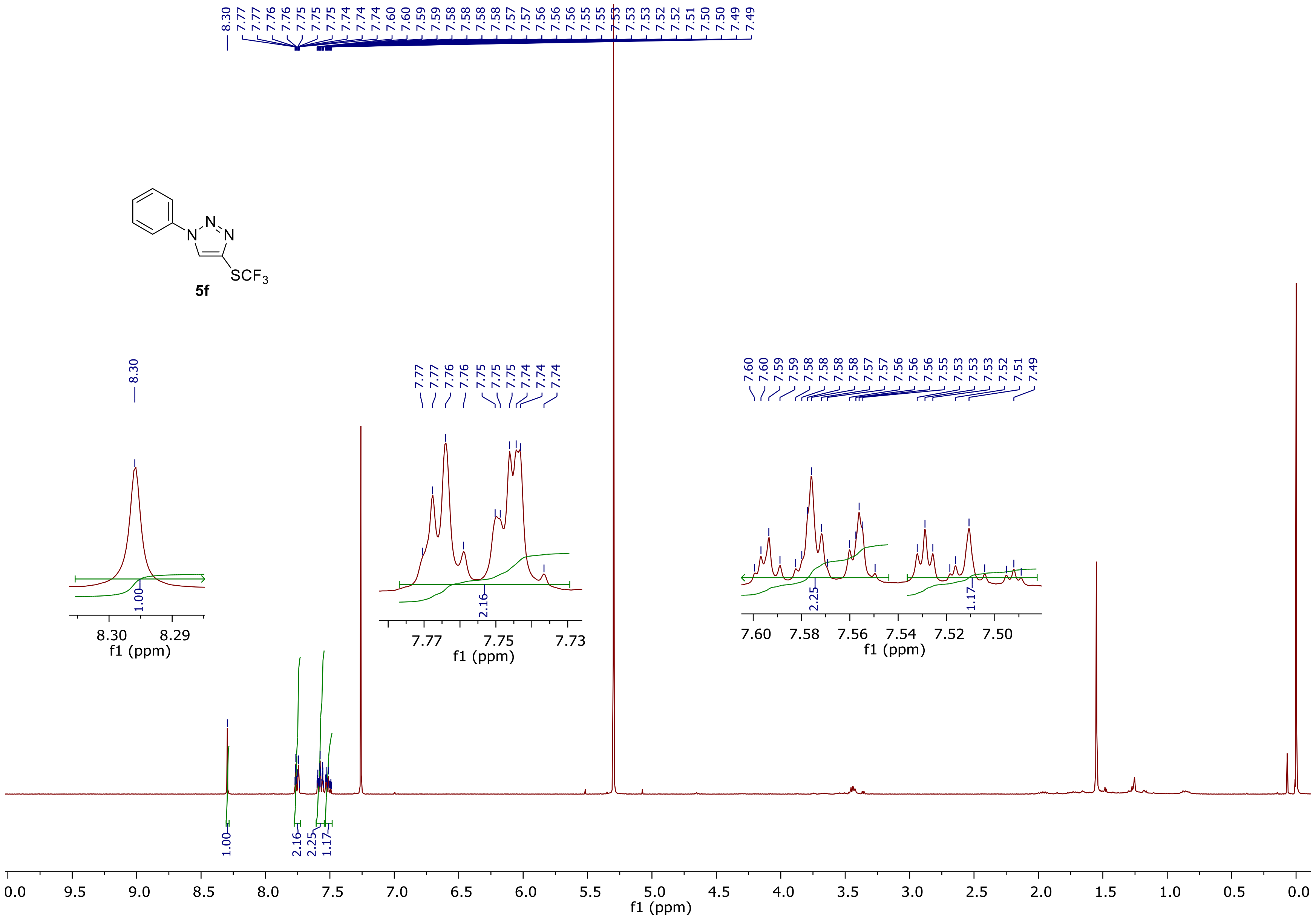

8.30
7.77 7.77 7.76 7.76 7.75 7.75 7.75 7.74 7.74 7.74 7.60 7.60 7.59 7.59 7.58 7.58 7.58 7.58 7.57 7.57 7.56 7.56 7.56 7.55 7.55 7.53 7.53 7.53 7.52 7.51 7.50 7.50 7.49 7.49
N
N
N
SCF3
5f
1.00
2.16
2.25
1.17
f1 (ppm)

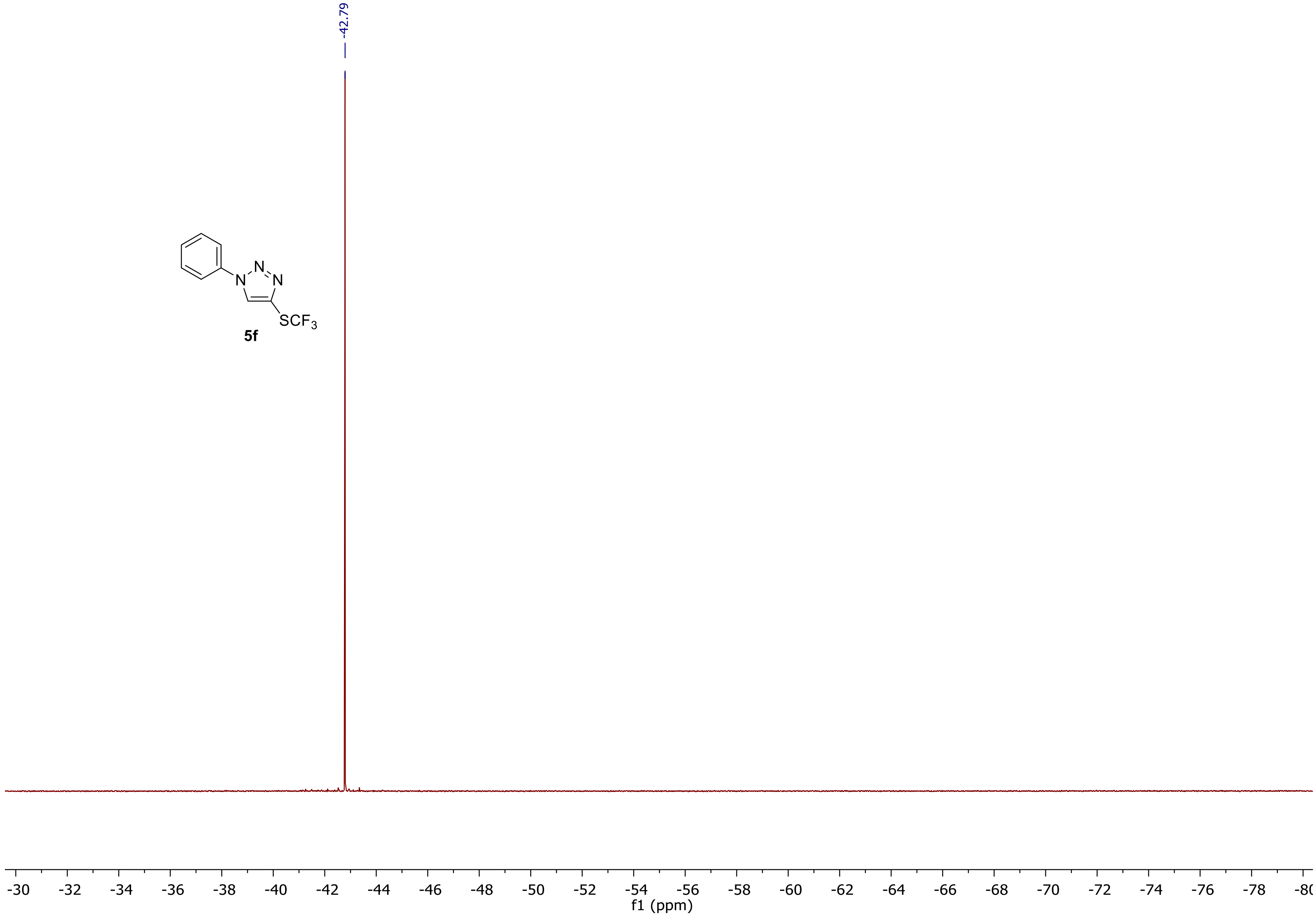
-42.79
N
N
N
SCF3
5f
-30 -32 -34 -36 -38 -40 -42 -44 -46 -48 -50 -52 -54 -56 -58 -60 -62 -64 -66 -68 -70 -72 -74 -76 -78
f1 (ppm)

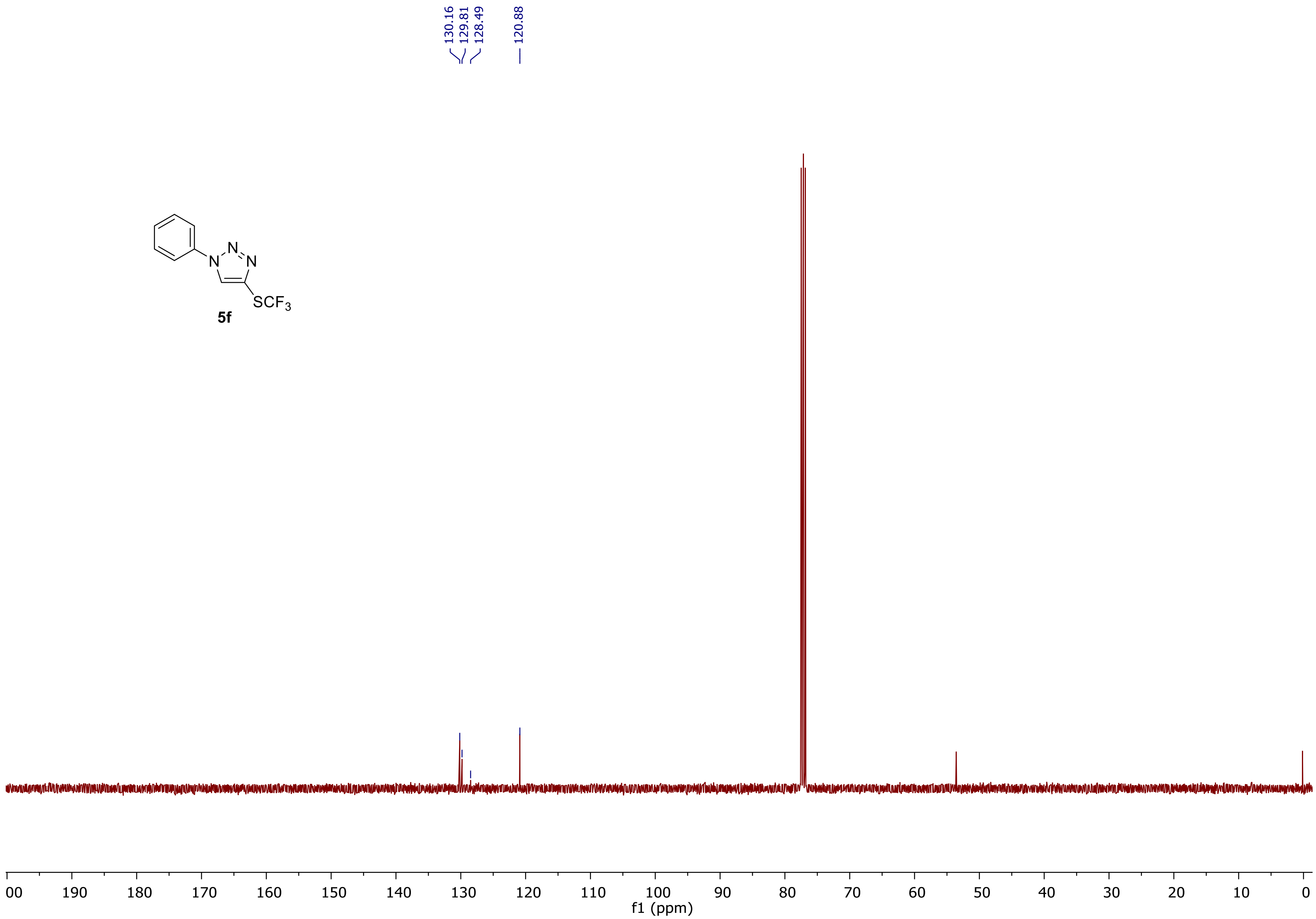

S11. ^{1}H, ^{13}C and ^{19}F NMR of Compound **5f**

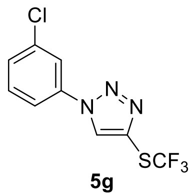
Cl
N
N
N
SCF3

5g

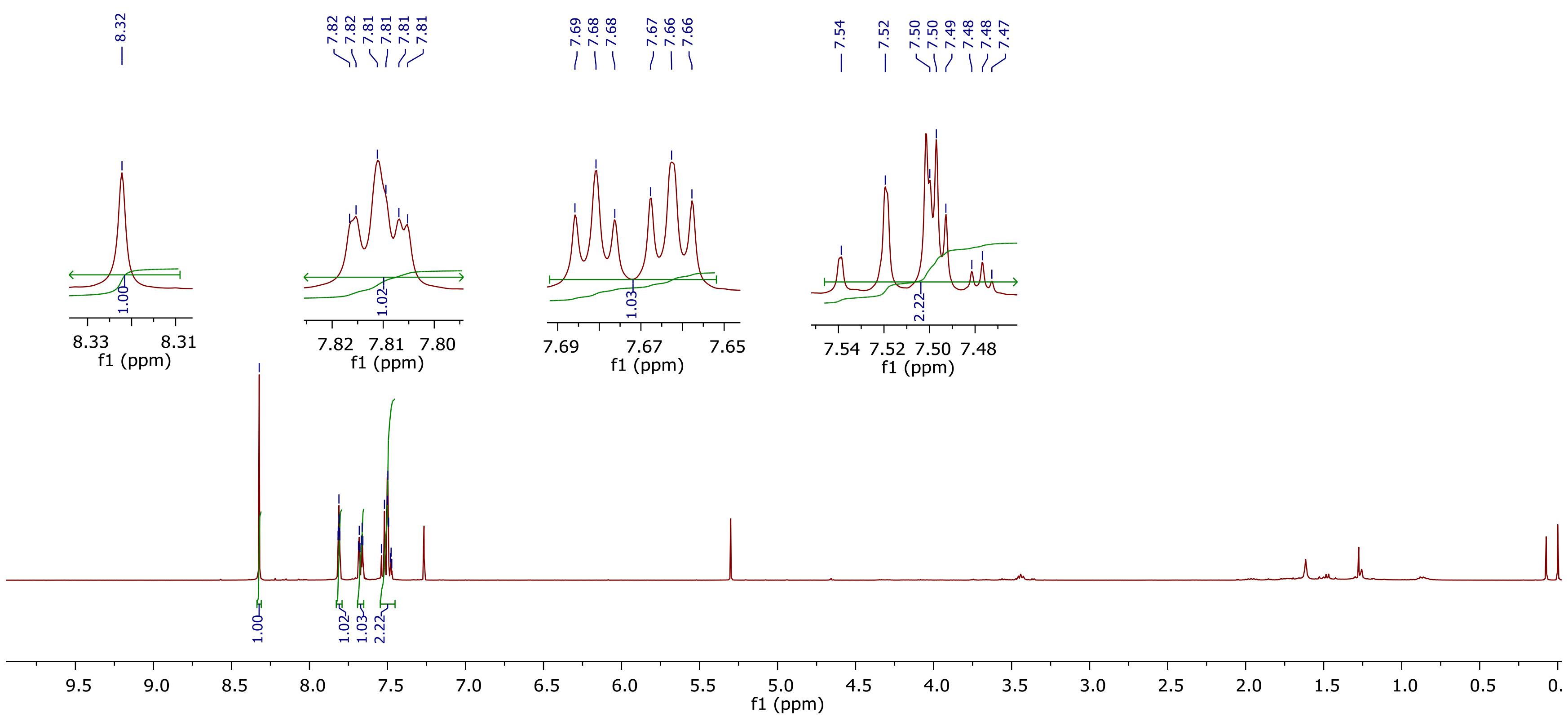
8.32
7.82
7.82
7.81
7.81
7.81
7.81
7.69
7.68
7.68
7.67
7.66
7.66
7.54
7.52
7.50
7.50
7.49
7.48
7.48
7.47
1.00
1.02
1.03
2.22
f1 (ppm)

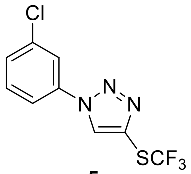
Cl
N
N
N
SCF3

5g

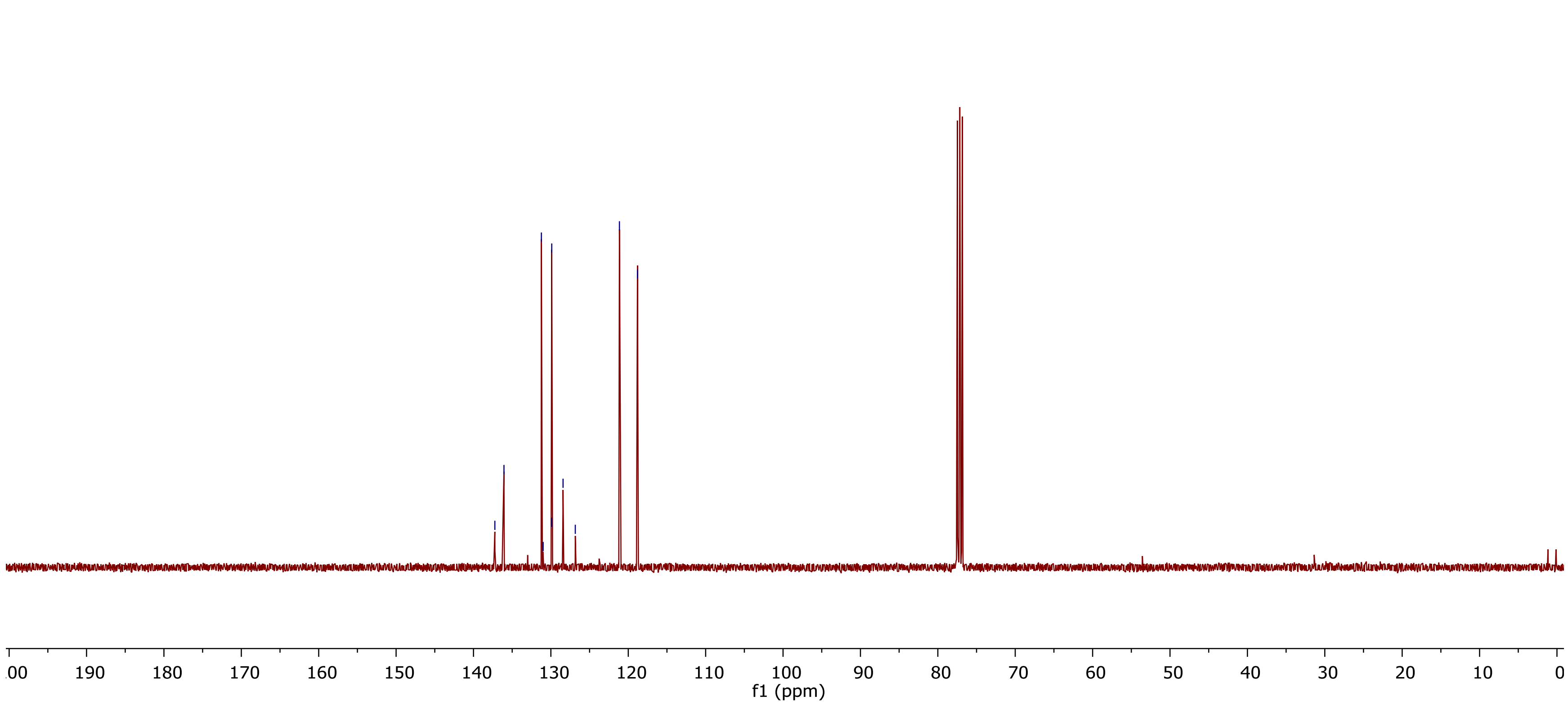
137.24
136.06
131.23
131.00
129.92
129.89
128.43
126.85
121.14
118.81
00
190
180
170
160
150
140
130
120
110
100
90
80
70
60
50
40
30
20
10
0
f1 (ppm)

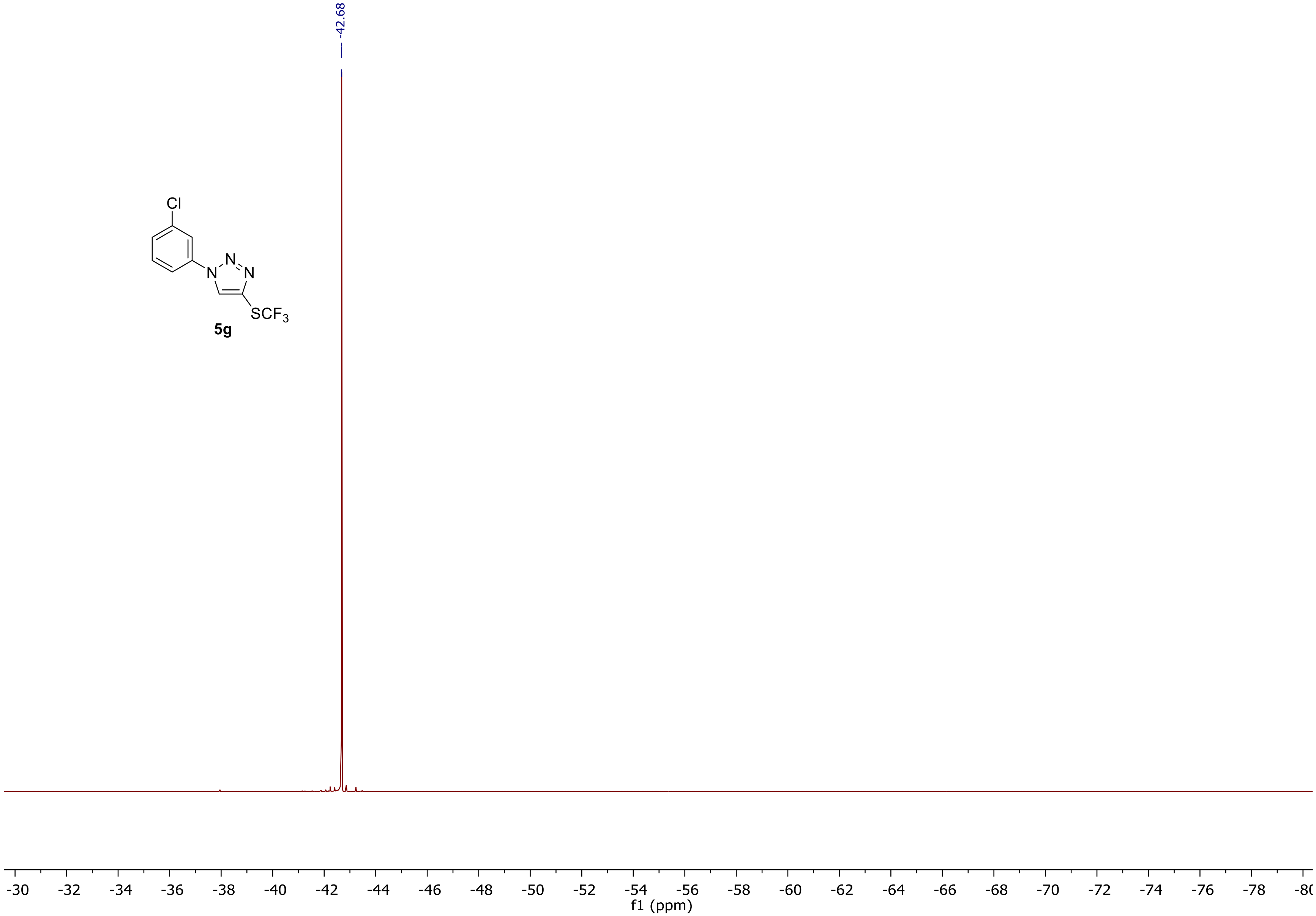

S12. ^{1}H, ^{13}C and ^{19}F NMR of Compound **5g**

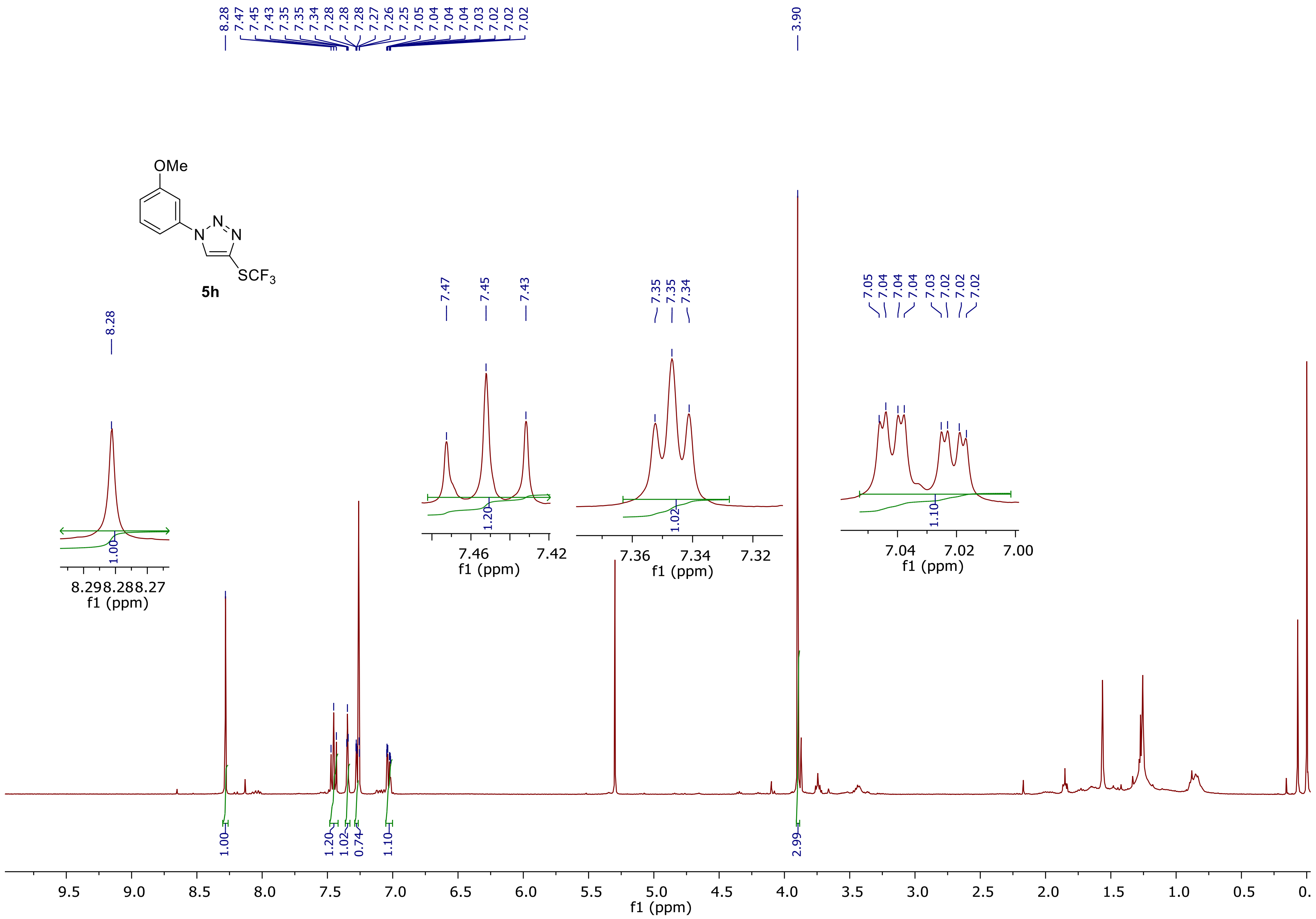

8.28
7.47
7.45
7.43
7.35
7.35
7.34
7.28
7.28
7.28
7.27
7.26
7.25
7.05
7.04
7.04
7.04
7.03
7.02
7.02
7.02
3.90
OMe
N
N
N
SCF3
5h
1.00
1.20
1.02
0.74
1.10
2.99
8.29 8.28 8.27
7.46
7.42
7.36
7.34
7.32
7.04
7.02
7.00
f1 (ppm)
9.5
9.0
8.5
8.0
7.5
7.0
6.5
6.0
5.5
5.0
4.5
4.0
3.5
3.0
2.5
2.0
1.5
1.0
0.5
0.

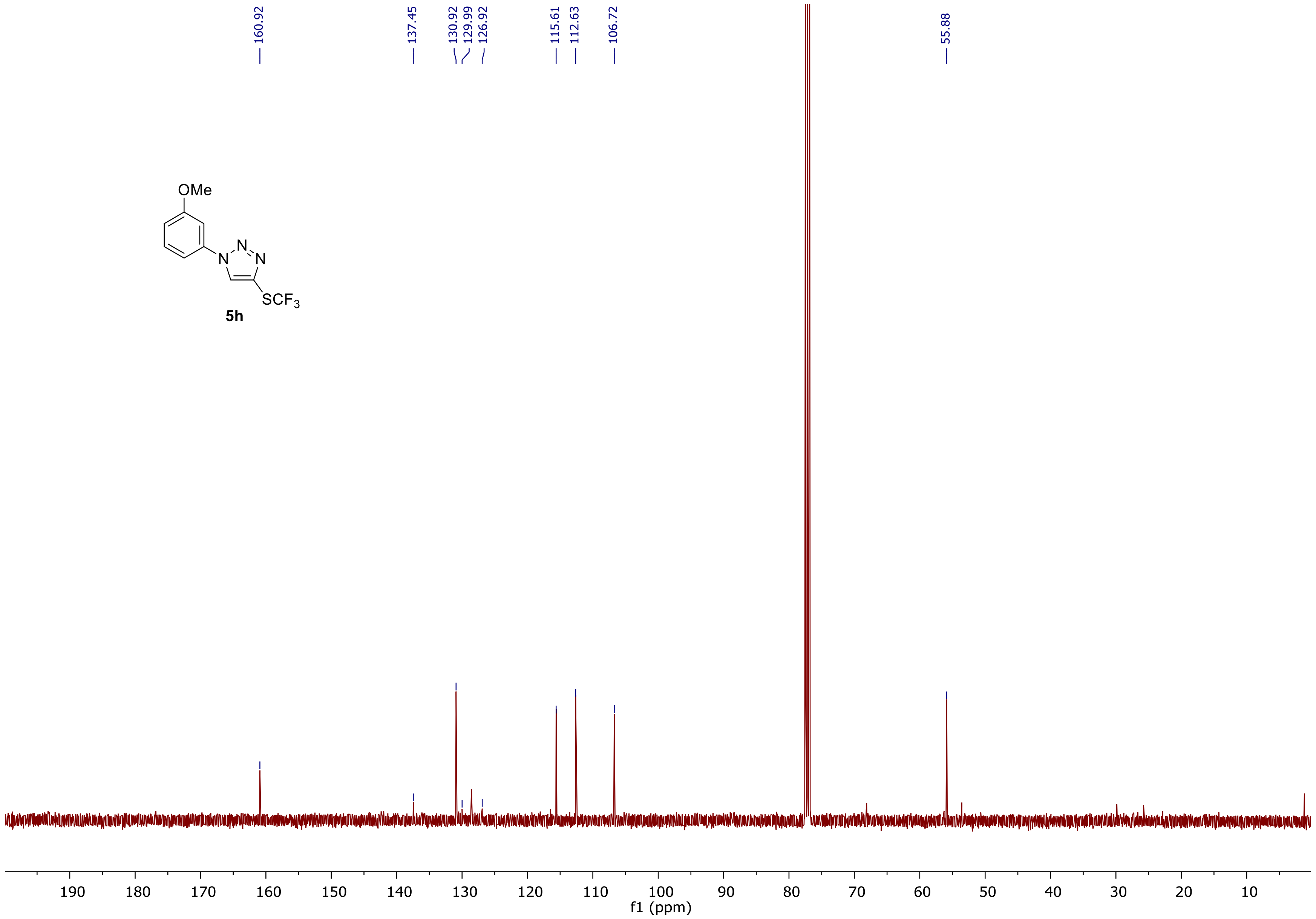

160.92
137.45
130.92
129.99
126.92
115.61
112.63
106.72
55.88
OMe
N
N
N
SCF3
5h
190 180 170 160 150 140 130 120 110 100 90 80 70 60 50 40 30 20 10
f1 (ppm)

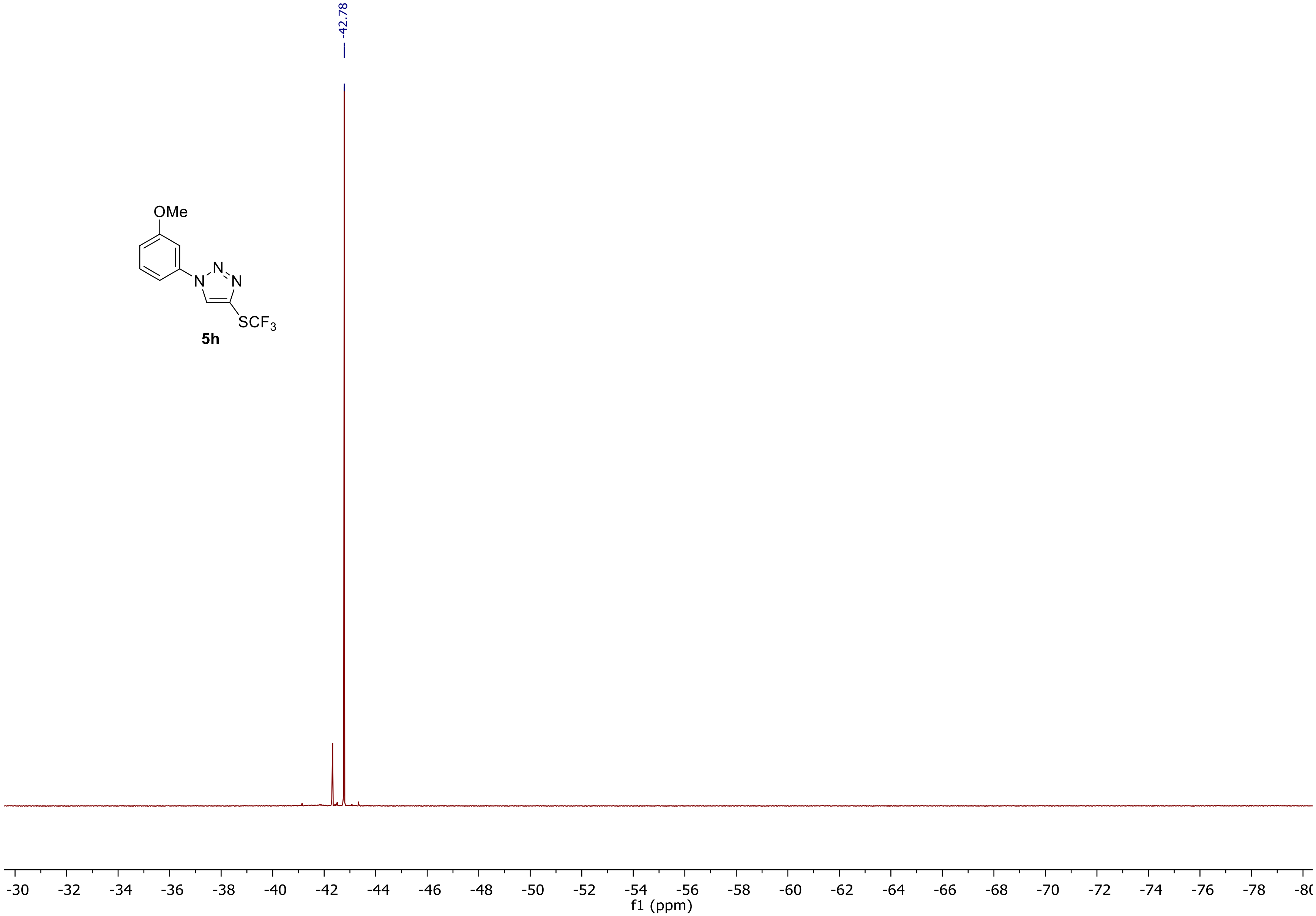

S13. ^{1}H, ^{13}C and ^{19}F NMR of Compound **5h**

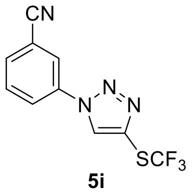
CN
N
N
N
SCF3

5i

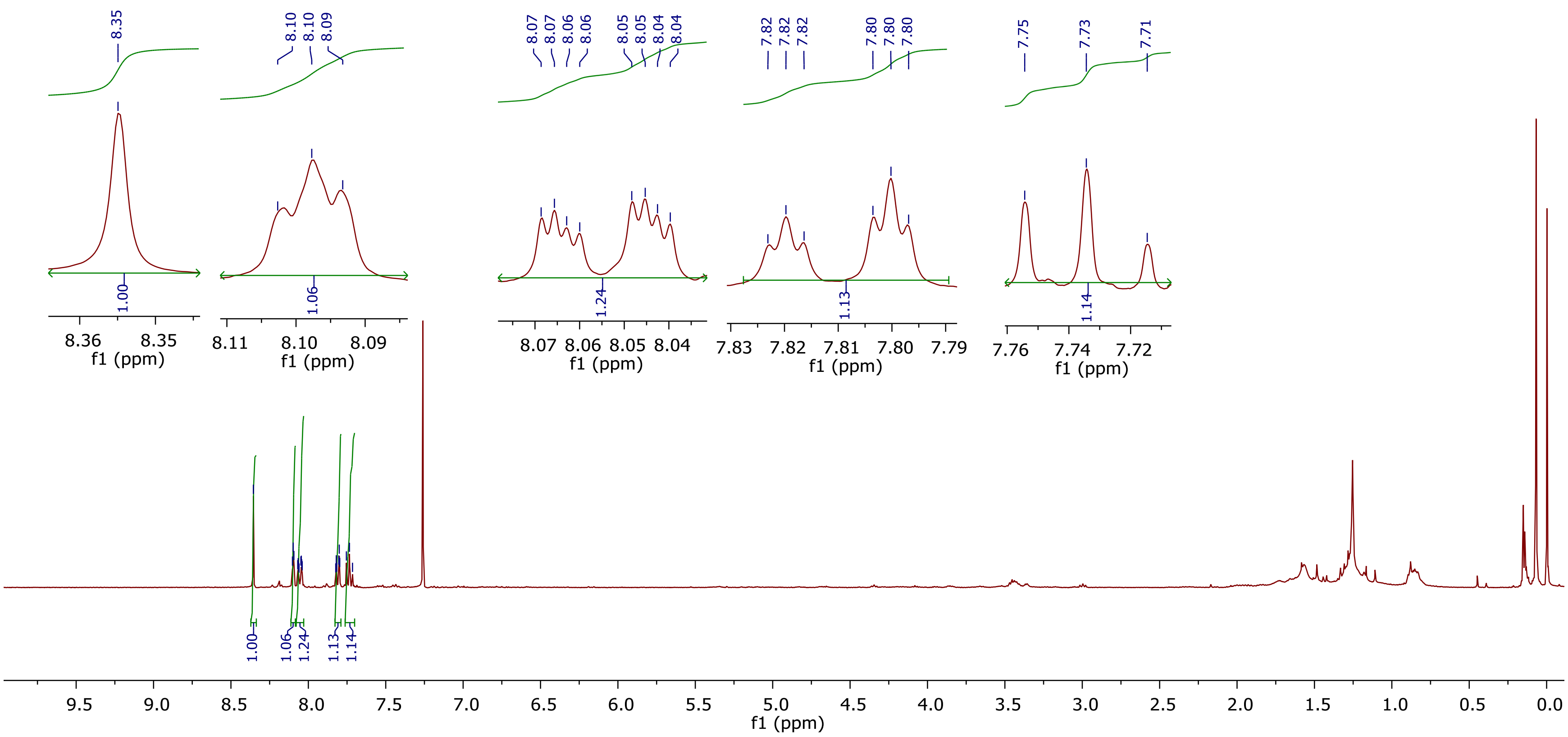
8.35
8.10
8.10
8.09
8.07
8.07
8.06
8.06
8.05
8.05
8.04
8.04
7.82
7.82
7.82
7.80
7.80
7.80
7.75
7.73
7.71
1.00
1.06
1.24
1.13
1.14
f1 (ppm)

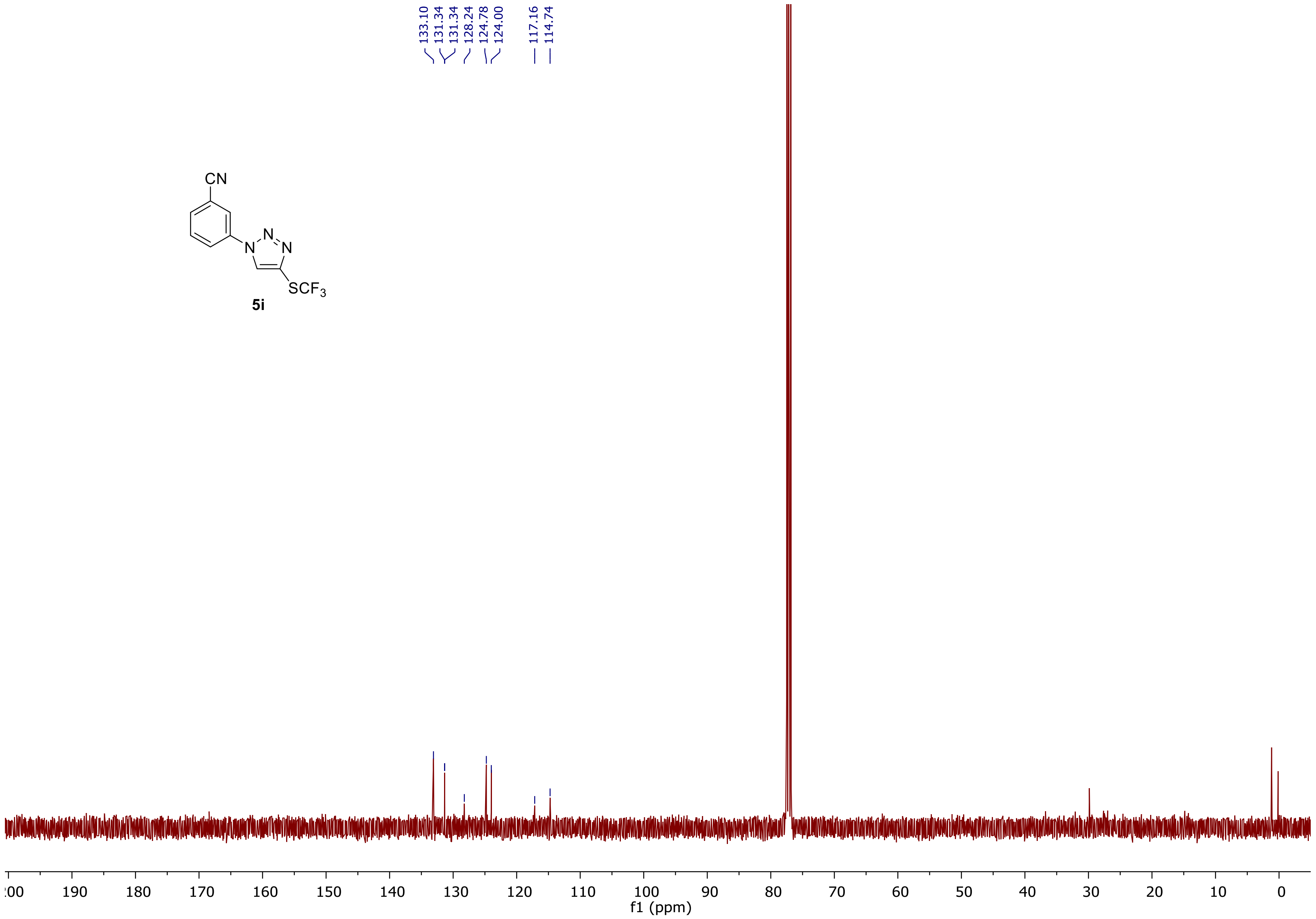

133.10
131.34
131.34
128.24
124.78
124.00
117.16
114.74
CN
N
N
N
SCF3
5i
200
190
180
170
160
150
140
130
120
110
100
90
80
70
60
50
40
30
20
10
0
f1 (ppm)

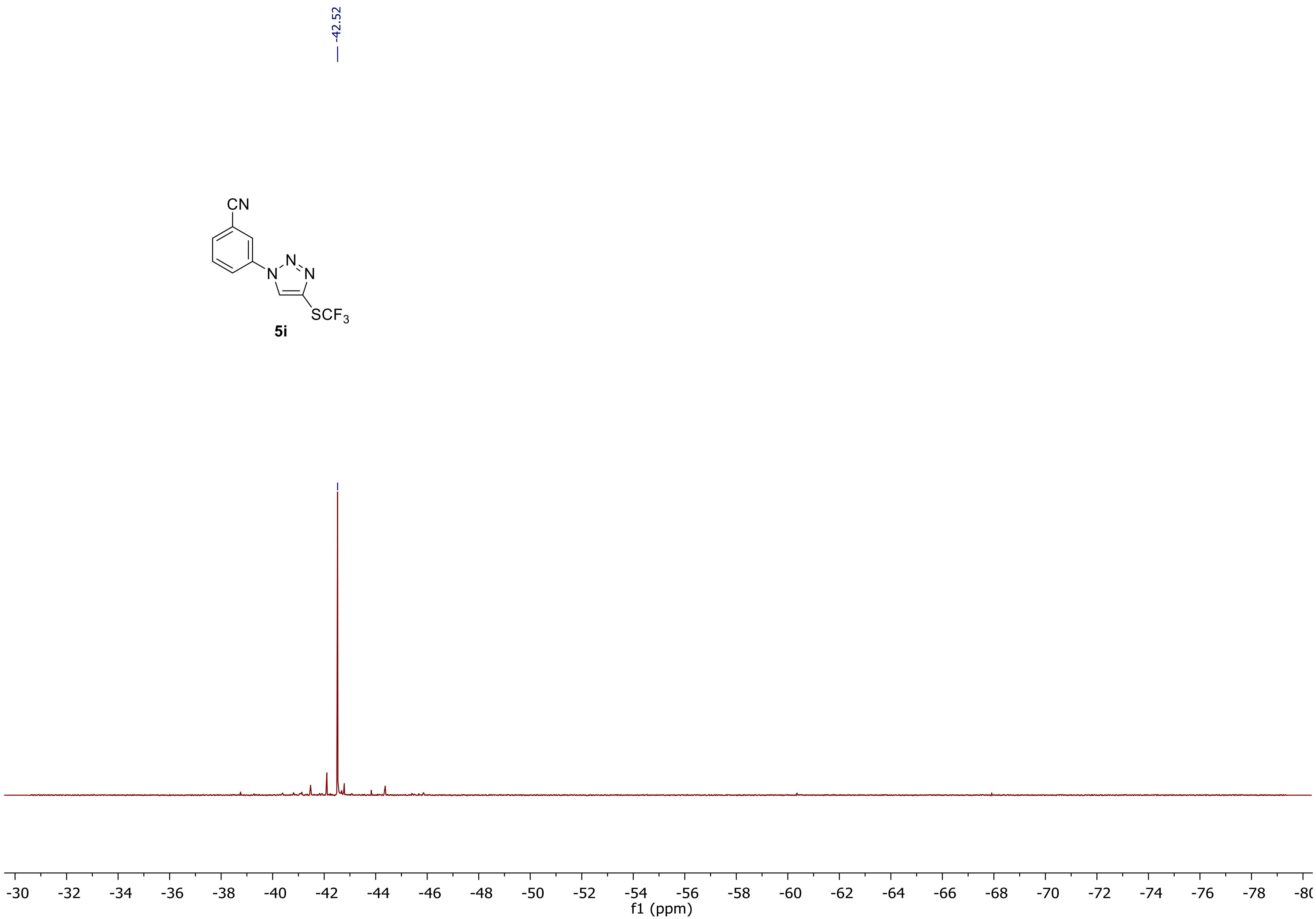

S14. ^{1}H, ^{13}C and ^{19}F NMR of Compound **5i**

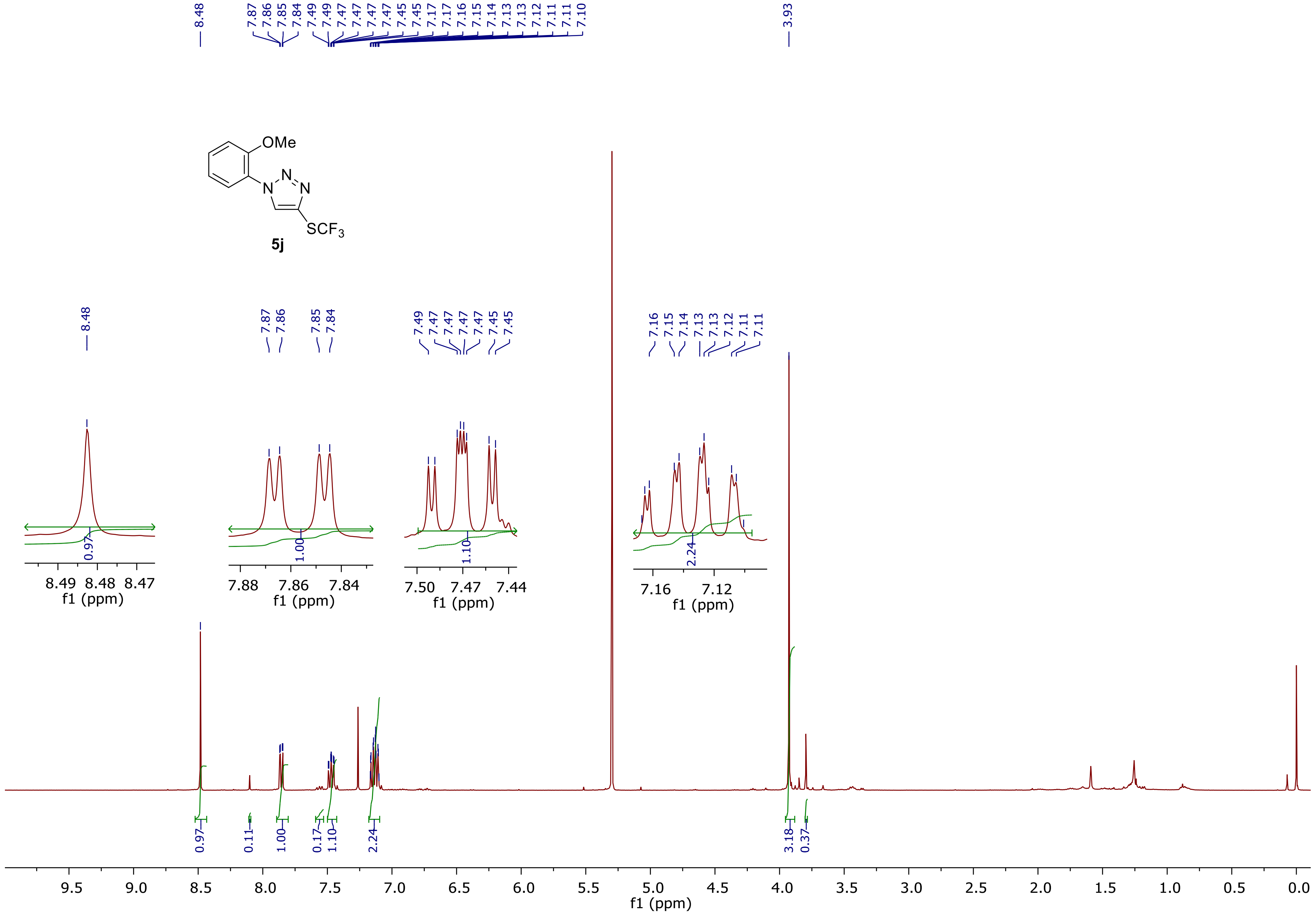

5j

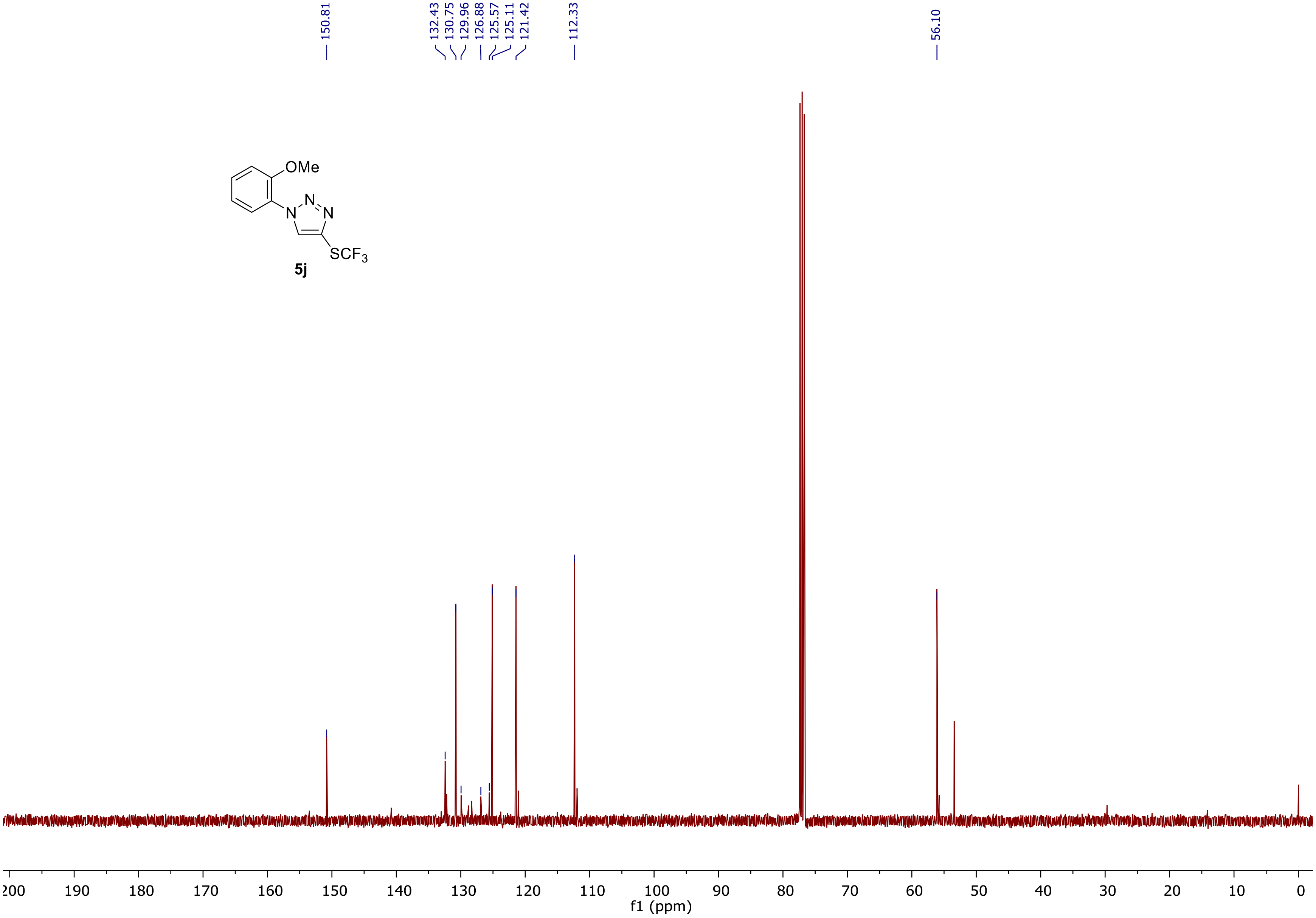

OMe
N
N
N
SCF3
5j
150.81
132.43
130.75
129.96
126.88
125.57
125.11
121.42
112.33
56.10
200
190
180
170
160
150
140
130
120
110
100
90
80
70
60
50
40
30
20
10
0
f1 (ppm)

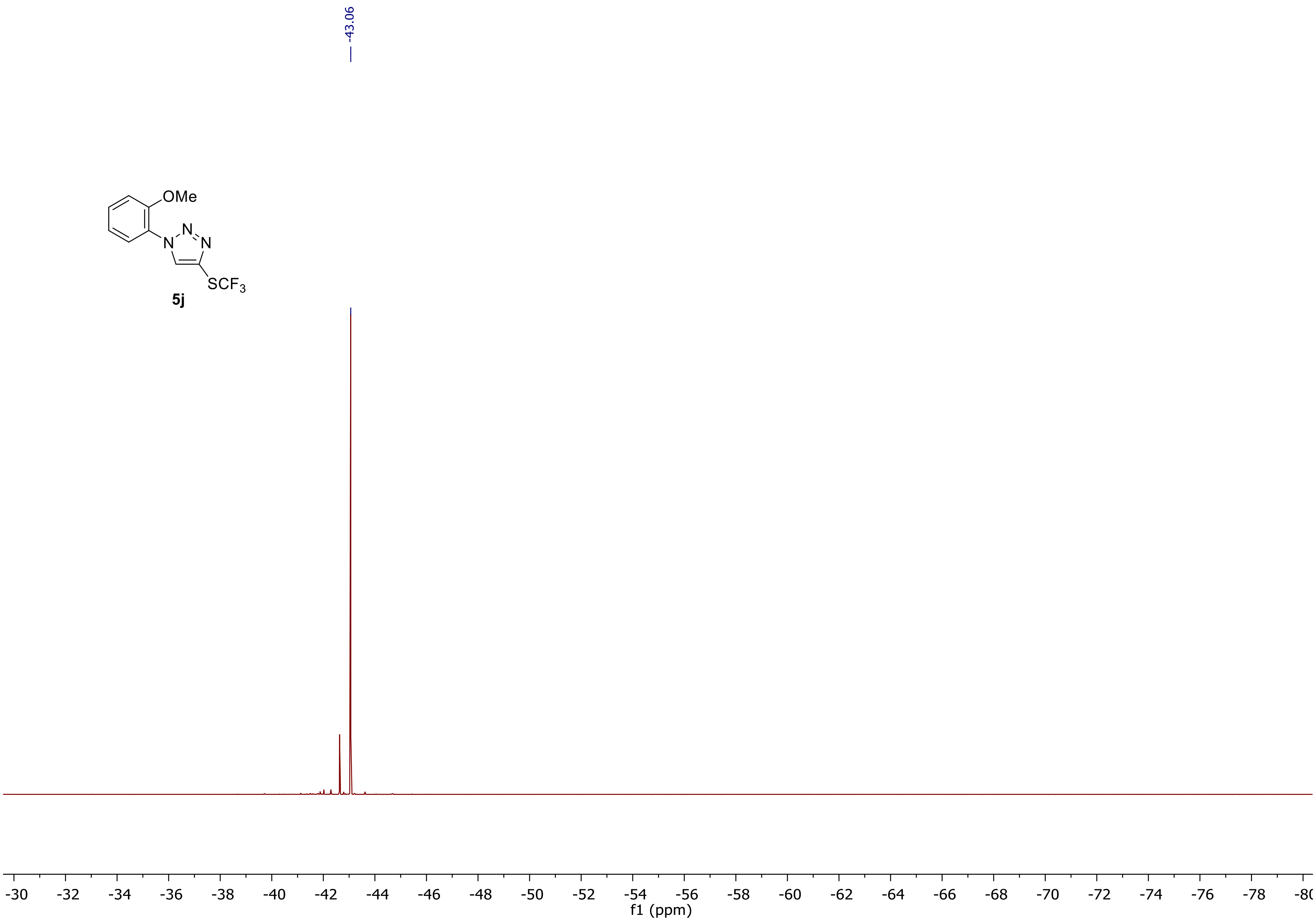

S15. ^{1}H, ^{13}C and ^{19}F NMR of Compound **5j**

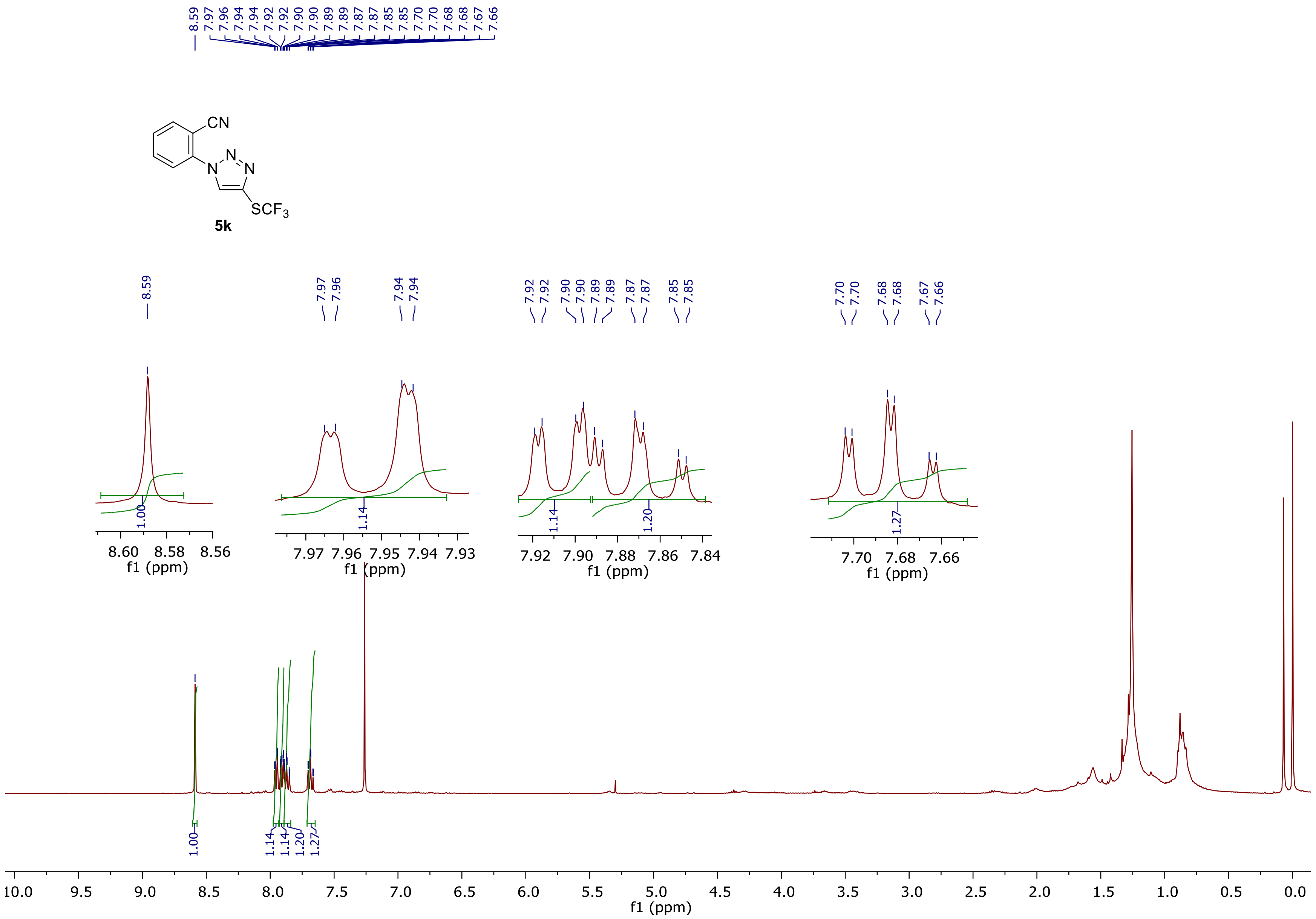
8.59
7.97
7.96
7.94
7.94
7.92
7.92
7.90
7.90
7.89
7.89
7.87
7.87
7.85
7.85
7.70
7.70
7.68
7.68
7.67
7.66
CN
N
N
N
SCF3
5k
1.00
1.14
1.14
1.20
1.27
f1 (ppm)

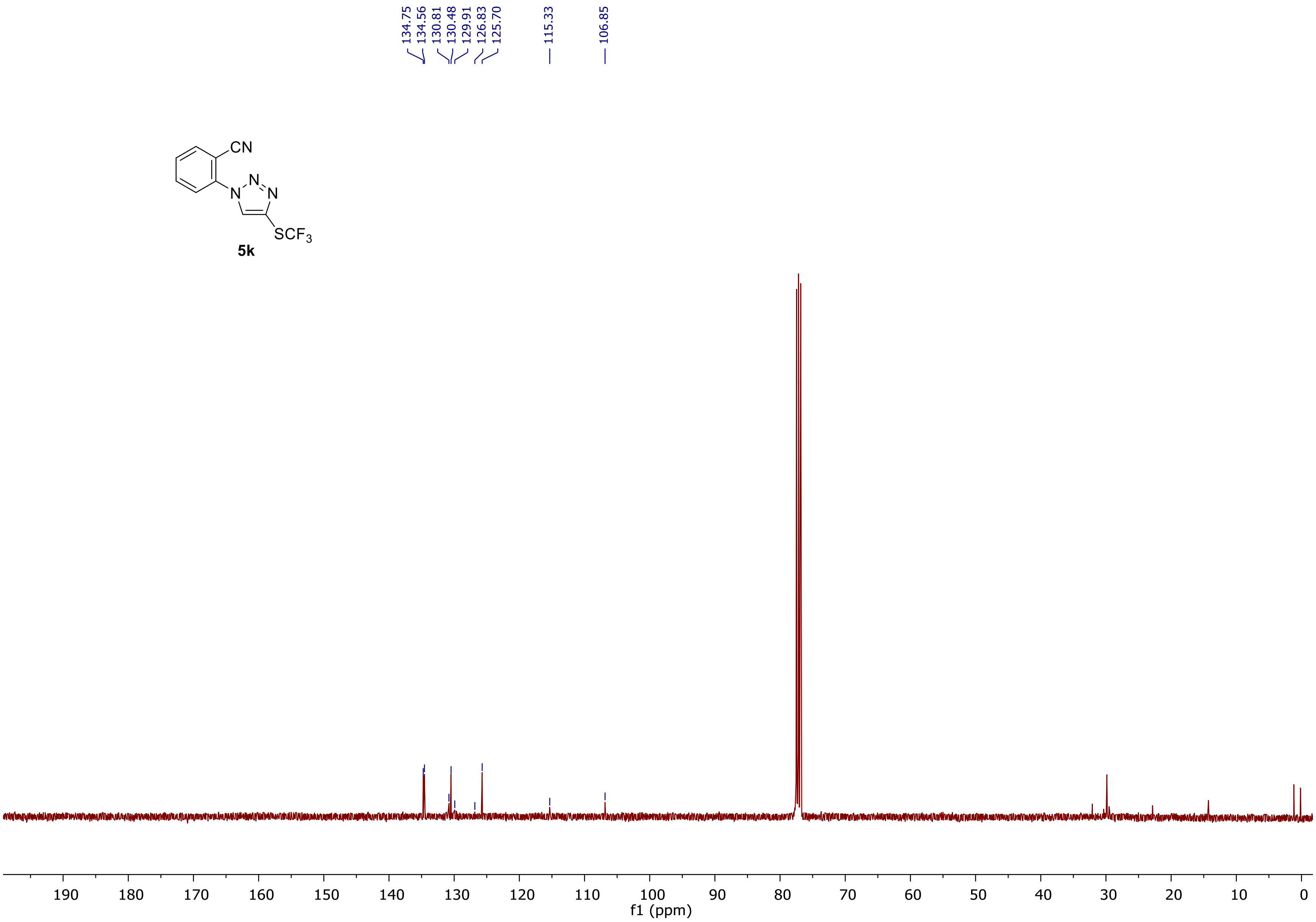
134.75
134.56
130.81
130.48
129.91
126.83
125.70
115.33
106.85
CN
N
N
N
SCF3
5k
190
180
170
160
150
140
130
120
110
100
90
80
70
60
50
40
30
20
10
0
f1 (ppm)

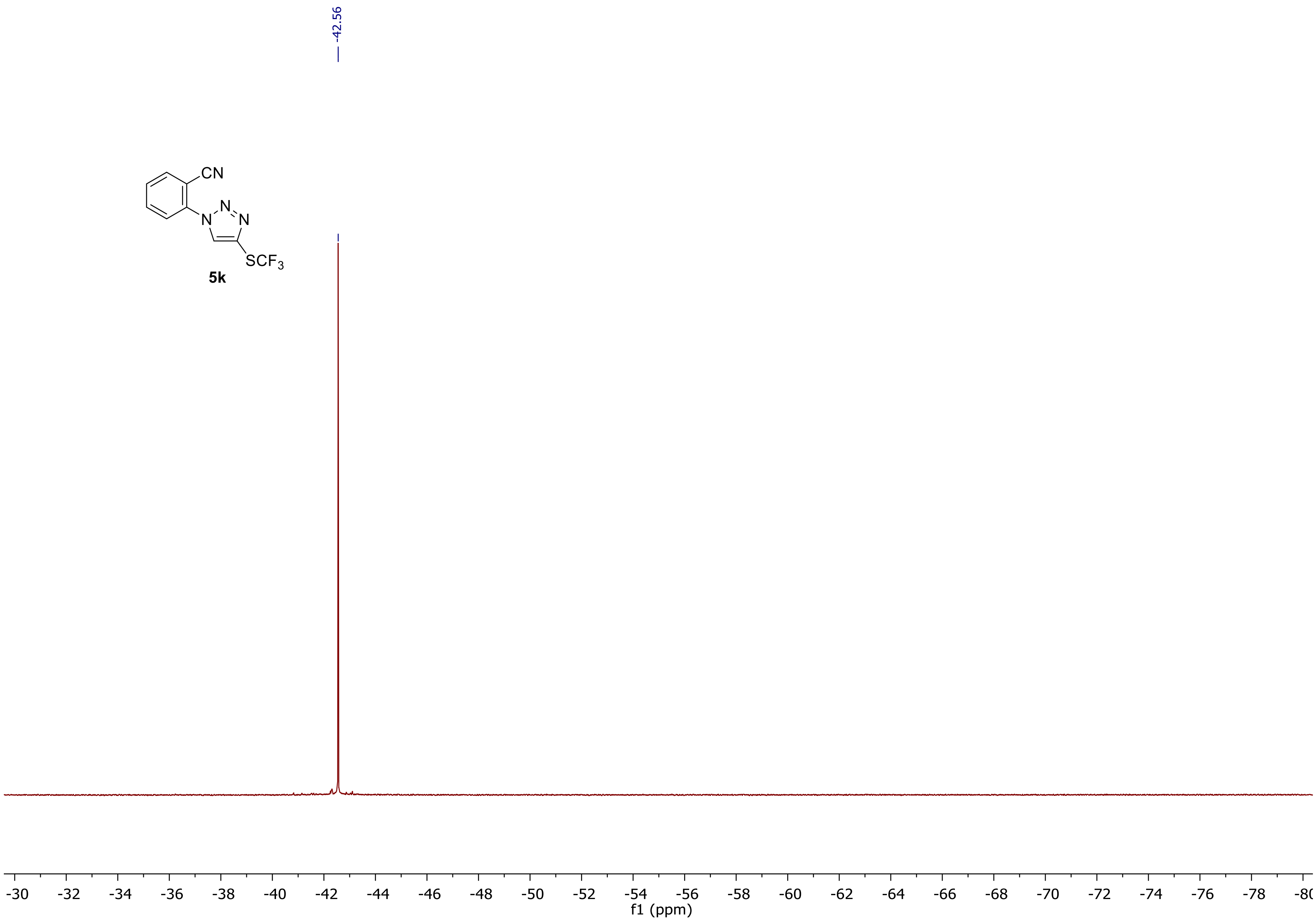

S16. ^{1}H, ^{13}C and ^{19}F NMR of Compound **5k**

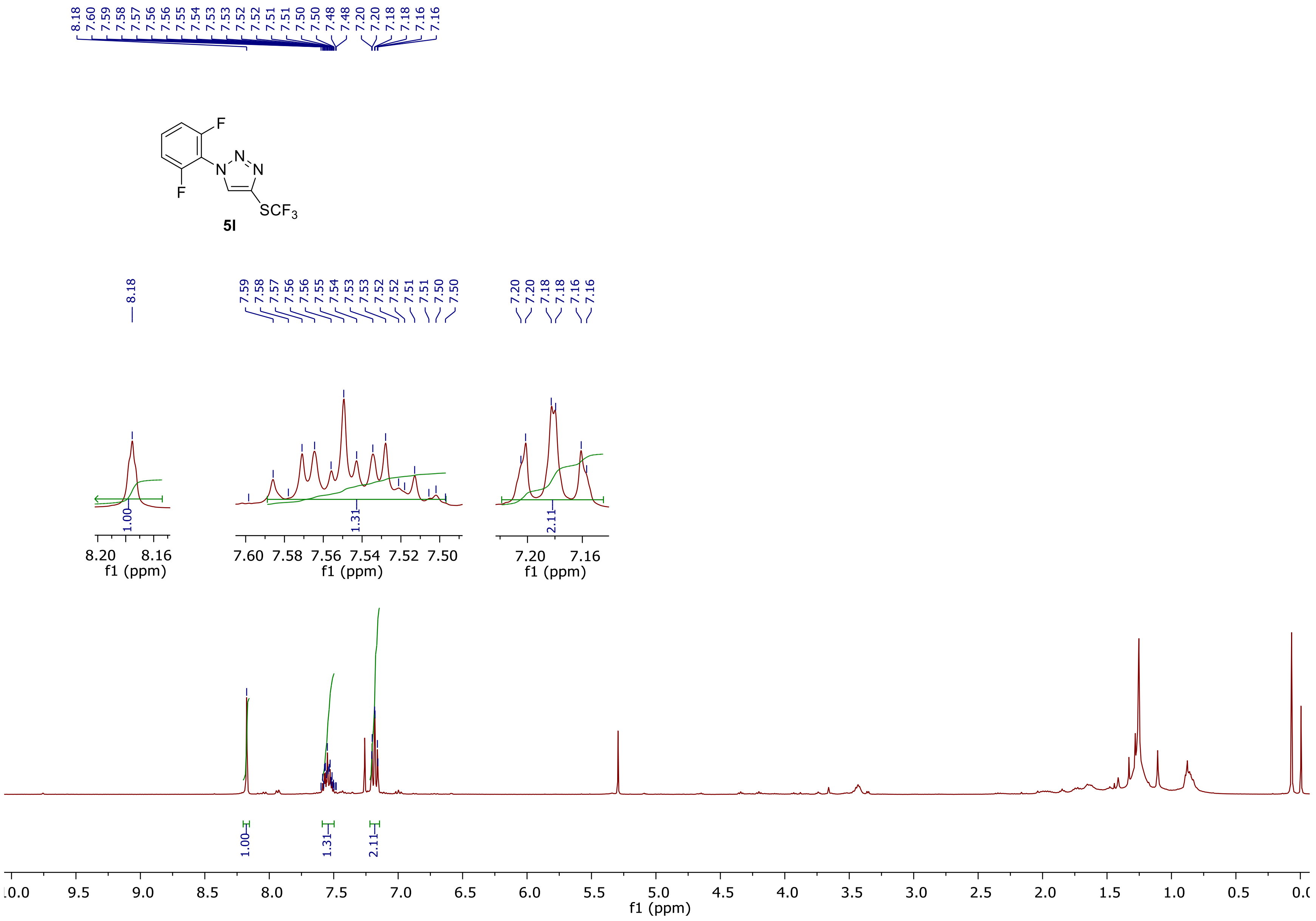
8.18 7.60 7.59 7.58 7.57 7.56 7.56 7.55 7.54 7.53 7.53 7.52 7.52 7.51 7.51 7.50 7.50 7.48 7.48 7.20 7.20 7.18 7.18 7.16 7.16
F
N
N
N
F
SCF3
5l
1.00
1.31
2.11
f1 (ppm)

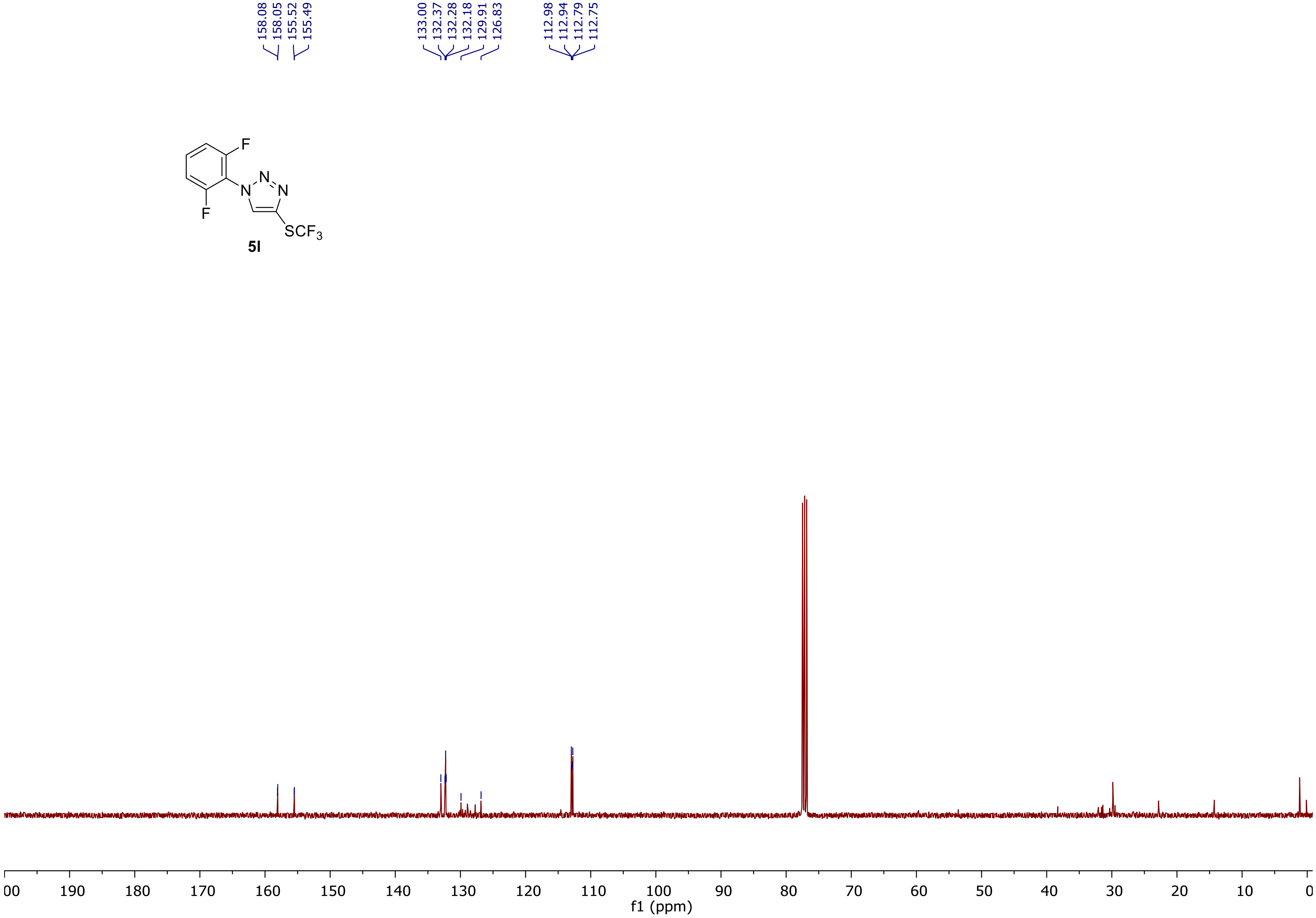

158.08
158.05
155.52
155.49
133.00
132.37
132.28
132.18
129.91
126.83
112.98
112.94
112.79
112.75
F
N
N
N
F
SCF3
5l
00
190
180
170
160
150
140
130
120
110
100
90
80
70
60
50
40
30
20
10
0
f1 (ppm)

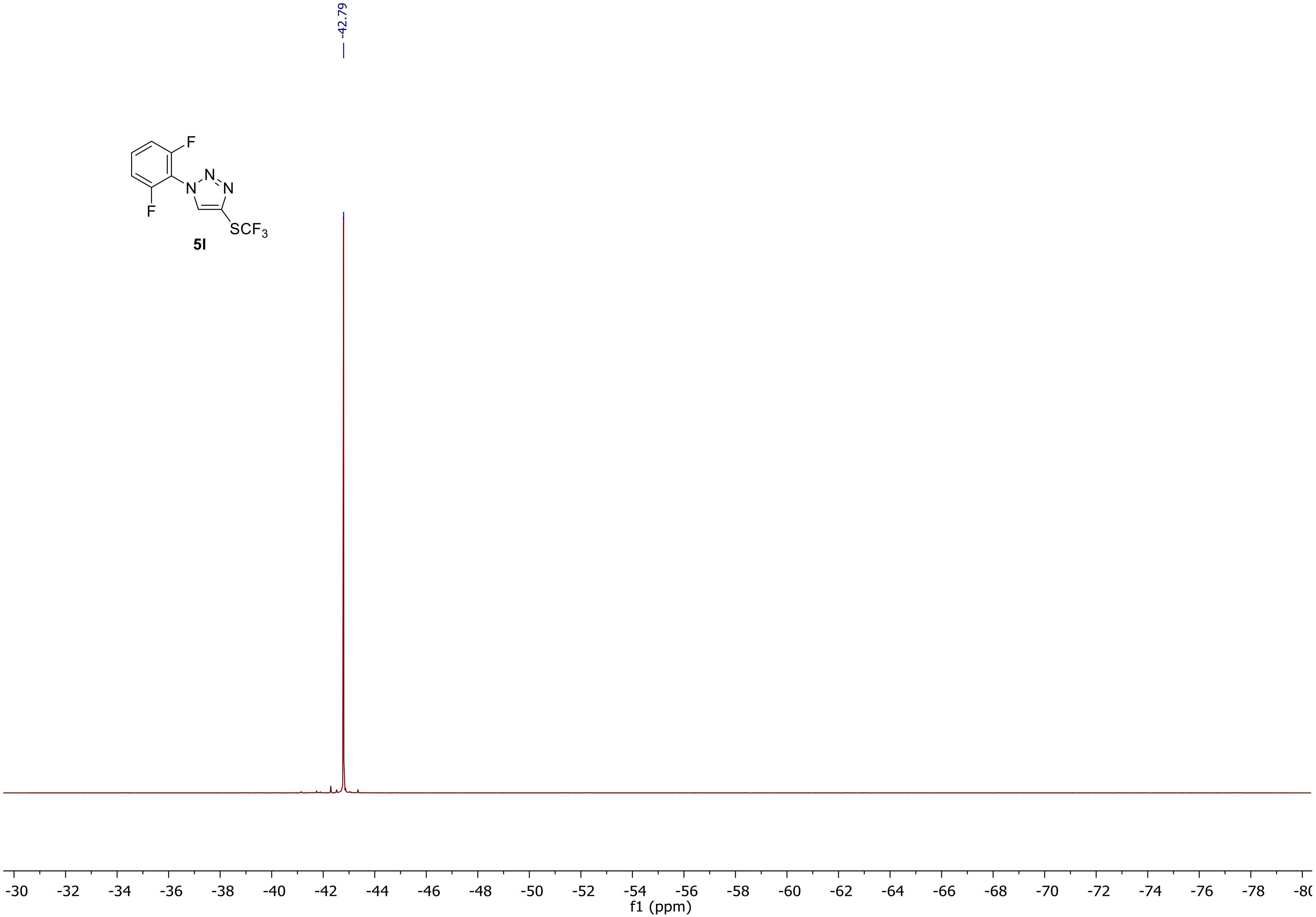

S17. ^{1}H, ^{13}C and ^{19}F NMR of Compound **5l**

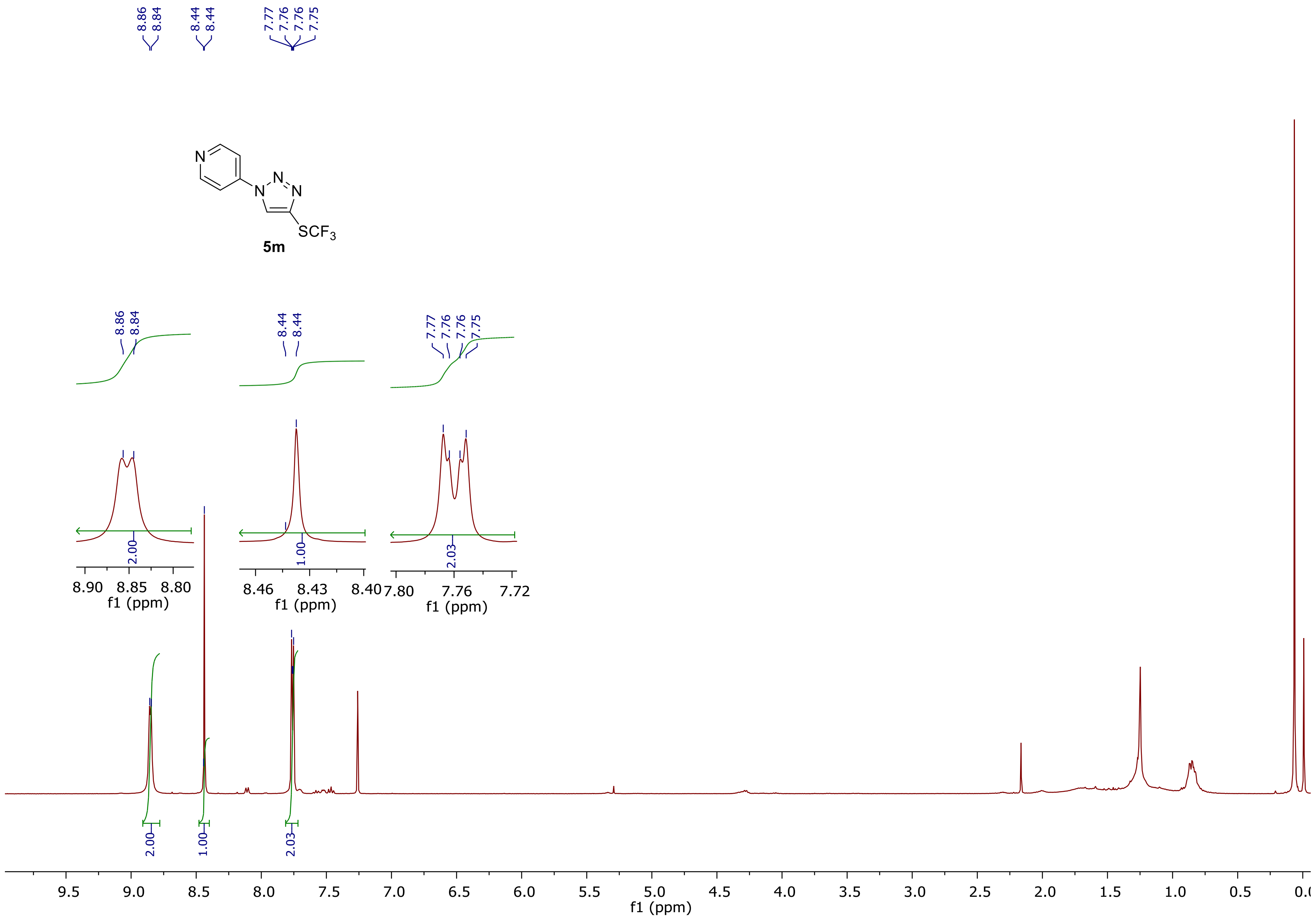

5m
SCF3
f1 (ppm)

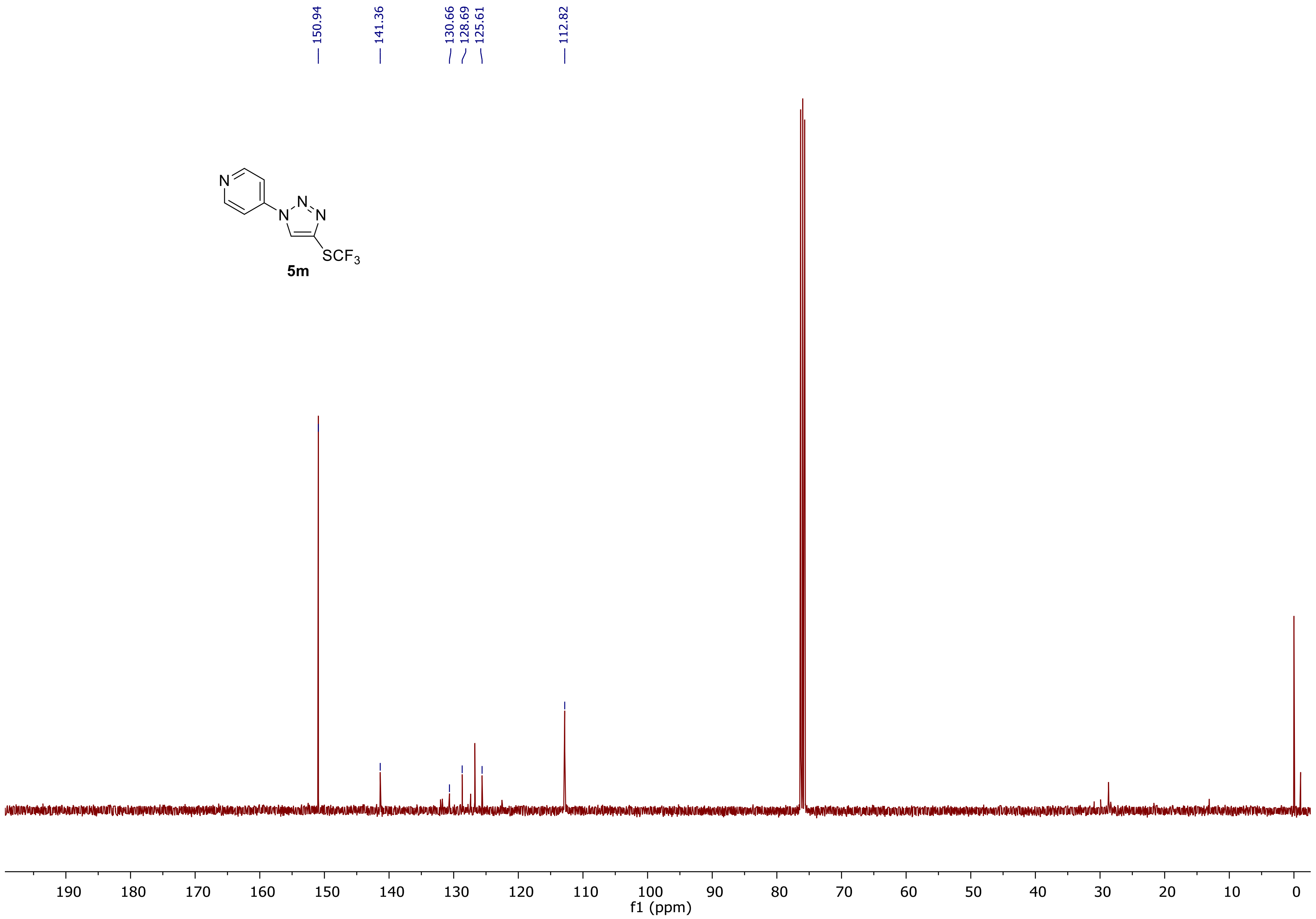
N
N
N
N
SCF3
5m
150.94
141.36
130.66
128.69
125.61
112.82
190
180
170
160
150
140
130
120
110
100
90
80
70
60
50
40
30
20
10
0
f1 (ppm)

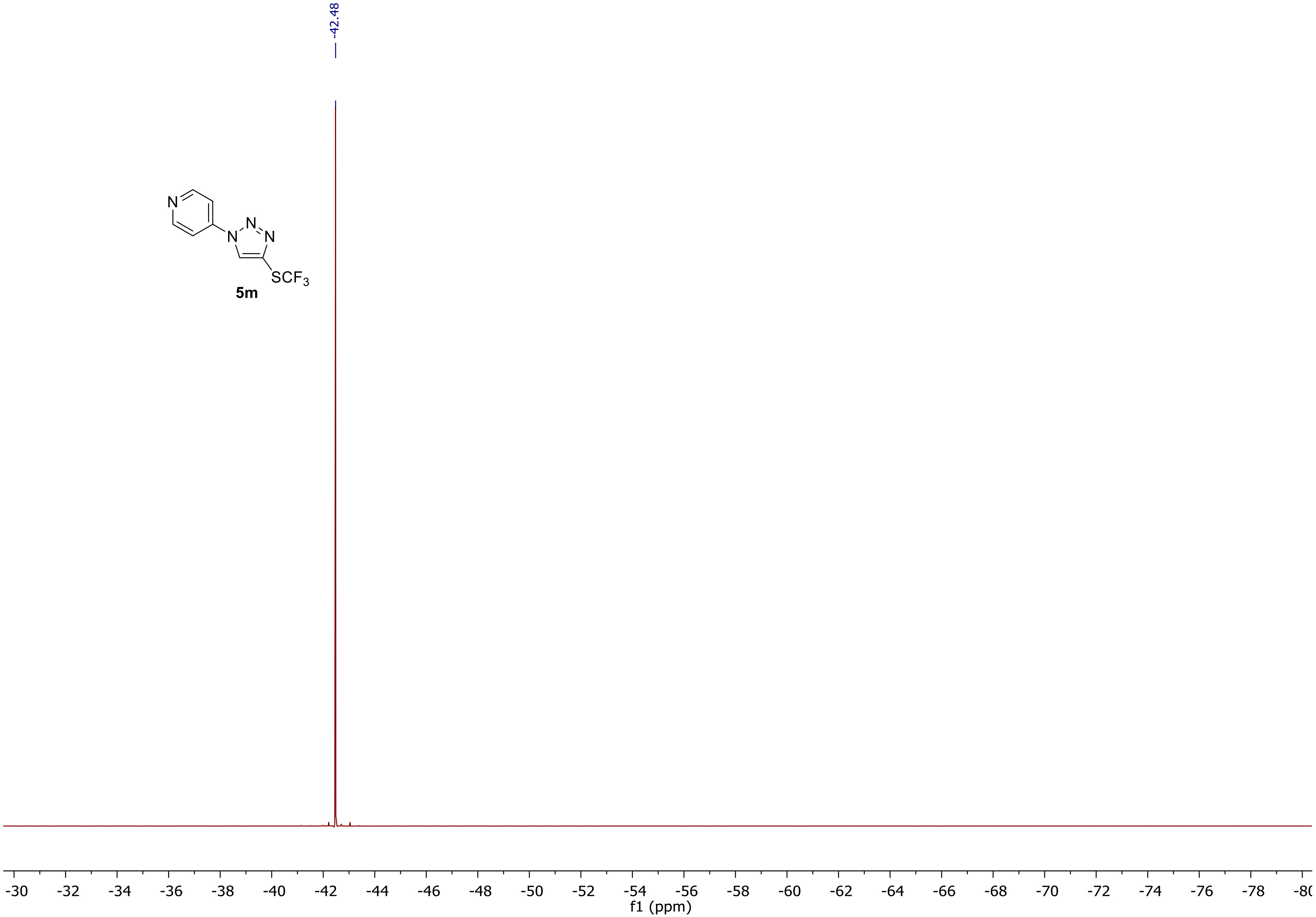

S18. ^{1}H, ^{13}C and ^{19}F NMR of Compound **5m**

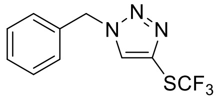
N
N
N
SCF3

5n

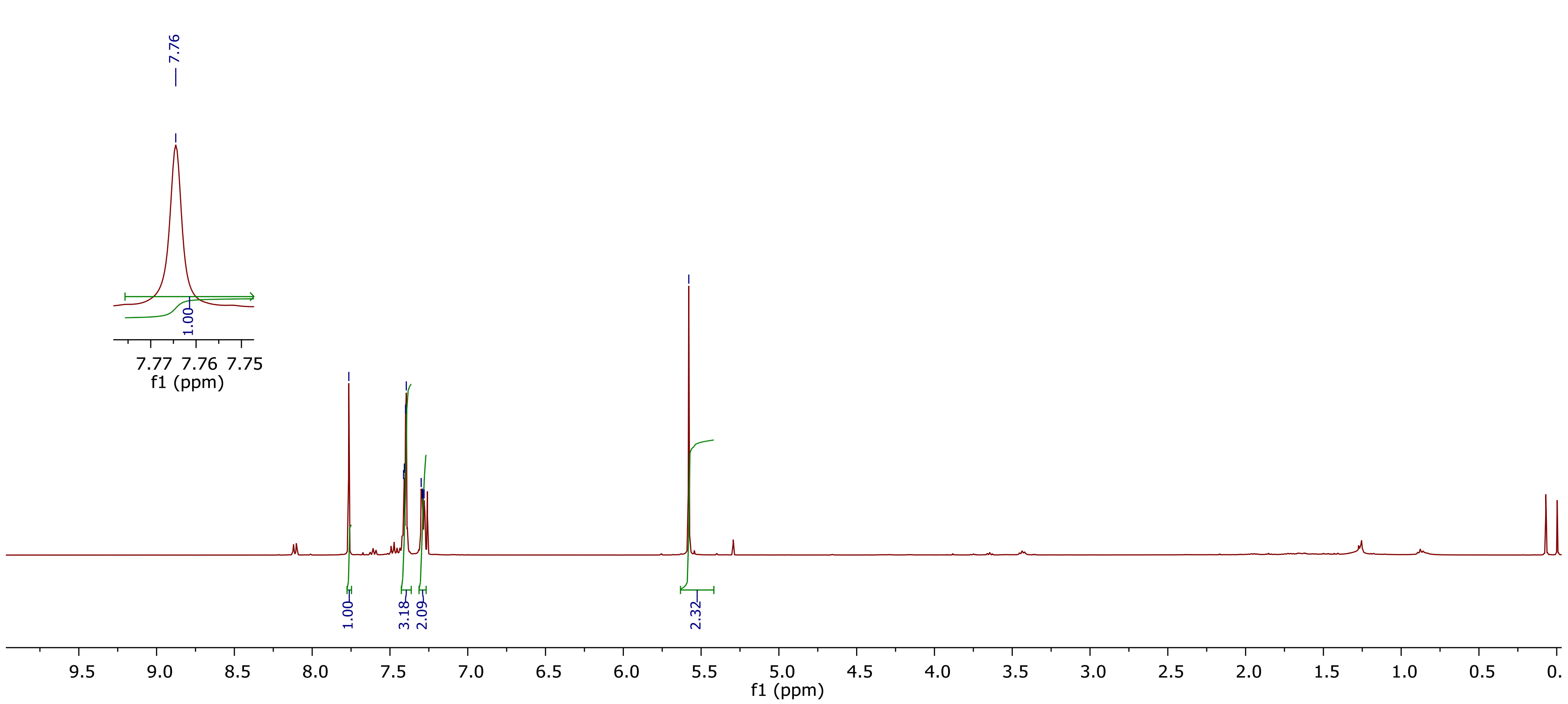
7.76
7.41
7.41
7.40
7.39
7.30
7.29
7.29
7.28
5.58
7.76
1.00
7.77 7.76 7.75
f1 (ppm)
1.00
3.18
2.09
2.32
9.5 9.0 8.5 8.0 7.5 7.0 6.5 6.0 5.5 5.0 4.5 4.0 3.5 3.0 2.5 2.0 1.5 1.0 0.5 0.
f1 (ppm)

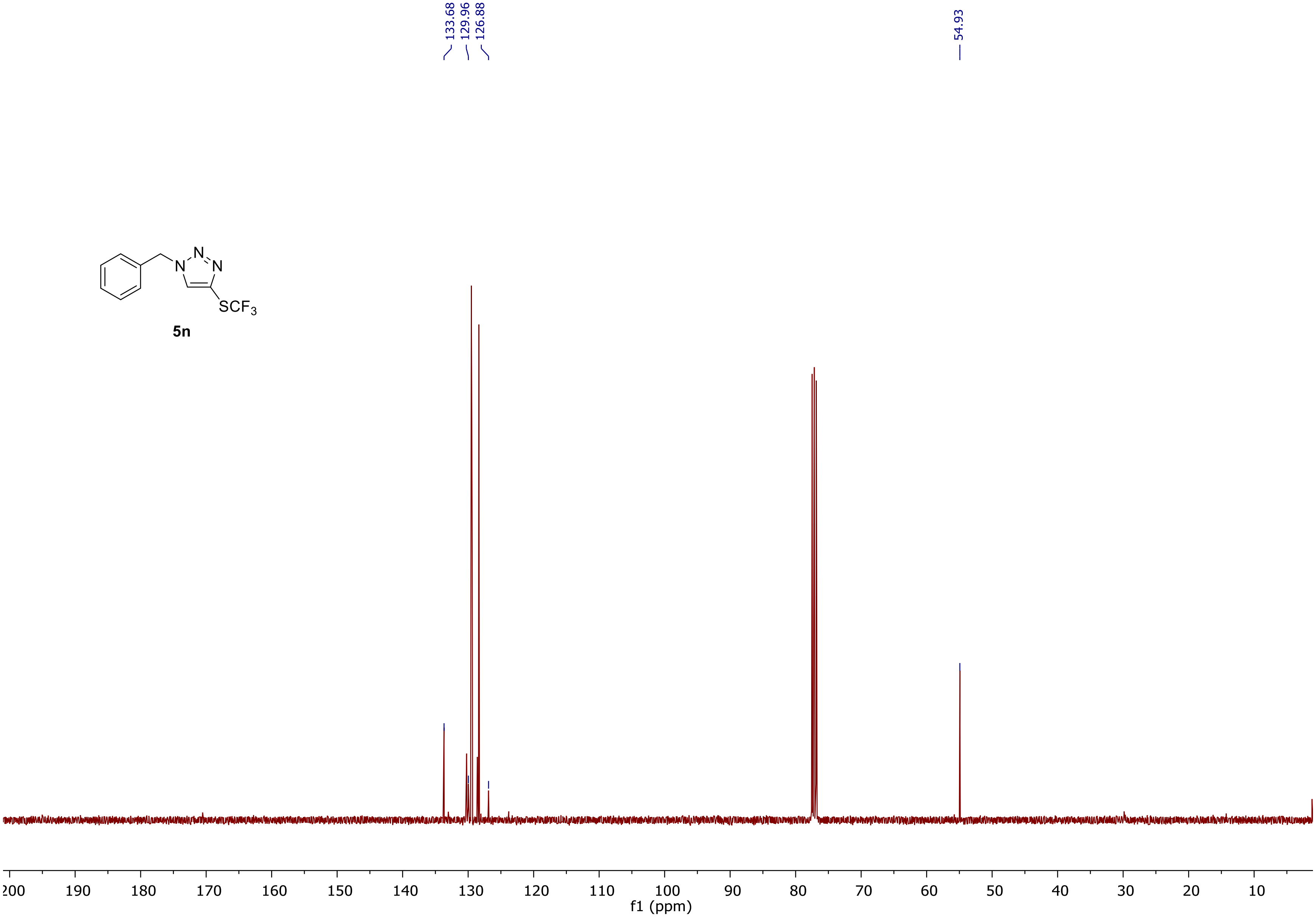

133.68
129.96
126.88
54.93
SCF3
5n
200 190 180 170 160 150 140 130 120 110 100 90 80 70 60 50 40 30 20 10
f1 (ppm)

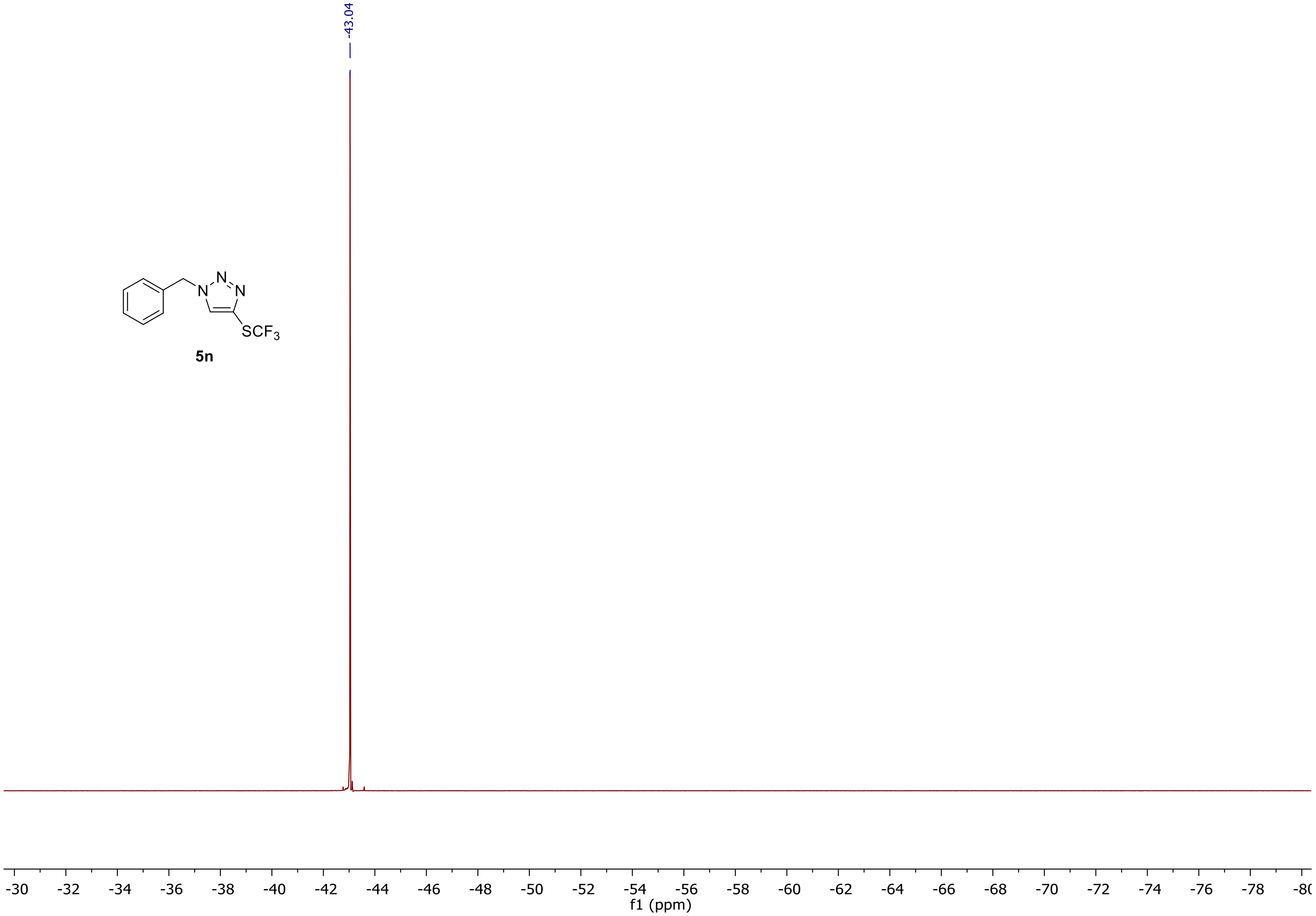

S19. ^{1}H, ^{13}C and ^{19}F NMR of Compound **5n**

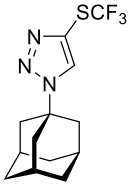
SCF3
N
N
N

5o

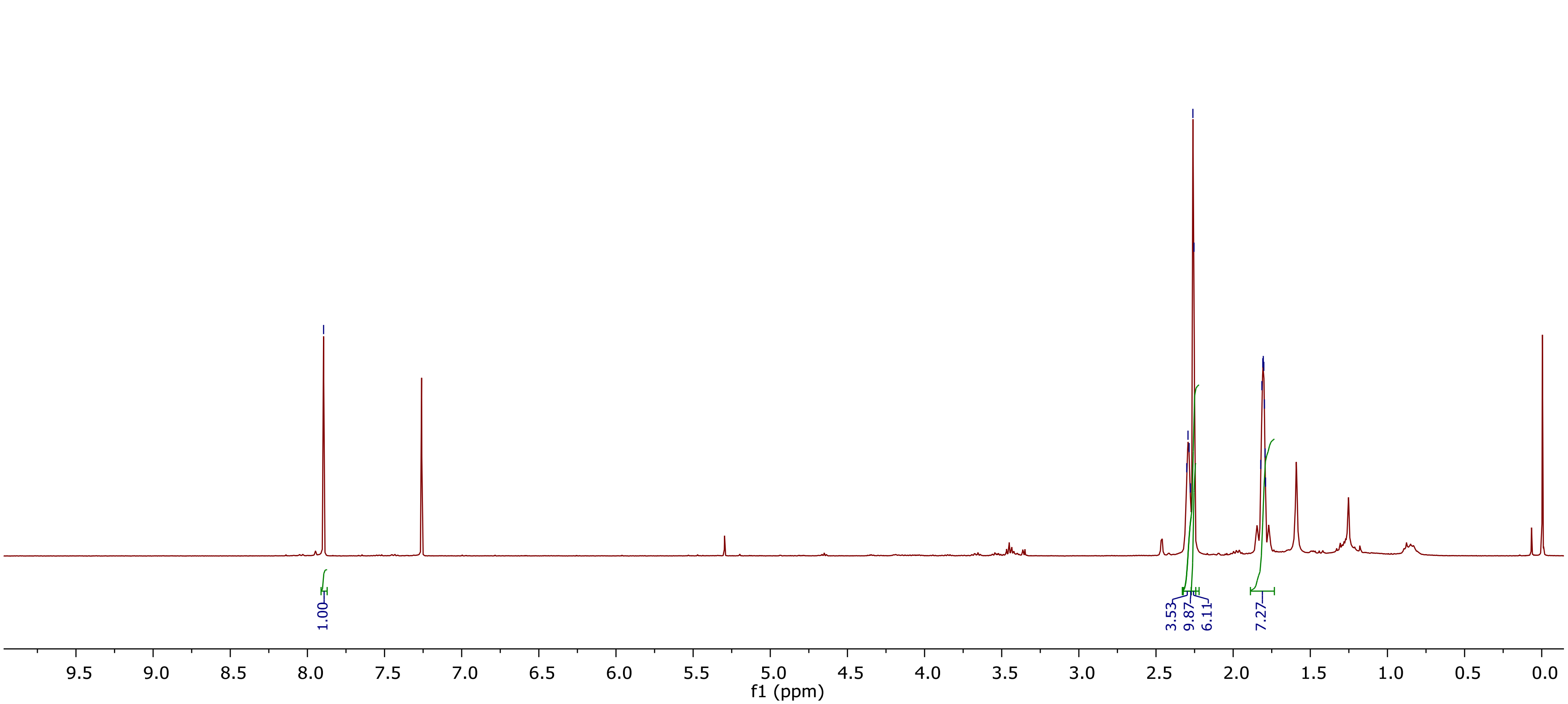
7.90
2.30
2.29
2.29
2.28
2.26
2.25
1.82
1.81
1.81
1.80
1.80
1.80
1.79
1.79
1.00
3.53
9.87
6.11
7.27
9.5
9.0
8.5
8.0
7.5
7.0
6.5
6.0
5.5
5.0
4.5
4.0
3.5
3.0
2.5
2.0
1.5
1.0
0.5
0.0
f1 (ppm)

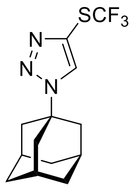
SCF3
N
N
N

5o

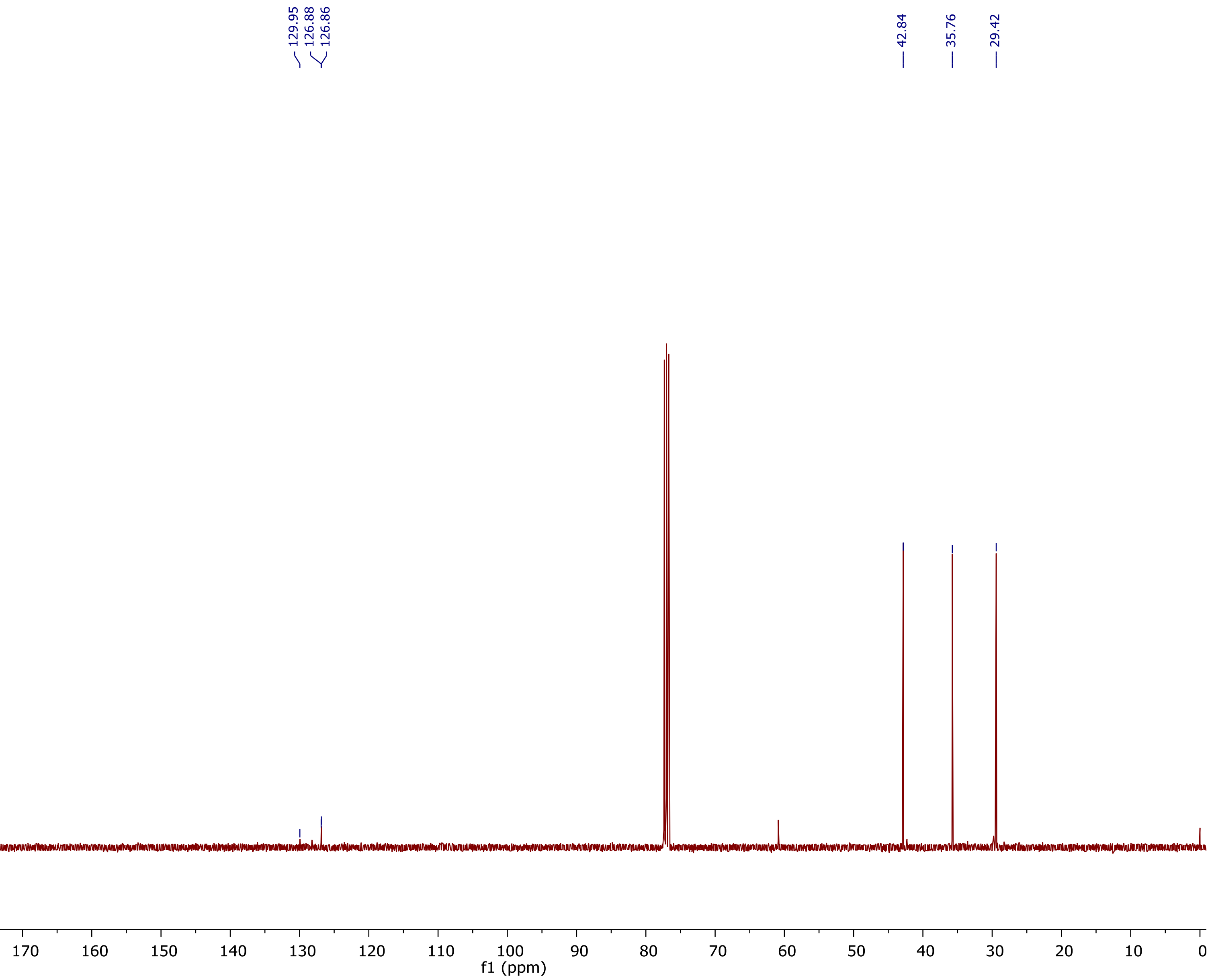
129.95
126.88
126.86
42.84
35.76
29.42
00
190
180
170
160
150
140
130
120
110
100
90
80
70
60
50
40
30
20
10
0
f1 (ppm)

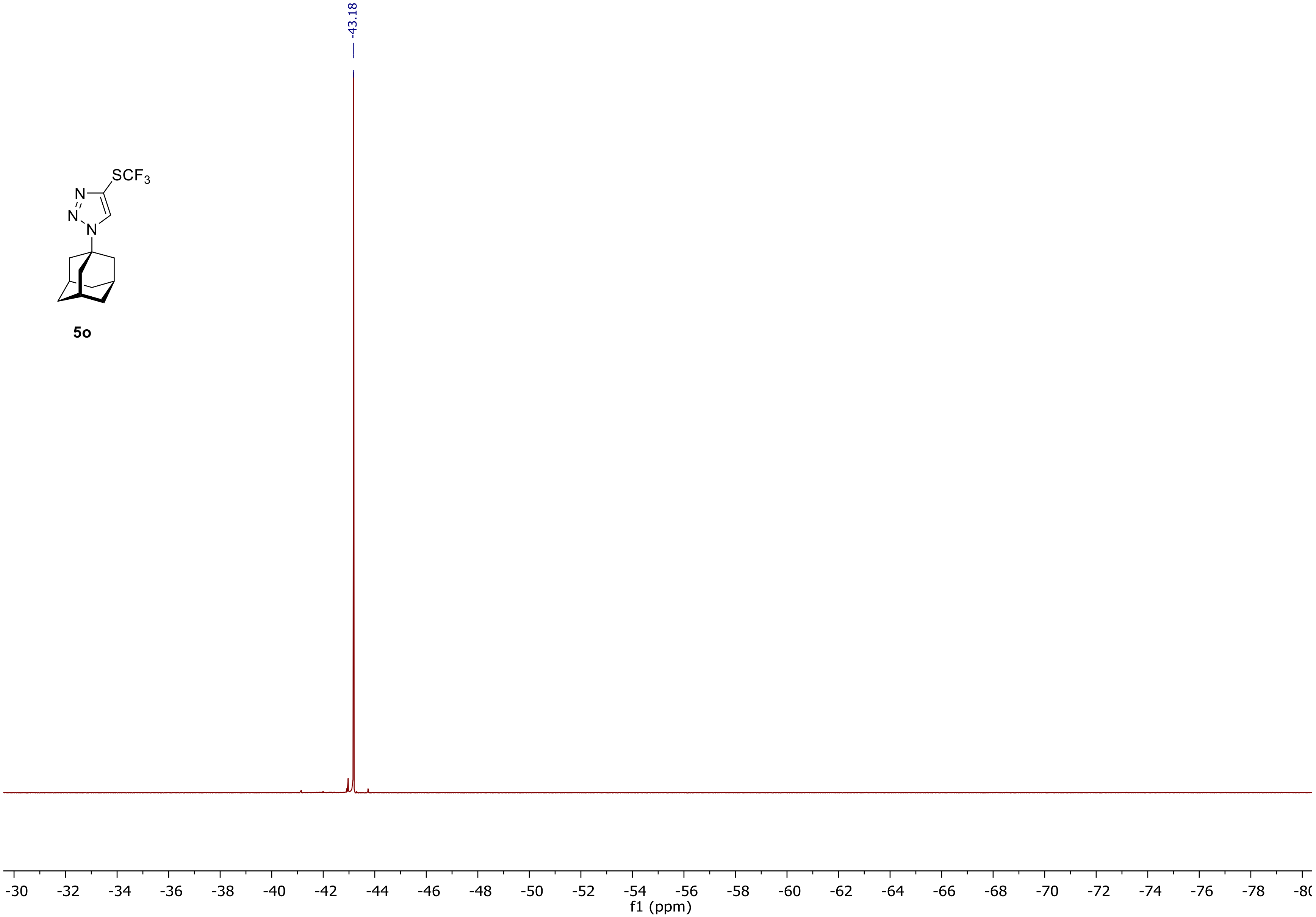

S20. ^{1}H, ^{13}C and ^{19}F NMR of Compound **5o**
